# The Role of Diet in the Prevention of Hypertension and Management of Blood Pressure: An Umbrella Review of Meta-Analyses of Interventional and Observational Studies

**DOI:** 10.1016/j.advnut.2023.09.011

**Published:** 2023-10-01

**Authors:** Ghadeer S. Aljuraiban, Rachel Gibson, Doris SM. Chan, Linda Van Horn, Queenie Chan

**Affiliations:** 1 Department of Community Health Sciences, College of Applied Medical Sciences, King Saud University, Riyadh, Kingdom of Saudi Arabia; 2 Department of Nutritional Sciences, King’s College London, London, United Kingdom; 3 Department of Epidemiology and Biostatistics, School of Public Health, Imperial College London, London, United Kingdom; 4 Department of Preventive Medicine, Northwestern University, Chicago, IL, United States

**Keywords:** blood pressure, diet, dietary patterns, hypertension, nutrients, observational studies, randomized controlled trials, umbrella review

## Abstract

High blood pressure (BP) is a major pathological risk factor for the development of several cardiovascular diseases. Diet is a key modifier of BP, but the underlying relationships are not clearly demonstrated. This is an umbrella review of published meta-analyses to critically evaluate the wide range of dietary evidence from bioactive compounds to dietary patterns on BP and risk of hypertension. PubMed, Embase, Web of Science, and Cochrane Central Register of Controlled Trials were searched from inception until October 31, 2021, for relevant meta-analyses of randomized controlled trials or meta-analyses of observational studies. A total of 175 publications reporting 341 meta-analyses of randomized controlled trials (145 publications) and 70 meta-analyses of observational studies (30 publications) were included in the review. The methodological quality of the included publications was assessed using Assessment of Multiple Systematic Reviews 2 and the evidence quality of each selected meta-analysis was assessed using NutriGrade. This umbrella review supports recommended public health guidelines for prevention and control of hypertension. Dietary patterns including the Dietary Approaches to Stop Hypertension and the Mediterranean-type diets that further restrict sodium, and moderate alcohol intake are advised. To produce high-quality evidence and substantiate strong recommendations, future research should address areas where the low quality of evidence was observed (for example, intake of dietary fiber, fish, egg, meat, dairy products, fruit juice, and nuts) and emphasize focus on dietary factors not yet conclusively investigated.

**Statement of Significance**

This umbrella review of published meta-analyses synthesized and graded the epidemiological evidence investigating the relationship between hypertension and change in blood pressure and a combination of dietary patterns, food groups, single foods, beverages, macronutrients, and micronutrients. Notably, it highlights the gap and provides a comprehensive evidence base for updating current guidelines for risk of hypertension and high blood pressure.

## Introduction

High blood pressure (BP), defined as systolic BP (SBP) of ≥130 mmHg and diastolic BP (DBP) of ≥80 mmHg [1], is a major risk factor underlying the pathogenesis of multiple cardiovascular diseases, including stroke, ischemic heart disease, heart failure, hypertensive heart disease [2], and renal events [3]. High BP was identified as the leading risk factor for adult mortality according to the 2017 Global Burden of Diseases, Injuries, and Risk Factors Study comparative risk assessment [4]. In 2019, the age-standardized global prevalence of hypertension in adults aged 30–79 y was 32% in females and 34% in males, respectively [5]; less than half of those treated had achieved hypertension control, leading to global control rates of 23% for females and 18% for males with hypertension [5]. Reducing the health burden of hypertension is, therefore, a priority for public health.

Observational studies and clinical trials report that risk factors of high BP are mainly environmental, including dietary and lifestyle factors [6,7], such as sodium and potassium intake, alcohol consumption, body weight, physical activity, socioeconomic status, and genetic factors [8], and that improvement in these factors could reduce risk of hypertension. Guidelines for the management and prevention of hypertension have been published by societies such as the European Society of Cardiology/European Society of Hypertension, International Society of Hypertension [9], and American College of Cardiology/American Heart Association [1]; all providing dietary recommendations, but primarily focusing on selected nutrients and dietary patterns, such as sodium restriction, alcohol moderation, the Mediterranean diet, and the Dietary Approaches to Stop Hypertension (DASH) diet. New evidence of other nutrients and patterns related to BP has more recently emerged but have yet to be comprehensively addressed [10].

In the past decade, evidence implicating specific macronutrients and micronutrients [11,12], single food groups [[13], [14], [15]], and dietary patterns [16,17] has been evaluated in systematic reviews and meta-analyses of randomized controlled trials (RCTs) and observational studies in relation to high BP and risk of hypertension. Some major food groups and nutrients are reportedly inversely associated with risk of hypertension; including whole grains, fruits, nuts, dairy, fiber, potassium [[11], [12], [13],^18^], whereas those positively associated include red and processed meats, dietary sugar, and sodium [15,18,19]. Other systematic reviews of RCTs have reported lower BP in individuals who conformed to specific dietary patterns, such as the DASH, Nordic, and Mediterranean diets [16,17]. While these findings offer helpful approaches for the prevention and management of high BP, the collective quality, strength, and bias of these reviews have not been well evaluated.

The synthesis of published systematic reviews, that is, an umbrella review, offers a useful method to systematically examine and compare studies’ strength of evidence and precision of estimates, producing a high-level overview of a topic [20]. Previous umbrella reviews on the relationship between diet and BP have focused solely on dietary patterns, such as the Mediterranean [21] and other popular diets [22]; single nutrients, for example, vitamin C [23], vitamin D [24], and dietary fiber [25]; single foods, such as coffee [26], garlic [27], and chocolate [28]; and certain food groups, such as pulses and legumes [29], allium vegetables [30], nuts [31], and dairy products [32]. Furthermore, some of these umbrella reviews included only observational studies [29,32] or RCTs [22,23]. To the best of our knowledge, no single umbrella review has synthesized and graded the totality of the evidence investigating the relationship between BP and a combination of dietary patterns, food groups, single foods, beverages, macronutrients, and micronutrients, that would help update and fill the gap of current guidelines. This umbrella review of meta-analyses of RCTs and observational studies evaluates the association between these dietary factors and risk of hypertension with change in BP.

## Methods

This umbrella was conducted following the Joanna Briggs Institute Umbrella Review Methodology [20]. The protocol for this umbrella review was registered on International Prospective Register of Systematic Reviews (PROSPERO) (CRD42019160516).

### Literature search

A systematic literature search was conducted in the electronic bibliography databases: PubMed (MEDLINE), Embase, Web of Science, and Cochrane Central Register of Controlled Trials from inception until October 31, 2021. Meta-analyses of RCTs or observational studies were identified through predefined and tested search terms listed in Supplemental Table 1. Studies were restricted to human studies. Two authors (GA and QC) conducted independent database searches in parallel. Extracted lists were imported into Rayyan [33] for title and abstract review by GA and QC independently. Disagreements were resolved by a third author (RG).

### Selection of meta-analyses

To avoid the inclusion of duplicate primary studies, when >1 meta-analysis was identified for a dietary exposure, we selected the most complete meta-analysis for RCTs and observational studies using the following stepwise process. In the first instance, we selected the one with the largest number of primary studies. If >1 meta-analysis had the same number of primary studies, we selected the one with the largest total sample size. If there was >1 study satisfying these criteria, the meta-analysis with the most information was selected. For each dietary exposure, where meta-analyses we excluded, these were reviewed to determine whether there was agreement on direction, magnitude, and significance of observed associations.

### Data extraction and reporting

For each meta-analysis, the following data were extracted: first author, publication year, outcome (incidence hypertension, change in BP), intervention (any administration forms), comparison group (as defined in the RCTs), exposure, number of included primary studies, number of estimates, study design of the primary studies (RCTs, cohorts, case-control, cross-sectional), numbers of participants, number of cases (observational studies), health status of participants (normotensive, hypertensive, obesity, diabetes, or a mixture of health conditions), sex of participants, type of results (for example, high versus low or dose–response and its increment unit), heterogeneity (*I*^2^), method of analysis (random or fixed effects), estimates of effect or association and their 95% confidence interval (CI) and/or *P* value, risk of bias or quality score of primary studies, publication bias, and funding source and reported conflicts of interest.

The results are reported by the themes of dietary exposure: dietary patterns, foods/beverages, macronutrients, micronutrients, and other food bioactive compounds. Within each theme the results were separated into RCTs and observational studies. Summary tables were used to present characteristics of included reviews and Forest plots were constructed to visualize effect sizes using the values extracted from the meta-analyses included in the review. Individual dietary exposures were then grouped on the basis of the NutriGrade classification of evidence quality (high, moderate, low, and very low) [34] and the direction of effect on BP (decreased, increased, and no change). Finally, dietary exposures that reported a clinically significant change in BP (>3 mmHg) were identified.

### Assessment of methodological quality

The methodological quality of the included publications was assessed with the validated Assessment of Multiple Systematic Reviews 2 (AMSTAR 2) tool [35]. Critical domains related to the conduct of the review, including the registration of a review protocol, adequacy of the literature search, justification of the excluded studies, risk of bias assessment of the included studies, appropriateness of meta-analytical methods, consideration of risk of bias in results interpretation, and presence and likely impact of publication bias, were evaluated. The overall confidence in the results of the included publications was then rated as high (no or 1 noncritical weakness), moderate (>1 noncritical weakness), low (1 critical flaw with or without noncritical weakness), or critically low (>1 critical flaw with or without noncritical weaknesses).

### Assessment of quality of evidence

Systematic reviews serve as a valuable and reliable method that provides evidence-based dietary guidance for a wide range of research questions. To increase the confidence in the observed effects, the quality of systematic reviews is evaluated using different assessment tools. NutriGrade is a numerical scoring system that is designed to grade the evidence in nutrition research [34]. It was developed on the basis of the Grading of Recommendations Assessment, Development, and Evaluation (GRADE) criteria [36], which grade the quality of evidence from systematic reviews for clinical practice. In addition to the GRADE criteria, the NutriGrade considers nutrition research-related data, such as risk of bias, directness of outcome (hard clinical outcome or surrogate markers), and funding bias when assessing the quality of evidence of meta-analyses of an association/effect between different dietary factors and outcomes.

Two reviewers (GA and RG) independently assessed the quality of each selected meta-analysis, and a third reviewer (DC) resolved conflicting scores. Six quality aspects were evaluated for both RCTs and observational studies: *1*) risk of bias, study quality, or study limitations; *2*) precision of the estimate; *3*) heterogeneity; *4*) directness; *5*) publication bias; *6*) funding bias; with *7*) study design for meta-analyses of RCTs only, and *8*) effect size and *9*) dose–response association for observational studies only. An overall score (maximum of 10 points) of 8 points, 6 to <8 points, 4 to <6 points, and <4 points represents high, moderate, low, and very low quality of evidence, respectively. A meta-analysis could be rated as high quality even when individual items such as risk of bias, heterogeneity, or publication bias did not achieve the highest score. Where the manuscript reported multiple outcomes (for example, markers of metabolic syndrome) and the authors did not separately report risk of bias or funding bias for BP or hypertension, we scored on the basis of what the authors reported for the composite of the outcomes (for example, all outcomes of metabolic syndrome). For the meta-analyses of observational studies that reported both comparing high to low dietary intake and dose–response results, NutriGrade would be scored for the results of high to low dietary intake only.

## Results

### Results of the search

**FIGURE 1:**
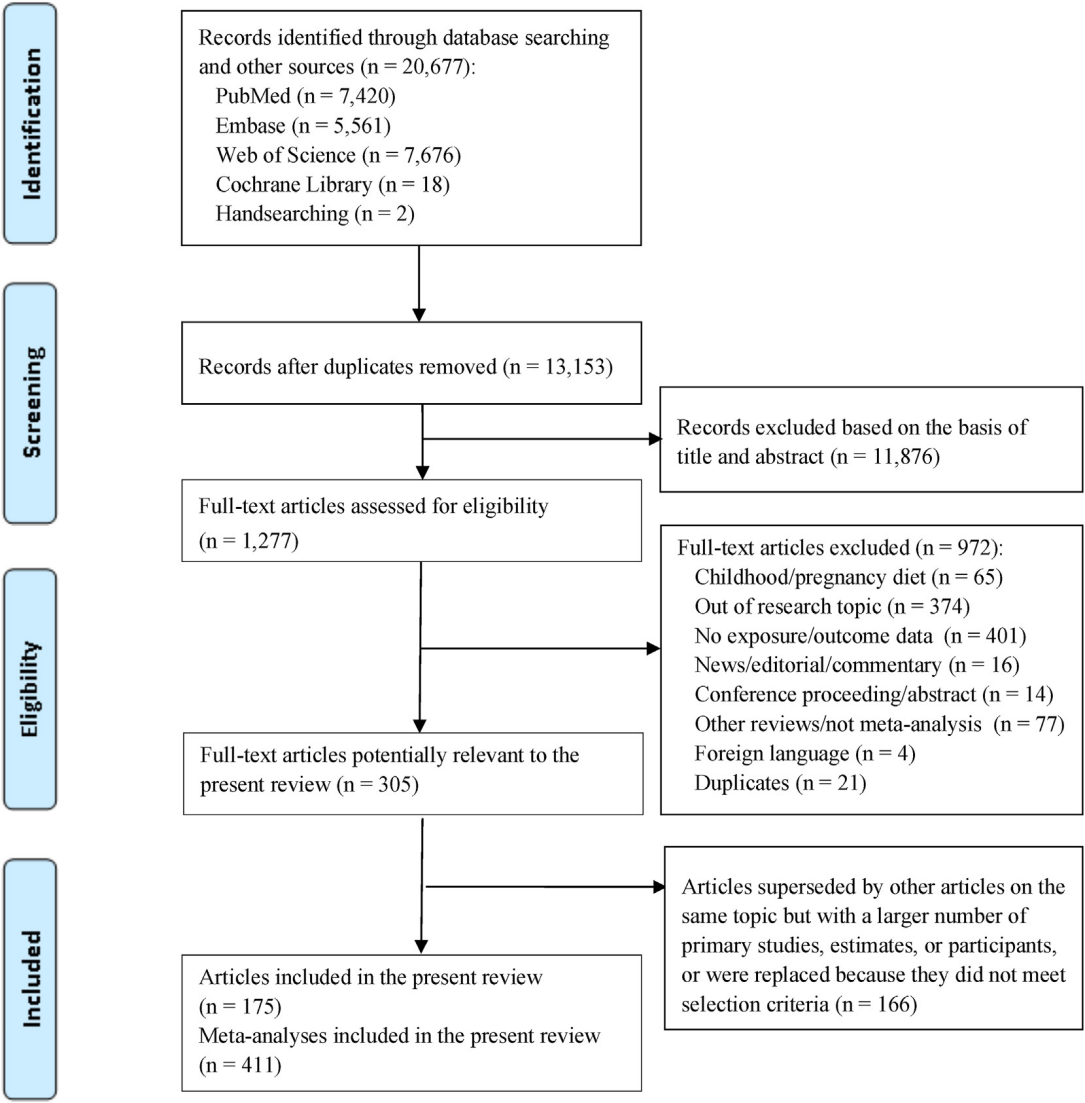
The role of diet in the prevention of hypertension and management of blood pressure: an umbrella review of meta-analyses of observational and intervention studies.

Figure 1 shows the PRISMA flowchart. Of the 17,099 records originally identified, 1277 publications were reviewed for its full text and 972 publications were subsequently excluded as they did not meet the selection criteria. Another 166 publications were superseded by other articles on the same topic but with a larger number of primary studies, estimates, or participants, or were replaced because they did not meet selection criteria, listed in Supplemental Table 2. A total of 175 publications reporting 341 meta-analyses of RCTs (145 publications) and 70 meta-analyses of diet exposures from observational studies (30 publications) were included (Supplemental Table 3).

We identified the following dietary factors: *1*) patterns of diet; *2*) foods groups: meat, poultry, fish, and egg, milk and dairy products, fruits, vegetables, fruits and vegetable, starchy foods, legumes and pulses, nuts and seeds, cocoa, herb, spice, and condiments; *3*) beverages; *4*) macronutrients: carbohydrates, sugars, proteins, fats, and oils; *5*) micronutrients: minerals, vitamins, probiotics, polyphenols, phytochemicals, nitrates, sweeteners (Supplemental Tables 4 and 5).

### Characteristics of included meta-analyses

The primary studies in the included meta-analyses were mostly from Europe (*n =* 119), North America (*n =* 106), Asia (*n =* 87), a smaller number was from the Middle East (*n =* 61), Australia and New Zealand (*n =* 50), and South America (*n =* 36), and only a few meta-analyses from Africa (*n =* 10) (Supplemental Tables 4 and 5).

For RCTs, the number of primary studies included in the meta-analyses ranged from 2 [[37], [38], [39], [40], [41], [42], [43], [44], [45]] to 91 [46], total number of participants ranged from 69 [38,39] to 36,806 [47], and duration of trial ranged between 1 h [[48], [49], [50]] and 7 y [47] (Supplemental Table 4).

For the meta-analyses of observational studies, 30 publications (70 exposures) reported results comparing high to low dietary intake [13,18,[51], [52], [53], [54], [55], [56], [57], [58], [59], [60], [61], [62], [63], [64], [65], [66], [67], [68], [69], [70], [71], [72], [73], [74], [75], [76], [77], [78]], of those 9 publications (18 exposures) also reported dose–response results [18,57,[60], [61], [62],^64^,^72^,^74^,^75^]; 3 publications (4 exposures) reported only dose–response results [13,64,67] (Supplemental Table 5). The number of primary studies included in the meta-analyses ranged from 2 [18,66,72] to 41 [68], total number of participants ranged between 5560 [76] and 640,525 [18], and the length of follow-up ranged from 1.3 [62] to 38 y [51,61,75].

### Methodological quality

Overall using AMSTAR 2, 7.5% (*n =* 11) of the included publications for RCTs were assessed as high in methodological quality, such as very-low-carbohydrate ketogenic diet compared with (Supplemental Table 4) low-fat diet [79], Nordic diet compared with standard (usual) diet [17], and high-protein diet compared with low-protein diet [80].

About 43.2% (*n =* 63) were assessed as moderate, for example, fruit juice compared with controls [81], peanuts compared with standard (usual) diet [43], and cinnamon compared with controls [82]. Low methodological quality evidence was for 27.4% (*n =* 40) of included publications, such as curcumin compared with controls [83], decaffeinated coffee compared with controls [50], and taurine supplements compared with controls [84].

Critically low methodological quality was reported in 21.9% (*n =* 32) publications, for example; soy foods compared with controls [85], calcium supplements compared with controls [86], and proanthocyanidins compared with controls [87].

For observational studies, none of the publications were rated as high quality (Supplemental Table 5). About 65.5% (*n =* 19) were rated as moderate, for example, vegan compared with omnivore [52], legumes intake [18], and total fructose intake [59]. Low methodological quality of evidence was reported for 27.6% (*n =* 8), for example, adherence to Mediterranean diet [56] and dietary long-chain n-3 polyunsaturated fatty acids (PUFA) [76], whereas critically low was reported for 6.9% (*n =* 2), for example, selenium level and SBP [63], and dietary protein intake 2 [65] (Supplemental Table 5).

The main limitations of the low- and critically low-quality meta-analyses were no grey literature, list of excluded studies, risk of bias assessment, absence of test for publication bias, and unreported source funding.

### Quality of the evidence

Using NutriGrade, 4.1% (*n =* 14) of the included meta-analyses of RCTs were rated as high and 11.4% (*n =* 39) very low and no meta-analysis of observational studies rated as high, and only (*n =* 4) were of moderate quality (Supplemental Tables 4 and 5). The main limitations identified were lack of tool for risk of bias assessment, selection bias, residual confounding, significant heterogeneity, small number of primary studies, absence of test for publication bias, and source of funding.

### Heterogeneity

Of the included meta-analyses of RCTs, 6.5% (*n =* 22) did not evaluate between-study heterogeneity, whereas 35.4% (*n =* 121) of the meta-analyses reported significant heterogeneity defined by (*P* < 0.05) and 50.7% (*n =* 173) reported heterogeneity defined by *I*^2^ ≥ 40% (Supplemental Table 4). For observational studies, 7.1% (*n =* 5) did not evaluate heterogeneity, whereas 42.9% (*n =* 30) of the meta-analyses reported significant heterogeneity defined by (*P* < 0.05) and 50.0% (*n =* 35) reported heterogeneity defined by *I*^2^ ≥ 40% (Supplemental Table 5).

### Publication bias

For RCTs, only 29.6% (*n =* 101) of the meta-analyses had 10 or more studies and reported no evidence of publication bias (low risk), 33.4% (*n =* 114) either had 5–9 studies and reported no evidence for publication bias or had 10 or more studies but reported moderate or small amount of publication bias (moderate risk), and 35.2% (*n =* 120) either had low number of primary studies, evidence for severe publication bias, or did not assess publication bias (unclear or severe risk).

Of the high and moderate evidence quality meta-analyses, (*n =* 10) and (*n =* 70) were of low risk of publication bias, respectively, and the interventions under reviewed were (high quality): very-low-carbohydrate ketogenic diet compared with low-fat diet (DBP) [79], DASH diet compared with controls [88], flaxseed compared with controls [89], multivitamin and multimineral compared with controls (DBP) [90], products with live bacteria compared with controls [91], grape and its products compared with controls [92], nitrates compared with controls (SBP) [93], urinary potassium compared with controls [94], and steviol glycoside compared with controls (DBP) [95].

For observational studies, 14.2% (*n =* 10) of the meta-analyses were of low risk, 32.9% (*n =* 23) moderate risk, and 57.1% (*n =* 40) unclear or severe risk of publication bias.

### Evidence of diet, blood pressure, and hypertension

Here, we describe the findings in the meta-analyses and their evidence quality, focusing more on high- and moderate-quality evidence assessed by NutriGrade, alongside low- or very-low-quality evidence. The NutriGrade ratings for a dietary factor were often the same for SBP and DBP and the evidence was generally for populations of mixed health statuses, the exceptions were specified. We summarized the effects of diet exposure on blood pressure and risk of hypertension in Table 1 [[11], [13], [15], [17], [18], [19], [37], [38], [39], [40], [41], [42], [43], [44], [45], [46], [47], [48], [49], [50], [51], [54], [55], [56], [57], [58], [59], [60], [62], [66], [67], [71], [72], [73], [74], [75], [76], [77], [79], [80], [81], [82], [85], [86], [87], [88], [89], [90], [91], [92], [93], [94], [95], [96], [97], [98], [99], [100], [101], [102], [103], [104], [105], [106], [107], [108], [109], [110], [111], [112], [113], [114], [115], [116], [117], [118], [120], [121], [122], [123], [124], [125], [126], [127], [128], [129], [130], [131], [132], [133], [134], [135], [136], [137], [138], [139], [141], [142], [143], [145], [146], [147], [148], [149], [150], [151], [153], [154], [155], [156], [157], [158], [159], [160], [161], [162], [163], [164], [165], [166], [167], [168], [169], [170], [171], [172], [173], [174], [175], [176], [177], [178], [179], [180], [181], [182], [183], [184], [185], [186], [187], [188], [189], [190], [191], [192], [193], [194], [195], [196], [197], [198], [199], [200], [201], [202], [203], [204], [205], [206], [207], [208], [209]]. The study characteristics, weighted mean difference, weighted mean change difference, or standardized mean difference in SBP or DBP or relative risk estimates for risk of hypertension are shown in Supplemental Table 3 for RCTs, Supplemental Table 4 for observational studies; NutriGrade and AMSTAR 2 ratings of the meta-analyses are presented in Supplemental Tables 3 and 4.

**TABLE 1:** Summary of effects on blood pressure and risk of hypertension by study type and diet exposure

(a) BP effects of diet exposure in randomized control trials by NutriGrade				
NutriGrade	Diet exposure	Decreased BP	Increased BP	No effect (NS)
High	Patterns of diet	Very-low-carbohydrate ketogenic diet vs. low-fat diet (DBP) [79] DASH diet^1^ [88]		
	Nuts and seeds	Flaxseed^2^ [89]		
	Minerals	Urinary potassium^1^ [94]		
	Vitamins			Multivitamin and multimineral (DBP) [90]
	Probiotics	Products with live bacteria [91]		
	Polyphenols	Grape and its products [92]		
	Nitrates	Nitrates (SBP) [93]		
	Sweeteners	Steviol glycoside (DBP) [95]		
Moderate	Patterns of diet	Low-carbohydrate diet vs. controls [102] Mediterranean diet [103] Dietary interventions^1^, low-calorie/fat diet, low-sodium diet, low-sodium and low-calorie/fat diet, low sodium/high potassium^1^, low-calorie/fat diet 1 [100] Plant-based diet [104] Vegetarian [105] High-protein diet [80] High-MUFA diet [101]		Very-low-carbohydrate ketogenic diet (SBP) [79] Vegan diet [106] Hyperproteic diet [107] Low-carb and high-fat diet, low-carb and high-protein diet [44] Low-fat high-protein diet (DBP) [108] Low-fat high-carbohydrate diet [109] Mediterranean diet [100]
	Meat, poultry, egg, and fish			Fish [117] Egg [118]
	Milk and dairy	Lactotripeptides [120]		
	Fruits and vegetables	Blueberry (DBP) [122] Berries^3^ [124] Cactus pear^3^ [45] Pomegranate^1^^,^^2^^,^^3^ [125] Beetroot^1^^,^^3^ [123]		Blueberry (SBP) [122] Strawberry (SBP)^2^ [127] Bitter melon [126]
	Legumes and pulses	Pulses (SBP) [142]		Pulses (DBP) [142]
	Nuts and seeds	Sesame [143] Nigella seeds^1^ [143]		Walnuts [145]
	Cocoa	Cocoa products [149]		
	Herb, spice, and condiment	Cinnamon^1^ [82]	Licorice (DBP) [150]	Licorice (SBP) [150]
	Beverages	Alcohol reduction^1^ [153] Black tea [49] Green tea [96]		Coffee [154]
	Carbohydrates	Psyllium [156] Inositol [157]		Inulin [158]
	Sugars	Fructose (DBP) [161]	Free sugars (DBP) [15]	Fructose (SBP) [161] Free sugars (SBP) [15]
	Proteins	L-carnitine supplements (DBP) [162] Soy protein^2^, soy protein isolate^2^ [85] Dietary peptides (SBP)^1^^,^^2^ [163]		Soy foods^2^ [85]
	Fats and oils	EPA and/or DHA supplements^2^ [169]		Olive oil supplements^2^ [170]
	Minerals	Sodium or salt substitute^1^ [19] Magnesium supplements [176]		Chromium supplements [177]
	Vitamins	Folic acid supplements [97] Vitamin C supplements^1^ [184] Multivitamin and multimineral (SBP) [90] Vitamin D supplements [185]		Vitamin D3 supplements^2^ [186] Vitamin D + Calcium [47]
	Probiotics	Lactobacillusplantarum supplements 1 [190]		Fermented milk^2^ [191]
	Polyphenols	Grape seed extract (SBP)^2^ [192] Quercetin (DBP) [194] Flavanols [46] Proanthocyanidins [87]		Resveratrol^2^ [196] Anthocyanin supplements (SBP)^2^ [195] Grape seed extract (DBP)^2^ [192]
	Phytochemicals	Phytosterols [202] Green coffee extract^1^ [203] Green tea extract (DBP) [204] Chlorogenic acid^1^ [205]		Green tea extract (SBP) [204] Tea extract [204] Lycopene supplements [206]
	Nitrates	Nitrates (DBP)^1^^,^^2^ [93]		
	Sweeteners	Stevioside^1^ [95]		Steviol glycoside (SBP), pure rebaudioside [95]
	Others	Astaxanthin supplements [209]		
Low	Patterns of diet	Portfolio diet [111] Paleolithic diet^1^ [110] Caloric restriction diet [112] Nordic diet^1^ [17]		Low-fat high-protein diet (SBP) [106] High carb diet [113]
	Meat, poultry, egg, and fish			
	Milk and dairy			Whole-fat dairy products, low-fat dairy [121]
	Fruits and vegetables	Strawberry (DBP) [127] Sour cherry (DBP) [132] Fruit juice (DBP) [81] Grapefruit (SBP)^3^ [131] Cranberry (SBP)^1^^,^^3^ [130] Ginger^1^^,^^2^^,^^3^ [129] Garlic^1^^,^^2^^,^^3^ [128] Fruit and vegetable^3^ [136]		Goji berry^3^ [133] Sour cherry (SBP) [132] Fruit juice (SBP) [81] Orange juice [135] Grapefruit (DBP)^3^ [131] Cranberry (DBP)^1^^,^^3^ [130] Kiwi fruit^3^ [134] Tomato [137]
	Starchy foods			Whole grains [141]
	Legumes and pulses			Peanuts, soy nuts [43]
	Nuts and seeds	Pistachios (SBP) [146] Almonds (DBP) [147] Cashew (SBP) [41]		Pistachios (DBP) [146] Mixed nuts vs. standard (usual) diet, tree nuts (DBP) [37] Almonds (SBP) [147] Cashew (DBP) [41] Mixed nuts vs. controls (SBP) [43] Chia seed [148]
	Herb, spice, and condiment	Curcumin (SBP) [98]		Curcumin (DBP) [98]
	Beverages	Decaffeinated coffee [50] Sour tea^1^ [155]		Beer [99] Tea [48]
	Carbohydrates	Dietary fiber (DBP) [160]		Arabinoxylan-rich foods^2^ Mannans^2^ [11] B-glucan (DBP), Guar gum (DBP) [42] Dietary fiber (SBP) [160]
	Proteins	Whey protein supplements^1^^,^^2^ [167] Milk protein^1^ [165] Dietary peptides (DBP) [163] Dietary protein^2^ [164] Soy milk [166]		L-carnitine supplements (SBP) [162] L-citrulline supplements [168] Dietary animal proteins 2 [164]
	Fats and oils	EPA supplements (SBP) [171] Fish oil supplements^1^^,^^2^ [172]		DHA supplements, EPA supplements (DBP) [171] CLA supplements [173]
	Minerals	Calcium supplements (SBP)^2^ [86] Dietary and supplemental calcium [179] Low vs. high urinary sodium to potassium ratio [178] Potassium supplements^2^ [180]		Potassium and magnesium supplements [181]
	Vitamins	Vitamin E supplements (SBP)^1^^,^^2^ [189]		Vitamin E supplements (DBP)^1^^,^^2^ [189] Active vitamin D^2^ [187] Folate supplements [188]
	Polyphenols	Anthocyanins (DBP) [193] High-phenolic olive oil (SBP) [39] Grape polyphenols (SBP) [197] Quercetin^1^ [194] Flavonoid-rich cocoa (SBP)^2^ [199] Red wine polyphenols (SBP) [200]		Flavonoid-rich fruits (hypertension population only) [201] Hydrobenzoic acids^2^ [193] High-phenolic olive oil (DBP) [39] Grape polyphenols (DBP) [197] Hesperidin [198] Flavonoid-rich cocoa (DBP)^2^ [199] Red wine polyphenols (SBP) [200]
	Phytochemicals	Olive leaf extract (SBP) [40]		Olive leaf extract (DBP) [40]
Very low	Patterns of diet	High fruit and vegetable diet (DBP) [114]		Low AGE diet vs. high AGE diet [116] Breakfast skipping [38] Low-carb diet vs. low-fat diet^2^ [115] High fruit and vegetable diet (SBP) [114]
	Meat, poultry, egg, and fish			Total meat
	Fruits and vegetables	100% fruit juice [138] Aronia berry (SBP) [139]		Aronia berry (DBP) [139]
	Nuts and seeds			Tree nuts (SBP) [37] Chia seed (SBP) [148]
	Herb, spice, and condiment	Saffron [151]		
	Carbohydrates	Chitosan [159]		B-glucan (SBP), guar gum (SBP), Konjac glucomannan, and pectin [42]
	Fats and oils	Dietary and supplemental EPA and/or DHA^2^ [175]		ALA supplements^2^ [174]
	Minerals	Urinary sodium^2^ [183]		Calcium supplements (DBP) [86] Zinc supplements [182]
	Phytochemicals	Genistein (SBP) [208]	Caffeine^1^^,^^2^ [207]	Genistein (DBP) [208]

(b) Risk of hypertension (relative risk, RR or odd ratio, OR) diet exposure in observation studies by NutriGrade				
NutriGrade	Diet exposure	Lower risk	Higher risk	No association (NS)
Moderate	Milk and dairy	Dairy products, low-fat dairy products, milk intake, fermented dairy intake [58]		
Low	Patterns of diet		DII diet [54]	
	Meat, poultry, egg, and fish	Egg [77]	Total meat [77]	
	Milk and dairy			Whole-fat dairy products, yogurt, and cheese [58]
	Fruits and vegetables	Fruits, vegetables, fruits, and vegetables [74]		
	Beverages	Coffee [75]	Alcohol [71] Sugar-sweetened soda [61]	
	Minerals	Dietary and supplemental calcium [60]		
	Vitamins	25-hydroxyvitamin D [62]		
	Polyphenols			Flavan-3-ol^2^, flavanones^2^ [55]
Very low	Patterns of diet	Mediterranean diet [56] Healthy pattern [73]	Acid load (PRAL) diet [67]	Acid load (NEAP) diet [67]
	Meat, poultry, egg, and fish		Unprocessed red meat, processed red meat [18] Poultry [77]	Fish [18]
	Milk and dairy	Fluid dairy products [69] Low-fat dairy^2^ [13]		
	Starchy foods	Whole grains [18]	French fries^2^ [72]	Refined grains [18] Baked/boiled/mashed potatoes^2^, potatoes^2^ [72]
	Legumes and pulses	Legumes [18]		
	Nuts and seeds	Nuts [18]		
	Cocoa			Chocolate [66]
	Beverages		Artificial-sweetened beverages [51,61]	
	Sugars			Fructose [59]
	Fats and oils	Biomarker of long-chain PUFA, total long-chain PUFA [76]		Dietary long-chain PUFA [76]
	Minerals	Dietary magnesium [57]		Serum magnesium [57]
	Vitamins	25-hydroxyvitamin D [62]		Dietary vitamin D [62]
	Polyphenols	Anthocyanin^2^ [55]		Flavones^2^, flavonols^2^, total flavonoids^2^ [55]

Abbreviations: AGE, advanced glycation end products; ALA, alpha-lipoic acid; AMSTAR, Assessment of Multiple Systematic Reviews; CLA, conjugated linoleic acid; DASH, Dietary Approaches to Stop Hypertension; DII, dietary inflammatory index; hypertension, hypertensive people only; NEAP, net endogenous acid production; PRAL, potential renal acid load.

1 Blood pressure change ≥3 mmHg.

2 Poor-quality meta-analyses (AMSTAR low or critically low).

3 Extract, juice, powder, paste, and supplementation.

### Patterns of diet—RCTs

**FIGURE 2:**
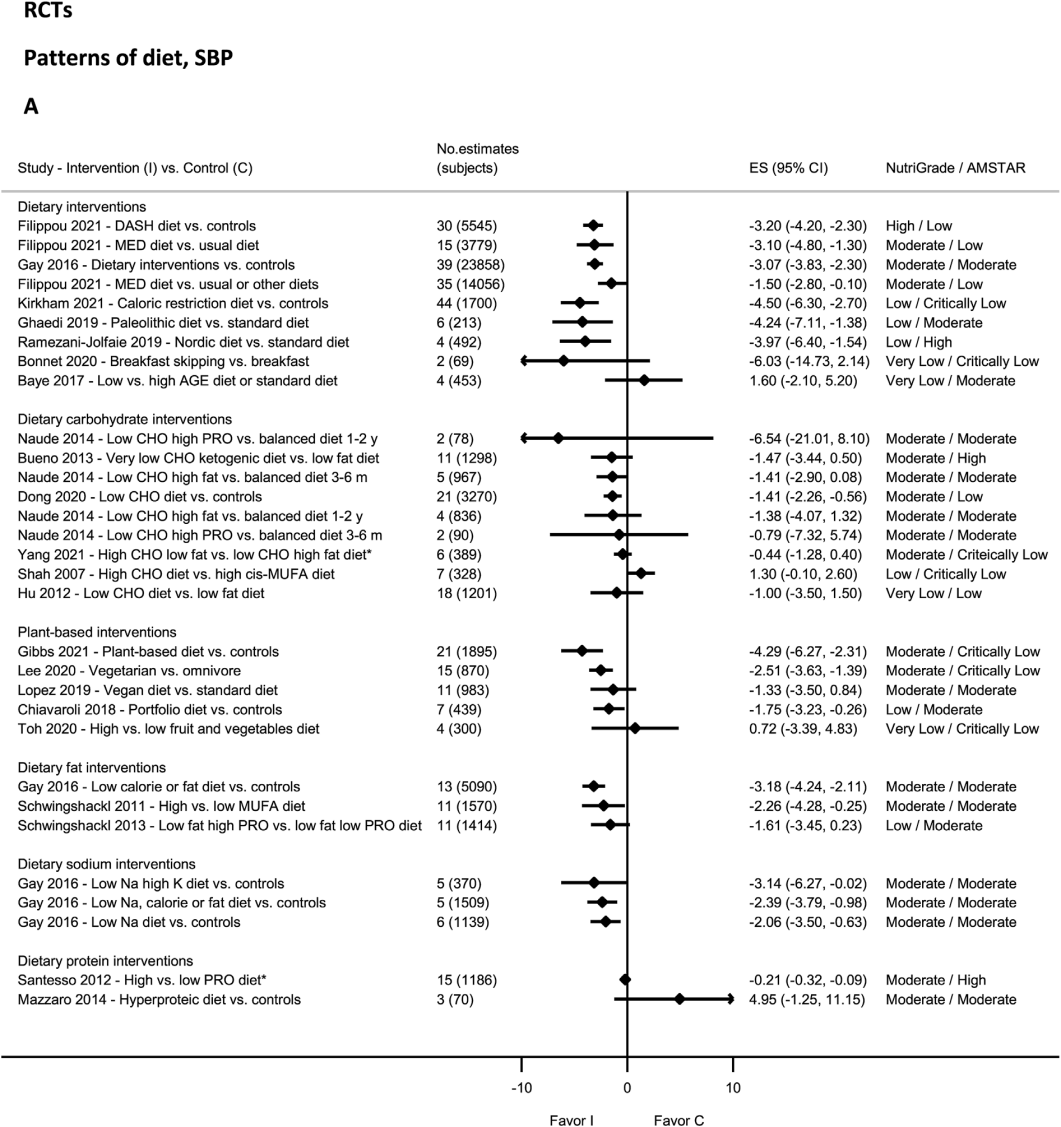

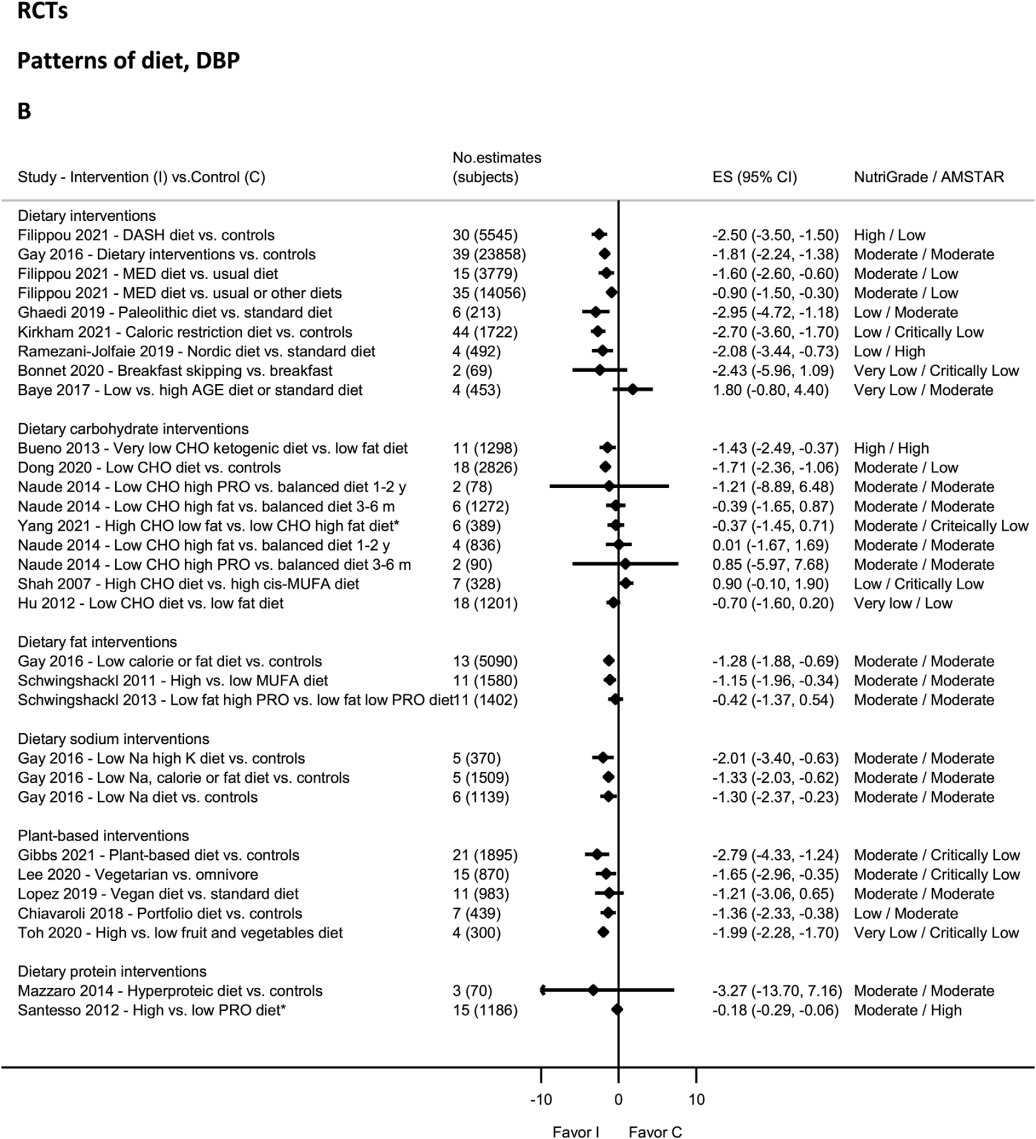
(A) Summary mean difference (ES), evidence quality (NutriGrade), and methodological quality (AMSTAR) of meta-analyses of randomized controlled trials investigating the effects of dietary patterns on systolic blood pressure. (B) Summary mean difference (ES), evidence quality (NutriGrade), and methodological quality (AMSTAR) of meta-analyses of randomized controlled trials investigating the effects of dietary patterns on diastolic blood pressure. Each solid diamond and the horizontal line across the diamond represents the summary mean difference or summary standardized mean difference (∗) and its 95% confidence interval reported by the published meta-analysis. AGE, advanced glycation end products; AMSTAR, Assessment of Multiple Systematic Reviews; CHO, carbohydrate; C, control; DASH, Dietary Approaches to Stop Hypertension; ES, mean difference estimate; I, intervention; K, potassium; MED, Mediterranean; Na, sodium; PRO, protein.

High-quality evidence on NutriGrade showed significantly decreased SBP and DBP with the DASH diet [88] and DBP with the very-low-carbohydrate ketogenic diet (Figure 2A and B) [79]. Moderate-quality evidence showed statistically significantly decreased SBP and DBP with dietary interventions [80,100,101], in particular for low-sodium diets with high-potassium [100], low-calorie or fat diets [100], low-carbohydrate diet [102], the Mediterranean diet [103], high-monounsaturated fatty acids (MUFA) diet [101], plant-based diet [104], and vegetarian diet [105]. Moderate-quality evidence showed nonsignificantly decreased SBP and DBP with other dietary patterns [44,79,[106], [107], [108], [109]] including vegan diet [106], a low-fat, high-protein diet (DBP) [108], and a low-carb, high-protein diet [44]. The evidence was rated as low quality for Paleolithic diet [110], Nordic diet [17], portfolio diet [111], caloric-restricted diet [112], low fat, high-protein diet (SBP) [108], high-carbohydrate diet [113] and very low quality for high fruit and vegetable diet [114], low-carb diet [115], breakfast skipping [38], and low advanced glycation end products [116].

### Patterns of diet—observational studies

**FIGURE 3:**
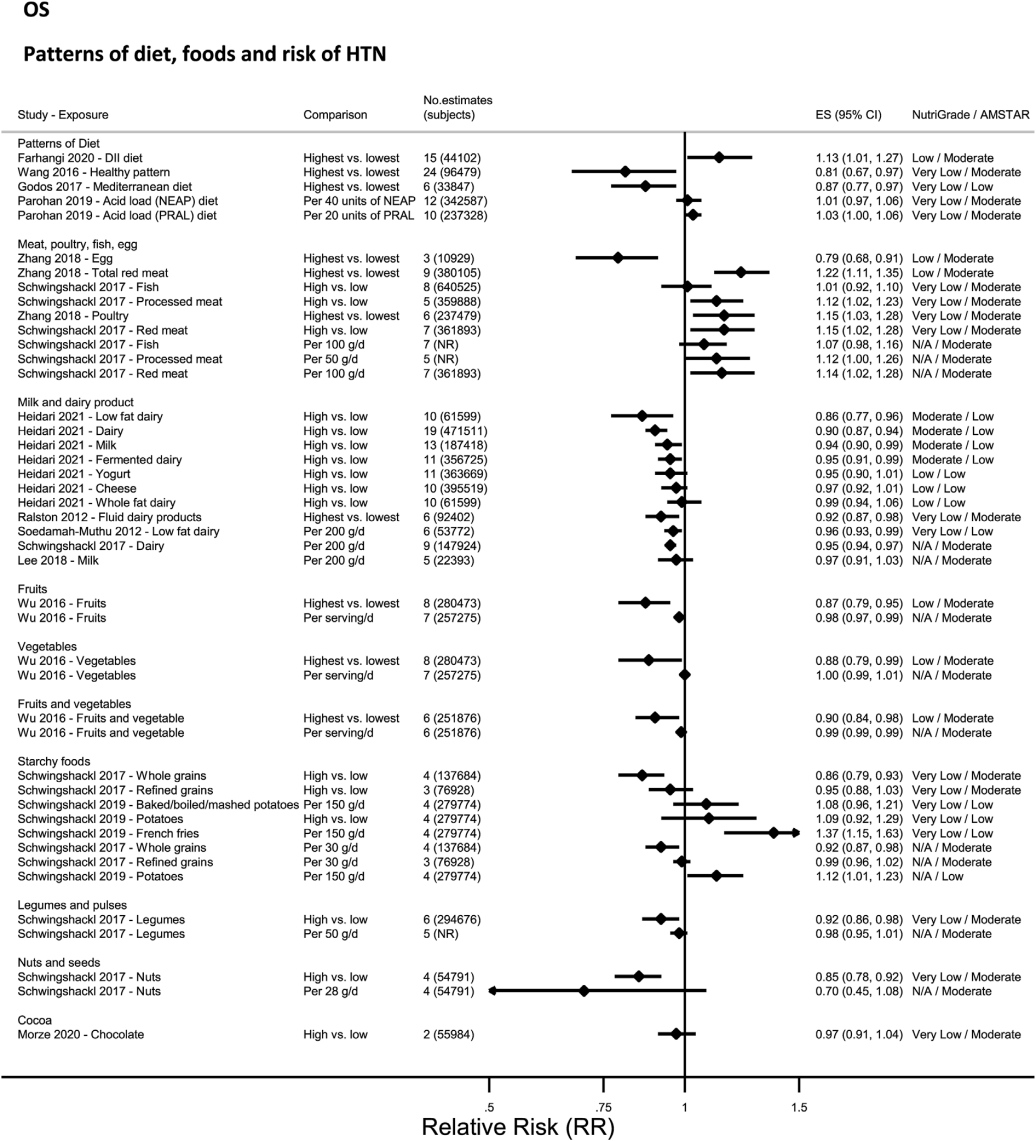
Summary relative risk (ES), evidence quality (NutriGrade), and methodological quality (AMSTAR) of the meta-analyses of observational studies investigating dietary patterns and foods in relation to risk of hypertension. Each solid diamond and the horizontal line across the diamond represents the summary relative risk and its 95% confidence interval for high vs. low comparison or per unit increment of exposure reported by the published meta-analysis. AMSTAR, Assessment of Multiple Systematic Reviews; DII, dietary inflammatory index; NEAP, net endogenous acid production; PRAL, potential renal acid load.

No evidence was rated as high- or moderate-quality evidence by NutriGrade (Figure 3). Only low-quality or very-low-quality evidence was available for vegan diet [52], diets characterized by the dietary inflammatory index [54], or empirically [73], Mediterranean diet [56], and acid load diets [53,67], and vegetarian diet [68].

### Meat, eggs, and fish—RCTs

**FIGURE 4:**
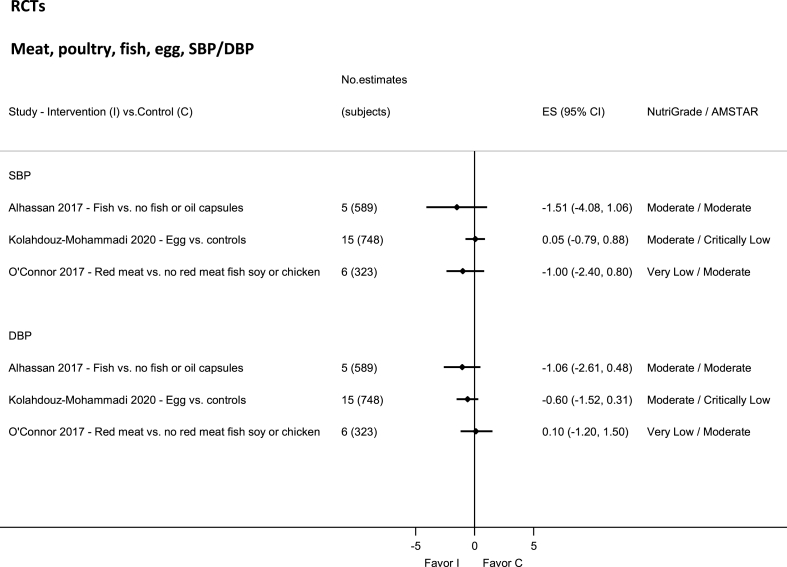
Summary mean difference (ES), evidence quality (NutriGrade), and methodological quality (AMSTAR) of the meta-analyses of randomized controlled trials investigating the effects of meat, poultry, fish, and egg on systolic and diastolic blood pressure. Each solid diamond and the horizontal line across the diamond represents the summary mean difference and its 95% confidence interval reported by the published meta-analysis. AMSTAR, Assessment of Multiple Systematic Reviews; C, control; ES, mean difference estimate; I, intervention.

No evidence was rated as high quality by NutriGrade (Figure 4). There was moderate-quality evidence showing nonsignificantly decreased SBP and DBP with fish intake [117], and egg [118], and very-low-quality evidence for total red meat [119].

### Meat, eggs, and fish—observational studies

No evidence was rated as high or moderate quality by NutriGrade (Figure 3). There was only low-quality evidence for egg and total meat [77], and very-low-quality evidence for poultry [77], and from 1 meta-analysis on processed and unprocessed meat, red meat, and fish [18].

### Dairy—RCTs

**FIGURE 5:**
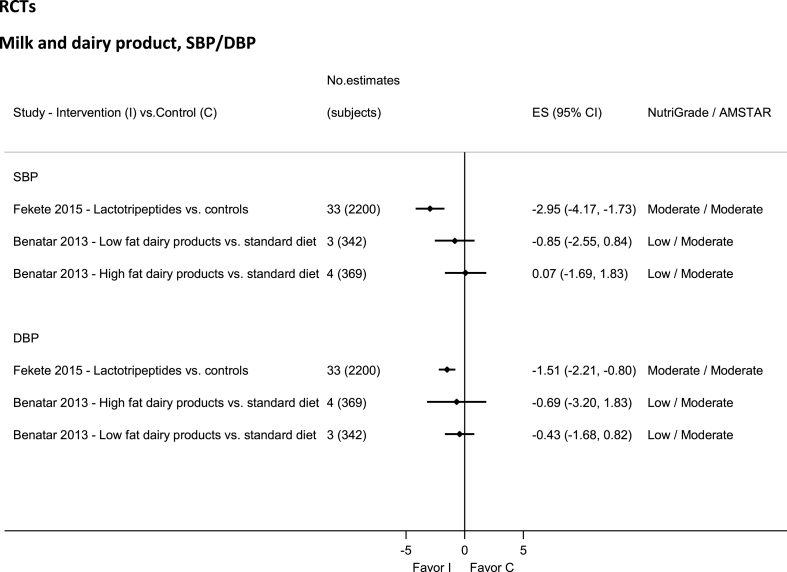
Summary mean difference (ES), evidence quality (NutriGrade), and methodological quality (AMSTAR) of the meta-analyses of randomized controlled trials investigating the effects of milk and dairy products on systolic and diastolic blood pressure. Each solid diamond and the horizontal line across the diamond represents the summary mean difference and its 95% confidence interval reported by the published meta-analysis. AMSTAR, Assessment of Multiple Systematic Reviews; C, control; ES, mean difference estimate; I, intervention.

No evidence was rated high or very low quality by NutriGrade (Figure 5). Moderate-quality evidence showed significantly decreased SBP and DBP with lactotripeptides intake [120]. There was low-quality evidence for whole and low-fat dairy [121].

### Dairy—observational studies

No evidence was rated as high quality by NutriGrade (Figure 3). There was moderate-quality evidence showing significantly lower HTN with total and low-fat dairy, milk, and fermented dairy [58]. There was low-quality evidence, from 1 meta-analysis, for whole-fat dairy, yogurt, cheese, and fluid dairy products [58].

### Fruit and vegetables—RCTs

**FIGURE 6:**
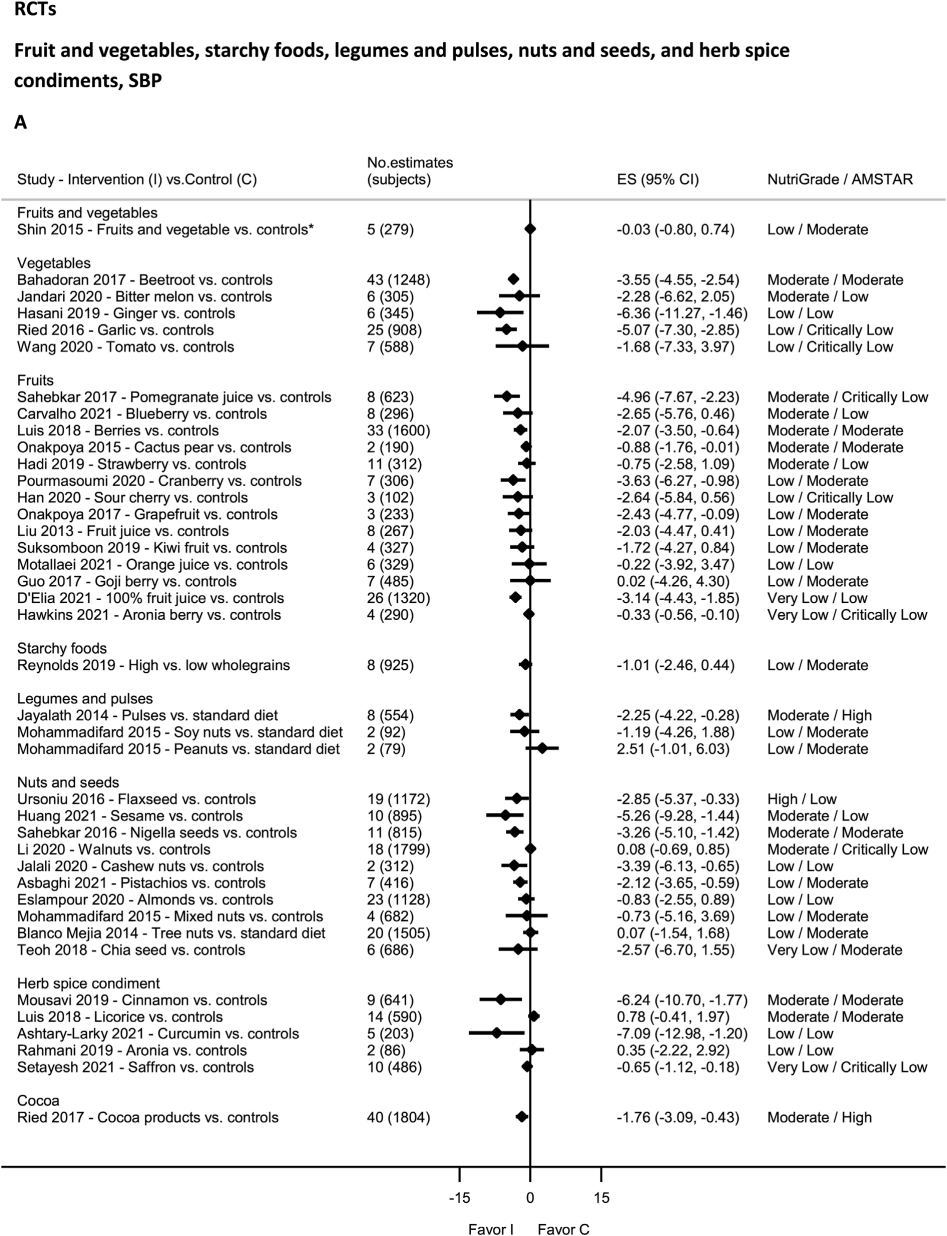

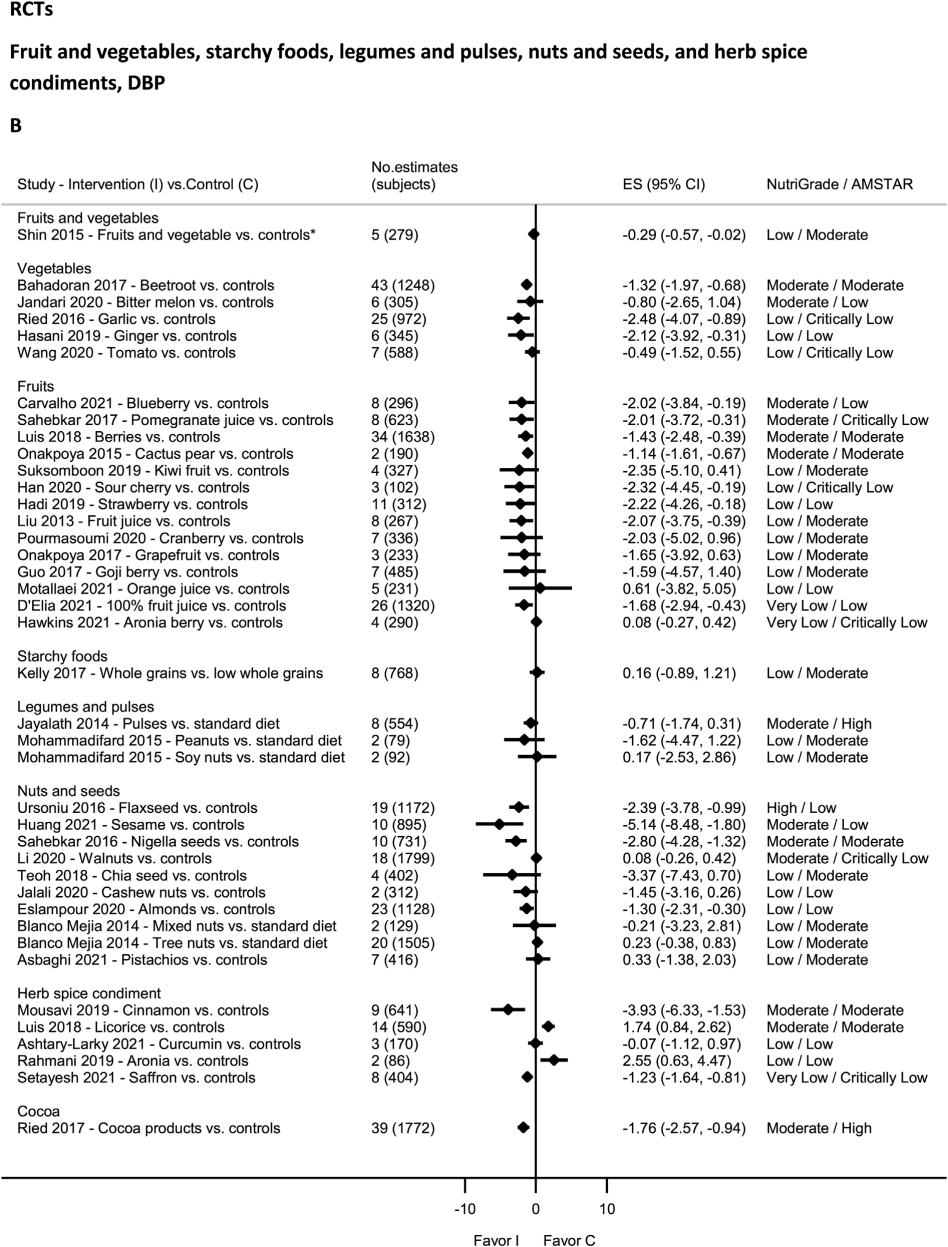
(A) Summary mean difference (ES), evidence quality (NutriGrade), and methodological quality (AMSTAR) of the meta-analyses of randomized controlled trials investigating the effects of fruit and vegetables, starchy foods, legumes and pulses, nuts and seeds, and herb spice condiments on systolic blood pressure. (B) Summary mean difference (ES), evidence quality (NutriGrade), and methodological quality (AMSTAR) of meta-analyses of randomized controlled trials investigating the effects of fruit and vegetables, starchy foods, legumes and pulses, nuts and seeds, and herb spice condiments on diastolic blood pressure. Each solid diamond and the horizontal line across the diamond represents the summary mean difference or summary standardized mean difference (∗) and its 95% confidence interval reported by the published meta-analysis. AMSTAR, Assessment of Multiple Systematic Reviews; C, control; ES, mean difference estimate; I, intervention.

No evidence was rated as high quality by NutriGrade (Figure 6A and B). There was moderate-quality evidence showing significantly decreased DBP with blueberry [122], SBP and DBP with beetroot [123], berries [124], cactus pear [45], and pomegranate [125] intakes. There was moderate-quality evidence showing nonsignificantly decreased SBP with blueberry [122], SBP and DBP with bitter melon [126] and strawberry (SBP) intakes [127]. Low-quality evidence was available for garlic [128], ginger [129], cranberry (SBP) [130], grapefruit (SBP) [131], strawberry (DBP) [127], sour cherry (DBP) [132], Goji berry [133], kiwi fruit [134], fruit juice [81], orange juice [135], fruits and vegetables [136], and tomato [137], and very-low-quality evidence for 100% fruit juice [138] and aronia berry [139].

### Fruit and vegetables—observational studies

No evidence was rated as high, moderate, or very low quality (Figure 3), rather only low-quality evidence was reported by NutriGrade, for fruits, vegetables, and fruits and vegetables [74].

### Starchy food: grains and potatoes—RCTs

No evidence was rated as high, moderate, or very low quality (Figure 6A and B), rather low-quality evidence by NutriGrade, for whole grains [140,141].

### Starchy food: grains and potatoes—observational studies

No evidence was rated as high, moderate, or low quality (Figure 3), rather only very-low-quality evidence rated by NutriGrade was reported for whole grains [18], refined grains [18], and from 1 meta-analysis for potatoes, baked/boiled mashed potatoes, and French fries [72].

### Legumes and pulses—RCTs

No evidence was rated as high or very low quality of evidence by NutriGrade (Figure 6A and B). There was moderate-quality evidence for intake of pulses (significant for SBP only) [142] and low quality of evidence for intakes of peanuts and soy nuts [43].

### Legumes and pulses—observational studies

No evidence was rated as high, moderate, or low quality by NutriGrade (Figure 3), rather only very-low-quality evidence for legumes [18].

### Nuts and seeds—RCTs

There was high-quality evidence by NutriGrade showing significantly decreased SBP and DBP with flaxseed intake (Figure 6A and B) [89] and moderate-quality evidence showing a significant reduction in SBP and DBP with intake of sesame [143], Nigella seeds [144], and a nonsignificant reduction in SBP and DBP with walnuts intake [145]. The evidence was rated as low quality for pistachio [146], mixed nuts (DBP) [37] (SBP) [43], tree nuts (DBP) [37], almonds [147], cashew nuts [41], chia seeds (DBP) [148]; and very low quality for chia seeds (SBP) [148] and tree nuts (SBP) [37].

### Nuts and seeds—observational studies

No evidence was rated as high, moderate or low quality by NutriGrade (Figure 3), rather only very low quality evidence for nuts [18].

### Cocoa—RCTs

No evidence was rated as high, low, or very low quality by NutriGrade (Figure 6A and B). There was moderate-quality evidence showing significantly decreased SBP and DBP with cocoa products intake [149].

### Cocoa—observational studies

No evidence was rated as high, moderate, or low quality by NutriGrade (Figure 3). There was only very-low-quality evidence for cocoa products [66].

### Herbs, spice, and condiment—RCTs

No evidence was rated as high quality by NutriGrade (Figure 6A and B). There was moderate-quality evidence showing significantly decreased SBP and DBP with intakes of cinnamon [82]; and increased DBP with licorice supplementation [150]. The evidence was rated as low quality for curcumin [83] and very low for saffron [151].

### Beverages—RCTs

**FIGURE 7:**
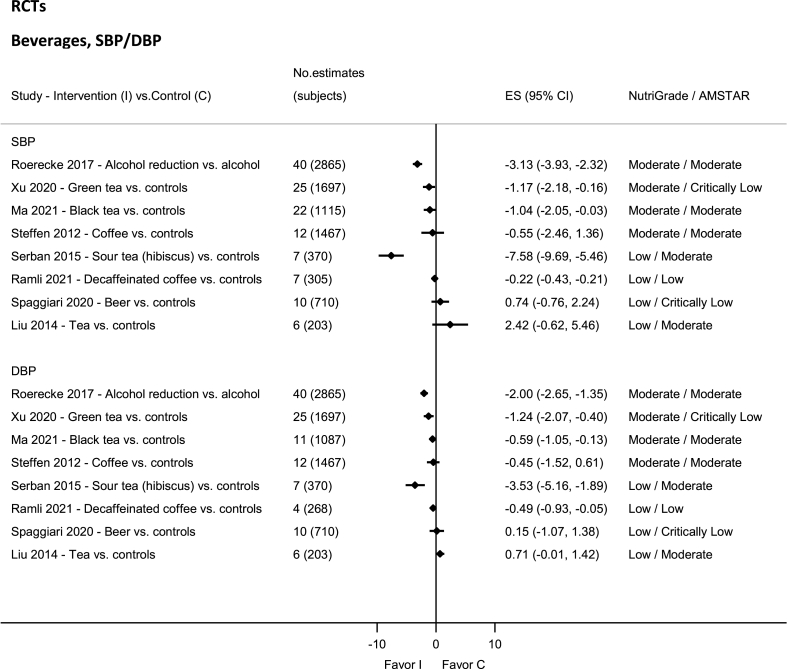
Summary mean difference (ES), evidence quality (NutriGrade), and methodological quality (AMSTAR) of the meta-analyses of randomized controlled trials investigating the effects of alcohol and other beverages on systolic and diastolic blood pressure. Each solid diamond and the horizontal line across the diamond represents the summary mean difference and its 95% confidence interval reported by the published meta-analysis. AMSTAR, Assessment of Multiple Systematic Reviews; C, control; ES, mean difference estimate; I, intervention.

No evidence was rated as high or very low quality by NutriGrade (Figure 7). There was moderate-quality evidence showing significant reductions in SBP and DBP with intakes of black tea [49], green tea [152], and alcohol [153]. There was moderate-quality evidence showing a nonsignificant reduction in SBP and DBP with coffee intake [154]. The evidence was rated as low quality for tea [48], decaffeined coffee [50], and hibiscus [155].

### Beverages—observational studies

**FIGURE 8:**
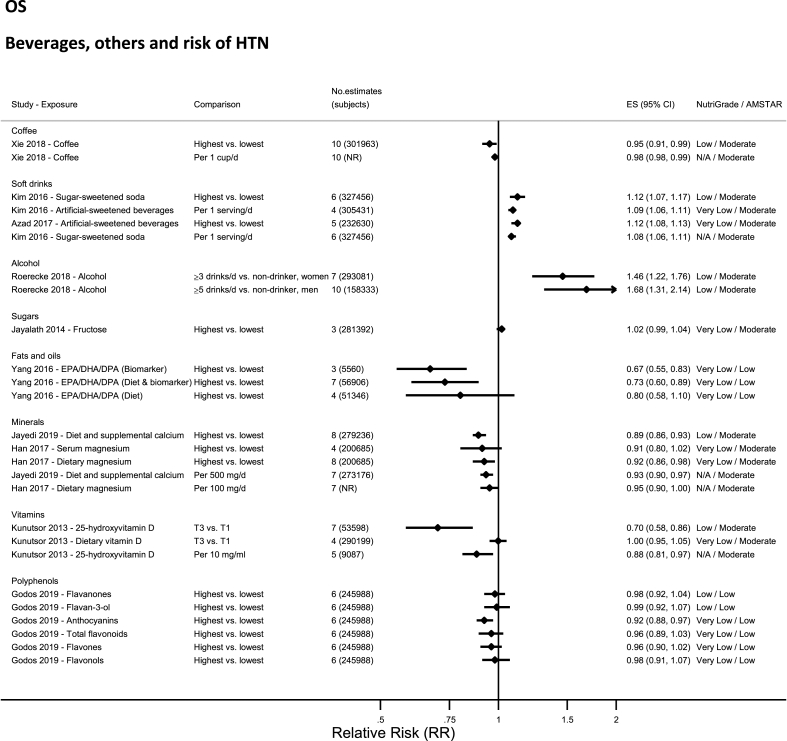
Summary relative risk (ES), evidence quality (NutriGrade), and methodological quality (AMSTAR) of the meta-analyses of observational studies investigating alcohol and other beverages in relation to risk of hypertension. Each solid diamond and the horizontal line across the diamond represents the summary relative risk and its 95% confidence interval for high vs. low comparison or per unit increment of exposure reported by the published meta-analysis. AMSTAR, Assessment of Multiple Systematic Reviews; DPA, docosapentaenoic acid; NR, not reported; T3 vs. T1, tertile 3 vs. tertile 1.

No evidence was rated as high or moderate quality by NutriGrade (Figure 8). There was low-quality evidence for alcohol [71], coffee [75], and soft drinks (sugar-sweetened) [61], and very-low-quality evidence for artificial-sweetened beverages [51].

### Carbohydrates, fiber, sugars—RCTs

**FIGURE 9:**
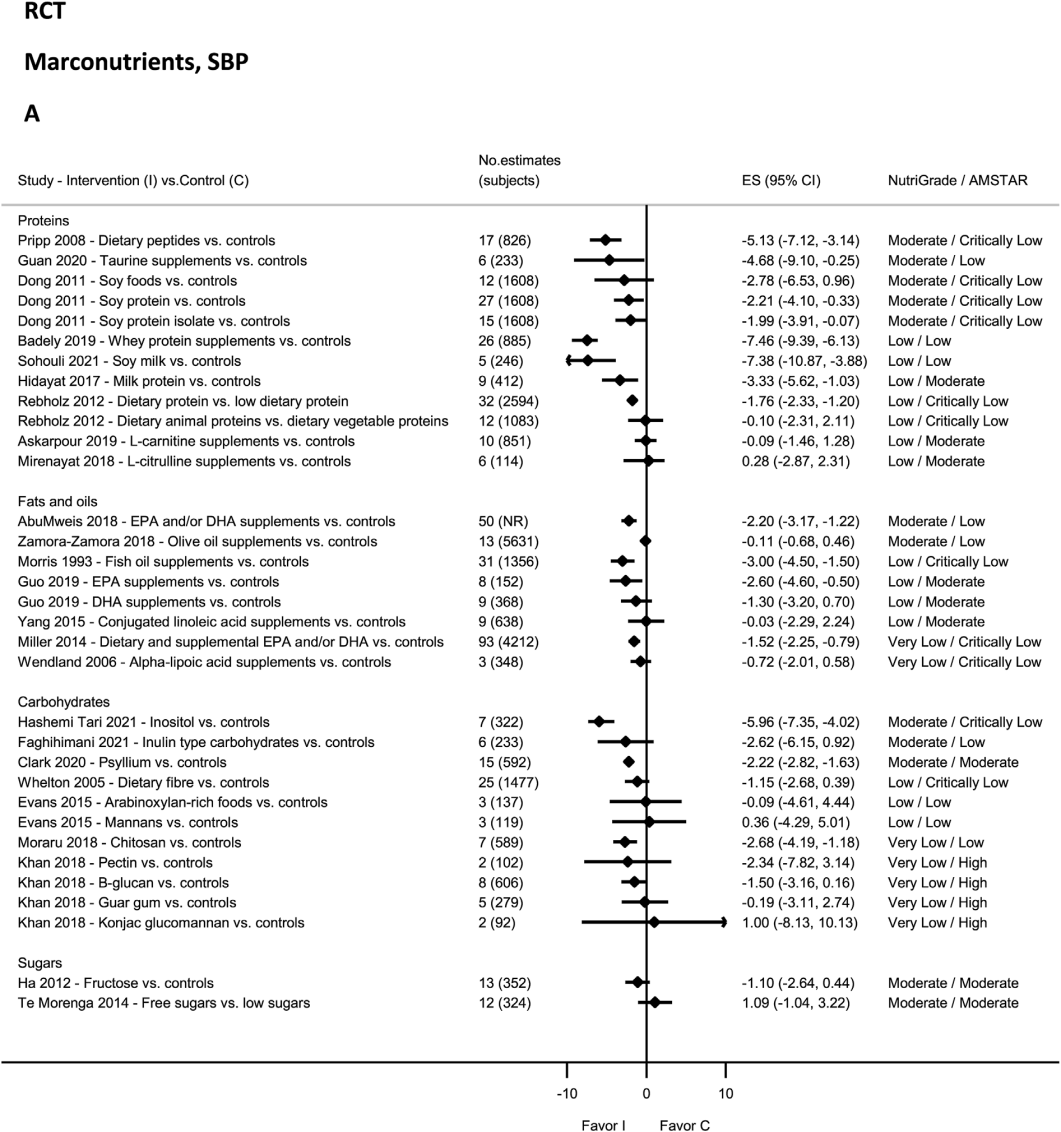

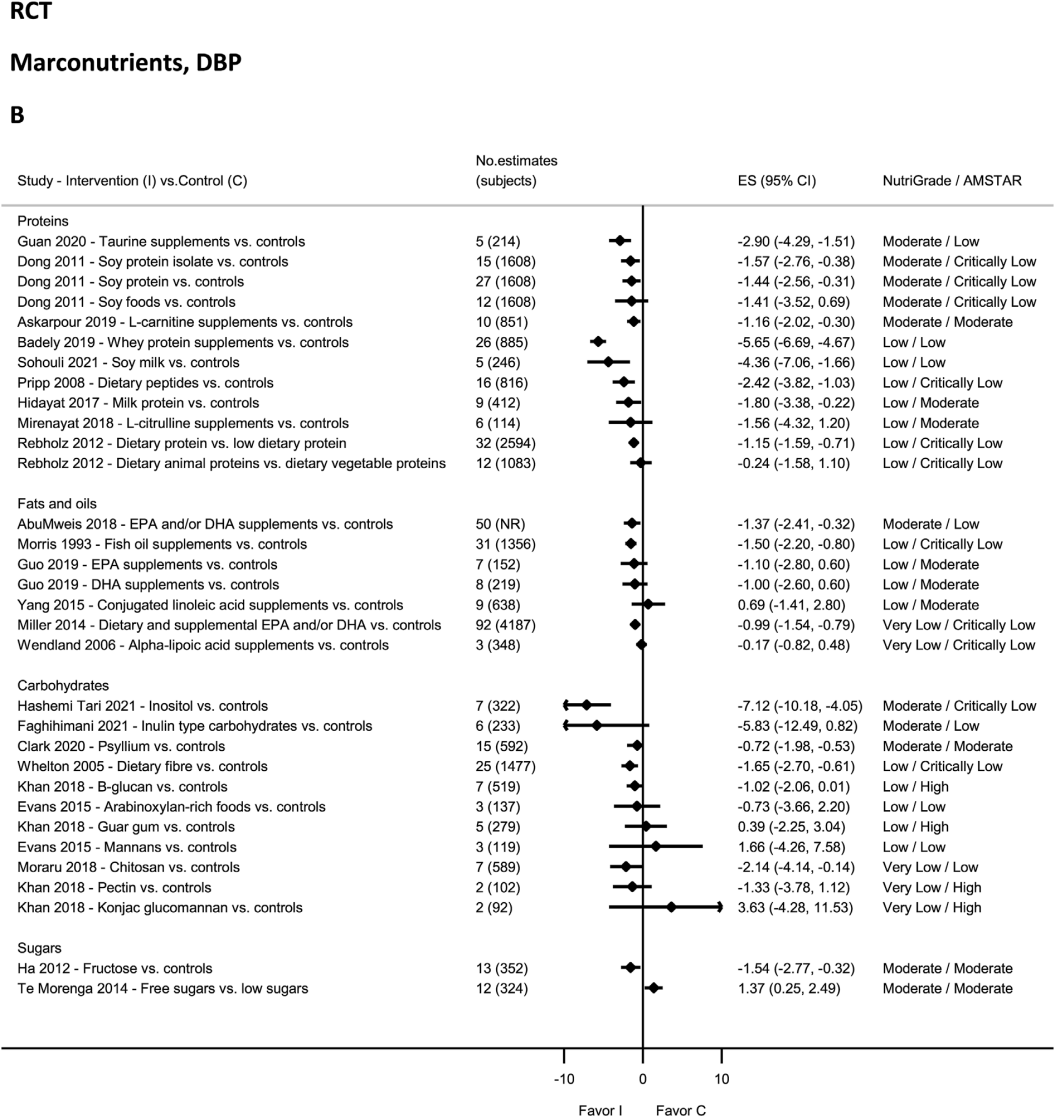
(A) Summary mean difference (ES), evidence quality (NutriGrade), and methodological quality (AMSTAR) of the meta-analyses of randomized controlled trials investigating the effects of macronutrients on systolic blood pressure. (B) Summary mean difference (ES), evidence quality (NutriGrade), and methodological quality (AMSTAR) of meta-analyses of randomized controlled trials investigating the effects of macronutrients on diastolic blood pressure. Each solid diamond and the horizontal line across the diamond represents the summary mean difference and its 95% confidence interval reported by the published meta-analysis. AMSTAR, Assessment of Multiple Systematic Reviews; C, control; ES, mean difference estimate; I, intervention; NR, not reported.

No evidence on carbohydrates was rated as high quality by NutriGrade (Figure 9A and B). There was moderate-quality evidence showing significant reductions in SBP and DBP with psyllium intake [156] and inositol [157]. There was moderate-quality evidence showing a nonsignificant reduction in SBP and DBP with inulin [158]. The evidence was rated as low quality for Arabinoxylan-rich foods and mannans [11], guar gum (DBP), and B-glucan (DBP) [42], and very-low-quality evidence for chitosan [159], and from the same meta-analysis for Konjac glucomannan, Pectin, B-glucan (SBP), and guar gum (SBP) [42]. None of the evidence on dietary fiber was rated as high, moderate, or very low quality by NutriGrade (Figure 9A and B), rather was low-quality evidence for total fiber [160]. None of the evidence on sugars was rated as high, low, or very low quality by NutriGrade (Figure 9A and B), rather there was moderate-quality evidence showing increased SBP and DBP with free-sugars intake [15]. Moderate-quality evidence revealed decreased DBP with fructose [161].

### Carbohydrates, fiber, and sugars—observational studies

No evidence was rated as high, moderate, or low quality by NutriGrade (Supplemental Table 4), only very-low-quality evidence for fructose [59].

### Proteins—RCTs

No evidence was rated as high or very low quality by NutriGrade (Figure 9A and B), but there was moderate-quality evidence showing significantly decreased SBP and DBP with intakes of L-carnitine supplements (DBP) [162], soy protein isolate [85], soy protein [85], Taurine supplement [84], and dietary peptides [163]. The evidence was rated as low quality for dietary protein and dietary animal and vegetable proteins [164], milk protein [165], soy milk [166], dietary peptides (DBP) [163], soy foods [85], whey protein supplements [167], L-carnitine supplements (SBP) [162], and L-citrulline supplementation [168].

### Proteins—observational studies

No evidence was rated as high, moderate, or low quality by NutriGrade (Supplemental Table 4), only very-low-quality evidence for dietary protein [65].

### Fats and oils—RCTs

No evidence was rated as high quality by NutriGrade (Figure 9A and B). There was moderate-quality evidence showing significantly decreased SBP and DBP with EPA and/or DHA supplements use [169]. There was moderate-quality evidence showing a nonsignificant reduction in SBP with olive oil supplements [170]. The evidence was rated as low quality for DHA and EPA supplements [171], fish oil supplements [172], and conjugated linoleic acid supplements [173], and very low quality for alpha-lipoic acid supplements [174], and dietary and supplemental EPA and/or DHA [175].

### Fats and oils—observational studies

No evidence was rated as high, moderate, or low quality by NutriGrade (Supplemental Table 4). There was very-low-quality evidence for EPA, DHA, and docosapentaenoic acid from dietary sources, biomarkers, and both dietary sources and biomarkers [76].

### Minerals—RCTs

**FIGURE 10:**
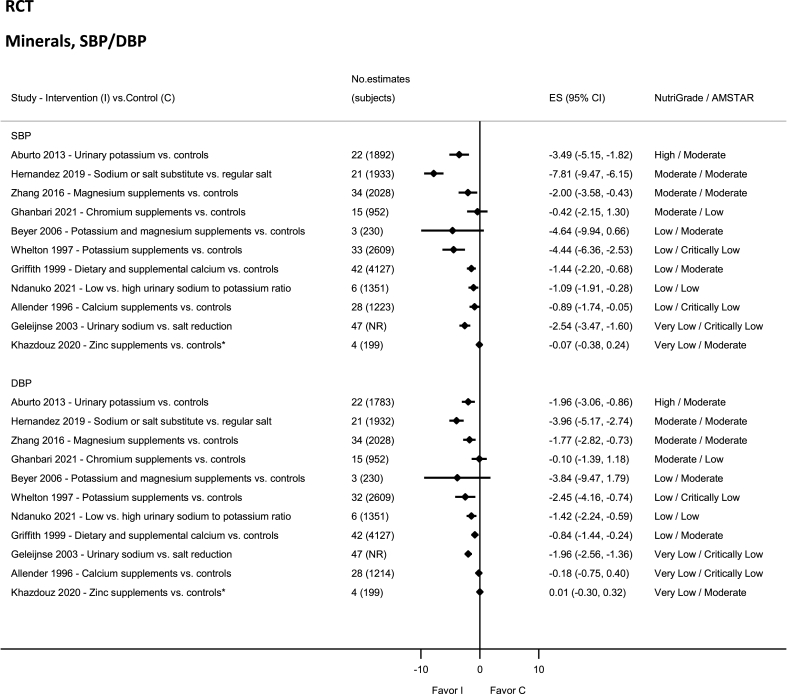
Summary mean difference (ES), evidence quality (NutriGrade), and methodological quality (AMSTAR) of the meta-analyses of randomized controlled trials investigating the effects of dietary minerals on systolic and diastolic blood pressure. Each solid diamond and the horizontal line across the diamond represents the summary mean difference or summary standardized mean difference (∗) and its 95% confidence interval reported by the published meta-analysis. AMSTAR, Assessment of Multiple Systematic Reviews; C, control; ES, mean difference estimate; I, intervention; NR, not reported.

There was high-quality evidence showing significant reductions in SBP and DBP with high urinary potassium levels (Figure 10) [94]. Moderate-quality evidence reported significantly reduced SBP and DBP with low sodium or salt substitute [19] and magnesium supplements [176]. Moderate-quality evidence showed nonsignificant reductions in SBP and DBP with chromium intake [177]. The evidence was rated as low quality for urinary sodium and potassium ratio [178], calcium (dietary and supplement) [179], calcium (SBP) (supplement) [86], potassium (supplement) [180], and potassium and magnesium (supplement) [181] in adults with hypertension. Very low quality of evidence was reported for calcium (DBP) (supplement) [86], zinc (supplement) [182], and reduced urinary sodium [183].

### Minerals—observational studies

No evidence was rated as high or moderate quality by NutriGrade (Figure 8). There was low-quality evidence showing significant inverse associations between dietary and supplemental calcium intake and risk of hypertension [60], and very-low-quality evidence for magnesium [57], selenium [63], and for urinary potassium [78].

### Vitamins—RCTs

**FIGURE 11:**
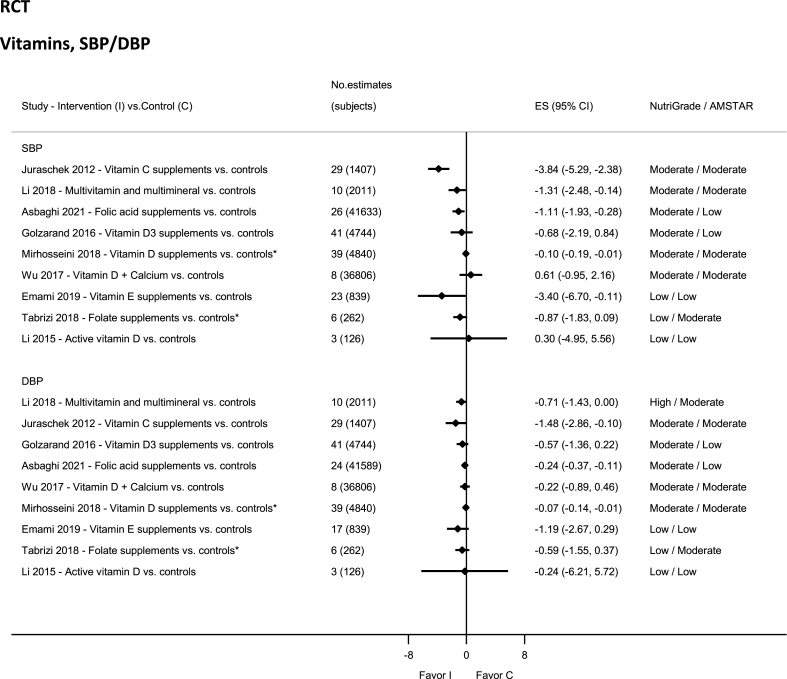
Summary mean difference (ES), evidence quality (NutriGrade), and methodological quality (AMSTAR) of the meta-analyses of randomized controlled trials investigating the effects of vitamin intakes on systolic and diastolic blood pressure. Each solid diamond and the horizontal line across the diamond represents the summary mean difference or summary standardized mean difference (∗) and its 95% confidence interval reported by the published meta-analysis. AMSTAR, Assessment of Multiple Systematic Reviews; C, control; ES, mean difference estimate; I, intervention.

There was high-quality evidence showing reduction in DBP with multivitamin intake (Figure 11) [90] and moderate-quality evidence showing significantly decreased SBP and DBP with intakes of vitamin C (supplements) [184], multivitamin intake (SBP) [90], and vitamin D (supplement) [185]. There was moderate-quality evidence showing nonsignificant results—decreased SBP and DBP with vitamin D3 supplement [186]; and decreased DBP but increased SBP with calcium plus vitamin D supplementation [47]. The evidence was rated as low quality for active vitamin D supplement [187], folate supplement [188], and vitamin E supplement [189].

### Vitamins—observational studies

No evidence was rated as high or moderate quality by NutriGrade (Figure 8). There was low-quality evidence for 25-hydroxyvitamin D level [62] and serum vitamin C [70], and very low for dietary vitamin D intake [62]

### Probiotics—RCTs

**FIGURE 12:**
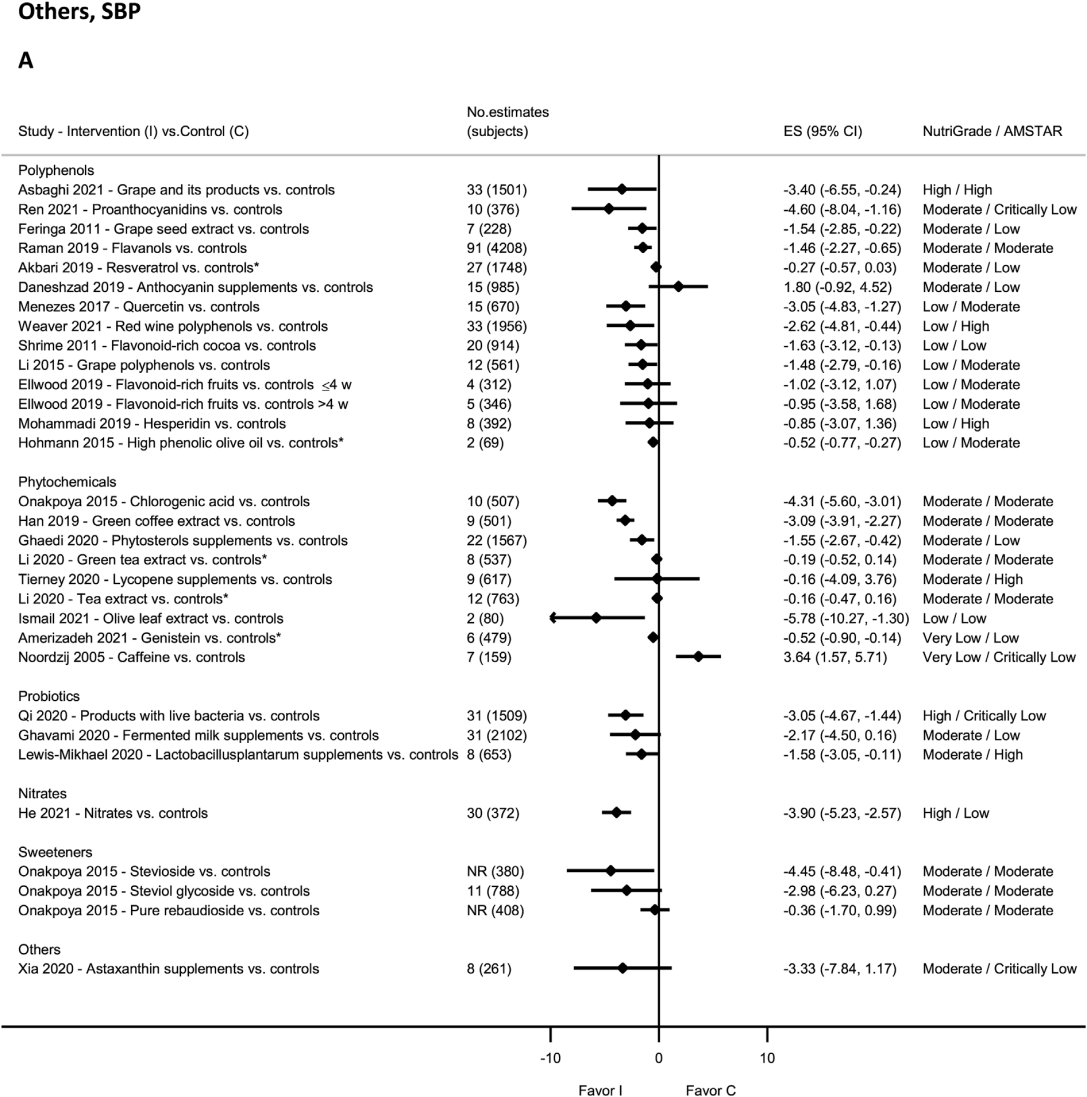

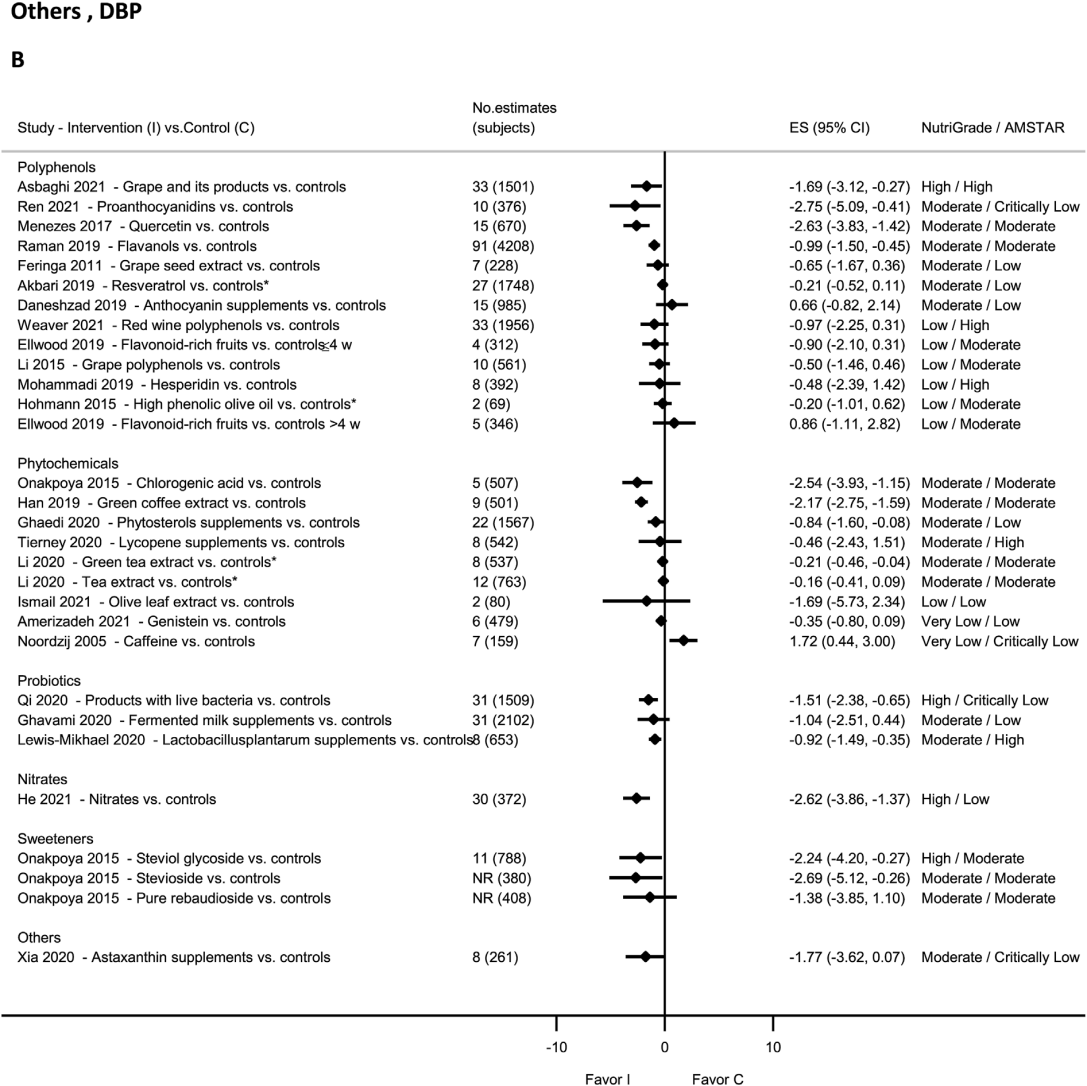
(A) Summary mean difference (ES), evidence quality (NutriGrade), and methodological quality (AMSTAR) of the meta-analyses of randomized controlled trials investigating the effects of polyphenols, phytochemicals, probiotics, nitrates, sweeteners, and other dietary factors on systolic blood pressure. (B) Summary mean difference (ES), evidence quality (NutriGrade), and methodological quality (AMSTAR) of the meta-analyses of randomized controlled trials investigating the effects of polyphenols, phytochemicals, probiotics, nitrates, sweeteners, and other dietary factors on diastolic blood pressure. Each solid diamond and the horizontal line across the diamond represent the summary mean difference or summary standardized mean difference (∗) and its 95% confidence interval reported by the published meta-analysis. AMSTAR, Assessment of Multiple Systematic Reviews; C, control; ES, mean difference estimate; I, intervention; NR, not reported.

There was high-quality evidence showing reduction in SBP and DBP with intake of live bacteria products by NutriGrade [91] (Figure 12A and B). There was moderate-quality evidence showing significantly decreased SBP and DBP with Lactobacillusplantarum supplements [190] and nonsignificant decrease in SBP and DBP with fermented milk [191].

### Polyphenols—RCTs

There was high-quality evidence showing reduction in SBP and DBP with grape intake and its products [92] (Figure 12A and B). There was moderate-quality evidence showing significant reductions in SBP and DBP with intakes of grape seed extract (SBP) [192], anthocyanins (berries, red grapes, and red wine) (SBP) [193], quercetin (DBP) [194], flavanols [46], and proanthocyanidins [87].

There was moderate-quality evidence showing a nonsignificant increase in SBP and DBP with anthocyanin supplements [195], and decrease in SBP and DBP with resveratrol supplementation [196] and grape seed extract (DBP) [192]. The evidence was rated as low-quality evidence for anthocyanins (berries, red grapes, and red wine) (DBP) [193], grape polyphenols [197], high-phenolic olive oil [39], hesperidin [198], hydrobenzoic acids [193], quercetin (SBP) [194], flavonoid-rich cocoa [199], red wine polyphenols [200], and flavonoid-rich fruits in adults with hypertension [201].

### Polyphenols—observational studies

No evidence was rated as high or moderate quality by NutriGrade (Supplemental Table 4), rather only low- and very-low-quality evidence for anthocyanins, flavanols, flavanones, flavones, flavonols, and total flavonoids [55].

### Phytochemicals—RCTs

No evidence was rated as high quality by NutriGrade (Figure 12A and B). There was moderate-quality evidence showing significantly decreased SBP and DBP with intakes of Phytosterols supplements [202], green coffee extract [203], green tea extract (DBP) [204], and chlorogenic acid [205]. There was moderate-quality evidence showing nonsignificant reductions in SBP and DBP with tea extract and green tea extract (SBP) [204], and Lycopene supplements [206]. The evidence was rated as low quality for olive leaf extract [40] and very low quality for caffeine [207] and Genistein [208].

### Nitrites and nitrates—RCTs

There was high-quality evidence on NutriGrade showing significant reductions in SBP with nitrates (Figure 12A and B) and moderate-quality evidence showing significant reductions in DBP [93].

### Sweeteners—RCTs

There was high-quality evidence on NutriGrade showing significantly decreased DBP with steviol glycoside intake [95] and moderate-quality evidence showing significant reductions in SBP and DBP with stevioside intake [95] (Figure 12A and B). The evidence was rated as moderate quality but showed nonsignificant reductions in SBP and DBP with intakes of pure rebaudioside and steviol glycoside (SBP) [95].

### Others—RCTs

There was moderate-quality evidence on NutriGrade showing a nonsignificant decrease in SBP and DBP with Astaxanthin supplement [209] (Figure 12A and B).

## Discussion

### Principle findings

This umbrella review of meta-analyses of RCTs and observational studies provides the first extensive and comprehensive overview that synthesizes and grades the strength of evidence of numerous dietary factors and changes in BP and risk of hypertension. We reviewed a total of 175 publications and reported 341 meta-analyses of RCTs and 70 meta-analyses of observational studies, using NutriGrade to assess the quality of each selected meta-analysis and AMSTAR 2 for a methodological quality of included publications. Meta-analyses of DASH dietary patterns showed that it was the most effective dietary pattern for reducing BP and its effect was comparable to antihypertensive pharmacological treatment, with similar findings for the Mediterranean diet. As part of our analyses, we found that the majority of studies investigating the relationship between dietary components and hypertension and BP were of poor-quality evidence or poorly reported. Despite this, the examined evidence supports the dietary guidelines recommended by several authoritative health bodies and highlights the relationship between BP and several other dietary factors.

Meta-analyses of RCTs for DASH, very-low-carbohydrate ketogenic diet (DBP), flaxseed, nitrates (SBP), urinary potassium, multivitamins and multiminerals (DBP), steviol (DBP), products with live bacteria, nitrates (SBP), and grape and its products were graded as high confidence in the effect estimate using NutriGrade, and further research probably will not change the confidence in the effect estimate. This review shows that no meta-analyses of observational studies were graded as high, and very few were graded as moderate quality. Further research could add evidence on the confidence and may change the effect estimate of meta-analyses having low or very low quality. Likewise, the methodological quality assessed using AMSTAR 2 was graded as high in only 11 of the included publications (all RCTs), with a majority of publications graded as moderate (43% for RCTs publications and 66% for observation studies’ publications). The poor-quality assessments may be due to the lack of quantitative risk of bias assessment, selection bias, significant heterogeneity, a small number of primary studies (<5), BP considered as a surrogate marker, absence of a test for publication bias, unreported source of funding, or moderate effect sizes (for observational studies), while the poor reporting may be due to the absence of grey literature or list of excluded studies. Overall, our review found that risk of bias remains uncertain for most of the available trials owing to poor reporting. This point is particularly concerning given that the majority of the trials were conducted after the Consolidated Standards of Reporting Trials guidelines were first reported in 1993 and published in 1996 [210]. We also found high heterogeneity, which may be due to the differences in trial designs, comparison groups, study populations, and analysis methods.

This umbrella review supports current recommended guidelines for the management and prevention of hypertension [1,9] by adhering to the DASH and Mediterranean dietary patterns, and restricting sodium with moderate alcohol intake. In accordance with these recommendations, we reported that the DASH dietary pattern (high-quality RCTs) significantly lowers SBP and DBP [96], as do some of its components, including fruits and vegetables [45,74,123,124], whole grains [18], legumes and pulses [18,142], nuts and seeds [18,89,144,211], total red meat and poultry [77], and lactotripeptides intake [120]. We also found urinary potassium excretion (high-quality RCTs) [94], low-sodium diets (moderate-quality RCTs) [96] associated with lower BP, but not with low-fat dairy (not significant for RCTs, but significant in moderate-quality observational studies) [121]. Our report supports recommendations for adhering to the Mediterranean diet, on the basis of moderate-quality evidence from RCTs [100] and very-low-quality evidence from observational studies. The lowering effects of alcohol reduction were also evident on the basis of moderate-quality evidence from RCTs [153] and low-quality evidence from observational studies. However, our review did not indicate a beneficial effect of decreased BP with moderate-quality RCTs of increased vegan diet [106], hyperproteic diet [107], low-carb, high-fat diet or low-carb, high-protein diet [44], and fish [117].

### Possible explanations

Biological mechanisms can be used to explain many of the findings from this study. For instance, the DASH diet and its components can reduce BP, as shown with high-quality evidence, by improving peripheral vascular function and associated metabolic improvements [212,213]. Moderate-quality evidence of restricted sodium diets can reduce BP through elevated plasma renin activity, serum aldosterone, plasma levels of noradrenaline and adrenaline, total cholesterol, and triglycerides [214]. The effect of low-calorie or fat diets on BP can be attributed to improvement in insulin resistance [215]. Furthermore, high-potassium intake decreased blood pressure in moderate-quality studies. This agrees with animal models that reported increased renin–angiotensin activity, renal injury, and raised BP in potassium-depleted rats [216,217]. For a high-protein diet and a very-low-carbohydrate ketogenic diet, the BP-lowering effect may be explained by the amino acid content, specifically arginine, a substrate for nitric oxide, that improves vasodilation, endothelial function, and insulin resistance leading to lower BP [218,219], or by the partially substituting carbohydrate with protein intake [220]. Nitric oxide may also play a role in the BP-lowering effects of other dietary components. For example, the BP-lowering effects of soy protein reported in moderate-quality evidence may be attributed to the actions of estrogen [221] and other constituents of soya, such as arginine, a precursor of nitric oxide [222]. Likewise, L-carnitine supplements demonstrated reduced DBP in moderate-quality studies, mainly due to an increase in nitric oxide [223]. Vitamin C was also found to reduce BP in moderate-quality studies, mainly related to increases in the tetrahydrobiopterin level, a cofactor that enhances the production of nitric oxide and bioavailability [224]. As for nitrates and nitrate-rich sources such as beetroot, the BP-lowering effects reported from moderate-/high-quality evidence may be related to nitric oxide production maintaining vascular health [225]. Furthermore, bioactive polyphenols and their constituents such as flavonoids, present in beetroot [226], berries, cactus pear, pomegranate, and cocoa, may be responsible for the BP reduction effects reported in moderate-quality studies through promoting nitric oxide bioactivity, reduction of some inflammatory pathways [227], and reduction of vascular resistance and arterial stiffness [228,229], which may lead to improved vascular function. Curcumin was associated with decreased BP on the basis of moderate-quality evidence, mainly through the reduction of oxidative stress and the induction of anti-inflammatory effects by suppressing factors such as adhesion molecules and nuclear factor-kappa [230], in addition to increasing nitric oxide bioavailability and inhibition of angiotensin-converting enzymes [231]. Similar explanations were suggested for the BP-lowering effects of quercetin and green tea as reported by moderate-quality evidence; in addition, green tea was shown to maintain vascular tone by balancing vasoconstricting substances, such as angiotensin II, prostaglandins, and endothelin-1 [194].

Furthermore, the BP-lowering effect by flaxseed seen in high-quality studies may be attributed to its components, such as phytoestrogens that reduced angiotensin I-induced increase of BP and alpha-linolenic acid, which reduces BP [232] through lowering the activity of soluble epoxide hydrolase [233]. EPA and/or DHA supplements reduced BP according to moderate-quality evidence. This may be related to the stimulated synthesis of prostacyclin by n-3 PUFA [234], which acts as a vasodilator, and reduces vascular resistance with n-3 PUFA [235]. Magnesium supplements showed reductions in BP as reported by moderate-quality evidence. It has been proposed that magnesium functions as a calcium channel blocker, stimulating endothelial function and vasodilation [236]. Vitamin D supplements were also found to decrease BP, as reported in moderate-quality evidence. The mechanism may be related to the association of vitamin D with reducing oxidative stress and inflammatory responses, improving endothelial function, and glucose homeostasis, and other cardioprotective effects [237]. Findings of reduced BP with green coffee extract and chlorogenic acid from moderate-quality evidence may be due to the chlorogenic acids present in green coffee that reduces the stress hormone cortisol [238], regulates glucose metabolism, and suppresses macrophage infiltration leading to blood vessel remodeling [239]. The beneficial effects of lactotripeptides and live bacteria reported in moderate-quality studies for decreasing the BP level include improved lipid levels, insulin resistance, and regulation of the renin–angiotensin system [240]. In addition, cocoa contains theobromine, linked to vasoactivity and consequently blood pressure reduction [241].

Findings of reduced BP in moderate-quality evidence with fructose intakes are inconsistent with clinical and animal studies. This may be explained by the heterogeneity in BP measures in the included studies, such as whether postprandial BP, which is most affected by fructose, was measured, rather than after an overnight fast when fructose may have been completely metabolized [161]. On the contrary, free sugars increased BP according to moderate-quality evidence, likely due to the fructose content that has been shown to stimulate triglycerides and cholesterol circulation, elevated synthesis of urate, which decreases nitric oxide synthesis resulting in vasoconstriction [242]. Licorice, used in producing candies and sweets, elevated BP according to moderate-quality evidence through its electrolyte content and effects on the renin–angiotensin–aldosterone axis [243]. Lastly, steviol glycosides were associated with decreased SBP [95], which can be explained by increased renal plasma flow and glomerular filtration rate [244].

### Comparison with previous umbrella reviews

Findings from previous umbrella reviews were comparable with some of the findings presented here. For example, Dinu et al. [21] reported that greater adherence to the Mediterranean diet reduced BP in their meta-analyses of RCTs, whereas no evidence was presented for observational studies. We found moderate-quality evidence for reduced BP with the Mediterranean diet, and no evidence of an association in observational studies. Other umbrella reviews reported BP-lowering effects of high-protein diets [22], low-fat diets [22], and DASH [22,245], Nordic [245], and portfolio dietary patterns [245], consistent with our findings. Other umbrella reviews were also consistent with our report; for instance, Grosso et al. [26] reported no effects of BP with coffee intake whereas caffeine intake significantly increased BP; Schwingshackl et al. [27] and Wan et al. [30] found significant reductions in BP with garlic intake; Veronese et al. [28] reported no effects of chocolate on BP; Theodoratou et al. [24] reported no effects of supplemental vitamin D on BP; Godos et al. [32] reported lower hypertension risk and high BP with total dairy consumption; another study found the reducing effects of dietary fiber on DBP only [25]; and no effects for nuts on BP were reported in previous umbrella reviews [31]. Contrary to our findings, Ashor et al. [23] reported no effects of vitamin C on BP, which may be attributed to characteristics of included trials and large heterogeneity.

### RCTs compared with observational studies

Intervention studies have known design advantages over observational studies ,including objective dietary measures, randomization including a control group. Observational studies are constrained by the scope of data relating to diet and other confounding variables, often subject to self-measurement error, residual confounding, and recall bias [246]. Despite the superiority of RCTs from a design perspective, the applicability of findings to the real world is challenging (particularly inadequate compliance in long-term dietary intervention trails) and may be limited by physiologically unacceptable levels of intake, study population, and outcomes measured [247]. Dietary recommendations and policies should be guided by rigorous systematic reviews and different study designs addressing the same dietary exposure could provide different results; any review of poor methodological quality could be misleading [248]. Therefore, the integration of evidence from both observational studies and intervention studies are required to improve dietary quality and lower disease burden.

### Strengths and limitations

Our umbrella review has several strengths. We provide the first systematic, comprehensive overview of the role of dietary factors and risk of hypertension and change in BP. Furthermore, we evaluated the methodological quality and quantity of evidence using validated tools: AMSTAR 2 and NutriGrade, the latter is designed on the basis of widely used Grade but tailored for nutritional studies. This approach allowed critical identification of good-quality evidence from well-designed meta-analyses that showed statistical and more importantly clinically significant effects/associations that inform public health recommendations and policy formulation. We also highlighted topics with low-quality reviews to potentially stimulate further research efforts. Several dietary factors evaluated by RCTs are less prone to the biases inherent in observational studies, which are, for nutrition-disease research, a rare quality.

Our umbrella review also had several limitations. For example, published meta-analyses of the same dietary factor might only have few common studies because of the different inclusion criteria for the review. Interventions varied substantially (active ingredients, administrative forms, dosing, and duration) between the included RCTs, which may explain the observed heterogeneity. Furthermore, the lack of intervention blinding, a challenging aspect of nutrition-related studies, may increase risk of bias and decrease the accuracy of results. Included observational studies were of varying design, and used different analytical methods and diverse study populations, which may explain the observed heterogeneity. In addition, BP measurement methods varied across publications and may be limited by random or systematic errors [249]. Another limitation is that the use of office BP does not capture measurements during longer periods of time or in everyday activities [249].

Furthermore, evidence on other subgroups, for example, sex, age, geographical locations, race, ethnicity, diabetes or chronic kidney disease status, and weight category were not synthesized in the current umbrella review. These factors could bias the observed associations. The current umbrella review is dependent on the reporting of the published meta-analyses and could not account for any potential missing primary studies, overlapping of primary studies or study participants. Finally, we repeated the search for the time lapsed from our initial search and included publications from 1 December 2019 to 31 October 2021. We identified 3578 relevant publications. A total of 166 publications were identified as exposures covered in the current review, these were compared for the direction and magnitude of effect and where appropriate, we replaced publications from our previous search.

## Conclusions and recommendations

The effect and association of dietary patterns, food groups, single foods, beverages, macronutrients, and micronutrients with risk of hypertension and change in BP were previously examined in several published meta-analyses, but this umbrella review categorically supports recommended dietary guidelines involving the DASH and Mediterranean patterns, restricting sodium, with moderate alcohol intake, as indicated by mostly moderate-quality RCTs. To achieve high-quality evidence and provide strong recommendations, future studies should consider several topics with only low quality of evidence observed (that is, small number of studies, reported bias, etc.). Future studies should focus on exposures likely to be biologically associated with risk of hypertension and change in BP but currently showing that the quality of evidence is still low. Future research should also focus on new dietary factors that have not yet been investigated (or published) so far. It is worth noting that this paper primarily focuses on dietary variables and comparisons across studies, rather than the actual differences between BP levels on the basis of baseline variability. Nonetheless, the majority of the studies examined the intervention effect on the basis of the difference of changes of BP from baseline. Hence, Wilder’s principle [250]—the expected difference in BP from the initial reading is higher if the initial BP reading is high—may be at play. In this regard, future studies should explore the potential influence of Wilder’s principle by observing the actual differences between BP levels on the basis of baseline variability.

## Author contributions

The authors’ responsibilities were as follows—RG, GSA, DSMC, QC: designed research, conducted research, analyzed data, and performed analysis; RG, GSA, QC: wrote the paper; DSMC, LVH: revised the work critically for important intellectual content; QC: had primary responsibility for final content; and all authors: read and approved the final manuscript.

## Funding

The authors received no financial support for the research, authorship, and/or publication of this article.

## Conflict of interest

None of the authors report a conflict of interest related to research presented in this article. QC is an employee and shareholder of Amgen Inc. The work presented here was conducted while QC was an employee of Imperial College London. Amgen Inc. was not involved in this study.
