# Supplementary material for: The Role of Diet in the Prevention of Hypertension and Management of Blood Pressure: An Umbrella Review of Meta-Analyses of Interventional and Observational Studies
Source: Adv Nutr. 2023 Oct 1;15(1):100123. doi: 10.1016/j.advnut.2023.09.011 (PMC10831905; doi:10.1016/j.advnut.2023.09.011)
Supplement: Multimedia component 1 [file mmc1.docx]

**Role of diet and blood pressure and risk of hypertension: An umbrella review of meta-analyses of interventional and observational studies**

**Author list:** Ghadeer S. Aljuraiban, Rachel Gibson, Doris SM Chan, Linda Van Horn, Queenie Chan

**Supplementary Table 1 Search terms**

**Combined steps:**

((diet[mesh] OR diet[tiab] OR diets[tiab] OR dietetics[mesh] OR dietetic[tiab] OR dietary[tiab] OR eating[mesh] OR eating[tiab] OR intake[tiab]) OR (nutrition[mesh] OR nutrition[tiab] OR nutrients[mesh] OR nutrient[tiab] OR nutrients[tiab]) OR (food and beverages[mesh] OR food*[tiab] OR food group*[tiab] OR food cluster*[tiab] OR food pattern*[tiab] OR dietary pattern*[tiab] OR diet therapy[mesh] OR diet quality[tiab]) OR (meal*[tiab] OR eating pattern*[tiab] OR fasting[tiab]) OR (vegetarian*[tiab] OR vegan*[tiab]) OR (bread[tiab] OR cereal*[tiab] OR grain*[tiab] OR corn[tiab] OR wholegrain[tiab] OR whole grain[tiab] OR wholewheat[tiab] OR whole wheat[tiab] OR granary[tiab] OR soy[tiab] OR soya[tiab] OR potato*[tiab] OR tuber[tiab] OR tubers[tiab] OR roots[tiab] OR pulses[tiab] OR legume*[tiab] OR lentils[tiab] OR beans[tiab] OR chickpeas[tiab] OR rice[tiab] OR pasta[tiab] OR quinoa[tiab]) OR (vegetables[mesh] OR vegetable*[tiab] OR fruit[mesh] OR fruit*[tiab]) OR (milk[tiab] OR dairy[tiab] OR dairy product*[tiab] OR yoghurt[tiab] OR cheese[tiab]) OR (meat[mesh] OR meat[tiab] OR pork[tiab] OR lamb[tiab] OR beef[tiab] OR poultry[tiab] OR chicken[tiab] OR turkey[tiab] OR duck[tiab] OR fish[tiab] OR seafood[tiab] OR shellfish[tiab] OR egg[tiab] OR eggs[tiab]) OR (oil*[tiab] OR butter[tiab] OR margarine[tiab]) OR (nuts[mesh] OR nut[tiab] OR nuts[tiab] OR peanut*[tiab] OR groundnut*[tiab] OR seeds[tiab]) OR (desert[tiab] OR sweets[tiab] OR sweetened[tiab] OR candy[tiab] OR sugar[tiab] OR syrup[tiab]) OR (beverage*[tiab] OR drinks[tiab] OR drinking[tiab]) OR (alcohol[tiab] OR alcoholic[tiab] OR ethanol[tiab] OR beer[tiab] OR wine[tiab] OR spirits[tiab] OR liquor[tiab]) OR (caffeine[tiab] OR coffee[tiab] OR tea[tiab]) OR (fruit and vegetable juices[mesh] OR juice[tiab] OR lemonade[tiab] OR sugar sweetened beverage*[tiab]) OR (food preservation[mesh] OR pickled[tiab] OR bottled[tiab] OR canned[tiab] OR cured[tiab] OR smoked[tiab] OR preserved[tiab] OR processed[tiab] OR sweetening agents[mesh] OR sweetener*[tiab] OR flavouring*[tiab] OR flavoring*[tiab]) OR (macronutrient*[tiab] OR micronutrient*[tiab]) OR (lipids[mesh] OR fat[tiab] OR fats[tiab] OR fatty[tiab] OR fatty acids[tiab]) OR (dietary carbohydrates[mesh] OR carbohydrate*[tiab] OR fibre[tiab] OR fiber[tiab] OR polysaccharide*[tiab] OR starch[tiab] OR starchy[tiab] OR sugar*[tiab] OR sucrose[tiab] OR fructose[tiab]) OR (dietary proteins[mesh] OR amino acid*[tiab]) OR cholesterol[tiab] OR (allium[mesh] OR onion[tiab] OR garlic[tiab]) OR (vitamins[mesh] OR vitamin*[tiab] OR mineral*[tiab] OR dietary supplements[mesh] OR nitrates[tiab] OR nitrites[tiab]) OR (polyphenols[mesh] OR polyphenol*[tiab] OR antioxidant*[tiab]) OR (sodium[mesh] OR sodium[tiab] OR salt[tiab] OR salting[tiab] OR salted[tiab]) OR (energy intake[tiab] OR energy density[tiab]))

AND

((hypertension[mesh] OR hypertension[tiab] OR prehypertensive[tiab] OR pre-hypertensive[tiab] OR prehypertension[tiab] OR pre-hypertension[tiab] OR "Hypertension, Pregnancy-Induced"[mesh] OR pregnancy induced hypertension[tiab] OR pregnancy-induced hypertension[tiab] OR pregnancy transient hypertension[tiab] OR pregnancy-transient hypertension[tiab] OR pregnancy related hypertension[tiab] OR pregnancy-related hypertension[tiab] OR gestational hypertension[tiab]) OR (metabolic syndrome[mesh] OR metabolic syndrome[tiab]) OR (blood pressure[mesh] OR systolic blood pressure[tiab] OR systolic pressure[tiab] OR diastolic blood pressure[tiab] OR diastolic pressure[tiab]) OR ("Cardiovascular Diseases/epidemiology"[mesh] OR "Cardiovascular Diseases/etiology"[mesh] OR "Cardiovascular Diseases/prevention and control"[mesh]) OR ("Pregnancy Complications, Cardiovascular/epidemiology"[mesh] OR "Pregnancy Complications, Cardiovascular/etiology"[mesh] OR "Pregnancy Complications, Cardiovascular/prevention and control"[mesh]))

NOT

(animal[mesh] NOT human[mesh])

AND

("systematic review" OR “systematic literature review” OR meta-analysis OR metaanalysis)

Return 6,234 records up to 31^st^ Oct 2021

Optional:

If filter #37 by article types – meta-analysis OR systematic reviews instead:

#37 AND (Meta-Analysis[ptyp] OR systematic[sb]))

**Supplementary Table 2 List of superseded studies, n=166**

| **Ref#** | **Title** | **Diet exposure** | **Reason for replacement** |
| --- | --- | --- | --- |
| 1 | Akilen R, Pimlott Z, Tsiami A, Robinson N. Effect of short-term administration of cinnamon on blood pressure in patients with prediabetes and type 2 diabetes. Nutrition. 2013;29(10):1192-6. | Herb, spice, and condiment | populatoin: prediabetes, T2DM |
| 2 | Alam S, Johnson AG. A meta-analysis of randomised controlled trials (RCT) among healthy normotensive and essential hypertensive elderly patients to determine the effect of high salt (NaCl) diet of blood pressure. J Hum Hypertens. 1999;13(6):367-74. | Minerals | fewer studies than chosen one |
| 3 | Allender PS, Cutler JA, Follmann D, Cappuccio FP, Pryer J, Elliott P. Dietary calcium and blood pressure: a meta-analysis of randomized clinical trials. Ann Intern Med. 1996;124(9):825-31. | Minerals | fewer studies than chosen one |
| 4 | Anker CCB, Rafiq S, Jeppesen PB. Effect of steviol glycosides on human health with emphasis on type 2 diabetic biomarkers: A systematic review and meta-analysis of randomized controlled trials. Nutrients. 2019;11(9):1965. | Sweeteners | fewer studies than chosen one |
| 5 | Appel LJ, Miller ER, 3rd, Seidler AJ, Whelton PK. Does supplementation of diet with 'fish oil' reduce blood pressure? A meta-analysis of controlled clinical trials. Arch Intern Med. 1993;153(12):1429-38. | Fats and oils | fewer studies than chosen one |
| 6 | Asgary S, Karimi R, Momtaz S, Naseri R, Farzaei MH. Effect of resveratrol on metabolic syndrome components: A systematic review and meta-analysis. Rev Endocr Metab Disord. 2019;20(2):173-86. | Polyphenols | fewer studies than chosen one |
| 7 | Ashor AW, Lara J, Siervo M. Medium-term effects of dietary nitrate supplementation on systolic and diastolic blood pressure in adults: a systematic review and meta-analysis. J Hypertens. 2017;35(7):1353-9. | Nitrates | fewer studies than chosen one |
| 8 | Askarpour M, Ghaedi E, Roshanravan N, Hadi A, Mohammadi H, Symonds ME, et al. Policosanol supplementation significantly improves blood pressure among adults: A systematic review and meta-analysis of randomized controlled trials. Complement Ther Med. 2019;45:89-97. | Carbohydrates | population: dyslipidemia |
| 9 | Binia A, Jaeger J, Hu Y, Singh A, Zimmermann D. Daily potassium intake and sodium-to-potassium ratio in the reduction of blood pressure: a meta-analysis of randomized controlled trials. J Hypertens. 2015;33(8):1509-20. | Minerals | fewer studies than chosen one |
| 10 | Brunner E, White I, Thorogood M, Bristow A, Curle D, Marmot M. Can dietary interventions change diet and cardiovascular risk factors? A meta-analysis of randomized controlled trials. Am J Public Health. 1997;87(9):1415-22. | Patterns of diet | fewer studies than chosen one |
| 11 | Cappuccio FP, Elliott P, Allender PS, Pryer J, Follman DA, Cutler JA. Epidemiologic association between dietary calcium intake and blood pressure: a meta-analysis of published data. Am J Epidemiol. 1995;142(9):935-45. | Minerals | fewer studies than chosen one |
| 12 | Cappuccio FP, MacGregor GA. Does potassium supplementation lower blood pressure? A meta-analysis of published trials. J Hypertens. 1991;9(5):465-73. | Minerals | fewer studies than chosen one |
| 13 | Castellana M, Conte E, Cignarelli A, Perrini S, Giustina A, Giovanella L, et al. Efficacy and safety of very low calorie ketogenic diet (VLCKD) in patients with overweight and obesity: A systematic review and meta-analysis. Rev Endocr Metab Disord. 2019. | Patterns of diet | population: obese or overweight only |
| 14 | Chanson-Rolle A, Aubin F, Braesco V, Hamasaki T, Kitakaze M. Influence of the Lactotripeptides Isoleucine-Proline-Proline and Valine-Proline-Proline on Systolic Blood Pressure in Japanese Subjects: A Systematic Review and Meta-Analysis of Randomized Controlled Trials. Plos One. 2015;10(11). | Milk and dairy | population: Japanese only |
| 15 | Chen SW, Chen ZH, Liang YH, Wang P, Peng JW. Elevated hypertension risk associated with higher dietary acid load: A systematic review and meta-analysis. Clin Nutr ESPEN. 2019;33:171-7. | Patterns of diet | fewer studies than chosen one |
| 16 | Cicero AF, Aubin F, Azais-Braesco V, Borghi C. Do the lactotripeptides isoleucine-proline-proline and valine-proline-proline reduce systolic blood pressure in European subjects? A meta-analysis of randomized controlled trials. Am J Hypertens. 2013;26(3):442-9. | Milk and dairy | population: European only |
| 17 | Cicero AF, Gerocarni B, Laghi L, Borghi C. Blood pressure lowering effect of lactotripeptides assumed as functional foods: a meta-analysis of current available clinical trials. J Hum Hypertens. 2011;25(7):425-36. | Milk and dairy | fewer studies than chosen one |
| 18 | Cole NI, Swift PA, He FJ, MacGregor GA, Suckling RJ. The effect of dietary salt on blood pressure in individuals receiving chronic dialysis: a systematic review and meta-analysis of randomised controlled trials. J Hum Hypertens. 2019;33(4):319-26. | Minerals | population: ESRD adults |
| 19 | Daneshzad E, Haghighatdoost F, Azadbakht L. Dietary acid load and cardiometabolic risk factors: a systematic review and meta-analysis of observational studies. Public Health Nutr. 2019;22(15):2823-34. | Patterns of diet | fewer studies than chosen one |
| 20 | de Paula TP, Kramer CK, Viana LV, Azevedo MJ. Effects of individual micronutrients on blood pressure in patients with type 2 diabetes: a systematic review and meta-analysis of randomized clinical trials. Sci Rep. 2017;7:40751. | Vitamins (vitamin D) | populatoin: prediabetes, T2DM |
| 21 | Del Gobbo LC, Falk MC, Feldman R, Lewis K, Mozaffarian D. Effects of tree nuts on blood lipids, apolipoproteins, and blood pressure: systematic review, meta-analysis, and dose-response of 61 controlled intervention trials. Am J Clin Nutr. 2015;102(6):1347-56. | Nuts and seeds | fewer studies than chosen one |
| 22 | D'Elia L, Cairella G, Scalfi L, Garbagnati F, Rossi G, Strazzullo P. Coffee intake and risk of hypertension and stroke: Meta-analysis of prospective studies. High Blood Pressure and Cardiovascular Prevention. 2012;19(3):153. | Beverages | fewer studies than chosen one |
| 23 | D'Elia L, La Fata E, Galletti F, Scalfi L, Strazzullo P. Coffee consumption and risk of hypertension: a dose-response meta-analysis of prospective studies. Eur J Nutr. 2019;58(1):271-80. | Beverages | fewer studies than chosen one |
| 24 | Desch S, Schmidt J, Kobler D, Sonnabend M, Eitel I, Sareban M, et al. Effect of cocoa products on blood pressure: systematic review and meta-analysis. Am J Hypertens. 2010;23(1):97-103. | Cocoa | fewer studies than chosen one |
| 25 | Dibaba DT, Xun P, Song Y, Rosanoff A, Shechter M, He K. The effect of magnesium supplementation on blood pressure in individuals with insulin resistance, prediabetes, or noncommunicable chronic diseases: a meta-analysis of randomized controlled trials. Am J Clin Nutr. 2017;106(3):921-9. | Minerals | populatoin: prediabetes, T2DM |
| 26 | Dong JY, Qin LQ, Zhang Z, Zhao Y, Wang J, Arigoni F, et al. Effect of oral L-arginine supplementation on blood pressure: a meta-analysis of randomized, double-blind, placebo-controlled trials. Am Heart J. 2011;162(6):959-65. | Proteins | population: nomo-/hypertensive |
| 27 | Dong JY, Zhang ZL, Wang PY, Qin LQ. Effects of high-protein diets on body weight, glycaemic control, blood lipids and blood pressure in type 2 diabetes: meta-analysis of randomised controlled trials. Br J Nutr. 2013;110(5):781-9. | Patterns of diet | populatoin: prediabetes, T2DM |
| 28 | Dong Y, Xu M, Chen L, Bhochhibhoya A. Probiotic Foods and Supplements Interventions for Metabolic Syndromes: A Systematic Review and Meta-Analysis of Recent Clinical Trials. Ann Nutr Metab. 2019;74(3):224-41. | Probiotics | includes pregnant women |
| 29 | Elamin MB, Abu Elnour NO, Elamin KB, Fatourechi MM, Alkatib AA, Alm, et al. Vitamin D and cardiovascular outcomes: a systematic review and meta-analysis. J Clin Endocrinol Metab. 2011;96(7):1931-42. | Vitamins | fewer studies than chosen one |
| 30 | Evans CE, Greenwood DC, Threapleton DE, Gale CP, Cleghorn CL, Burley VJ. Glycemic index, glycemic load, and blood pressure: a systematic review and meta-analysis of randomized controlled trials. Am J Clin Nutr. 2017;105(5):1176-90. | Patterns of diet | fewer studies than chosen one |
| 31 | Faris MAE, Jahrami HA, Alsibai J, Obaideen AA. Impact of Ramadan Diurnal Intermittent Fasting on Metabolic Syndrome Components in Healthy, Non-Athletic Muslim People Aged Over 15 Years: A Systematic Review and Meta-Analysis. Br J Nutr. 2019:1-51. | Patterns of diet | population: not general population |
| 32 | Fattore E, Botta F, Agostoni C, Bosetti C. Effects of free sugars on blood pressure and lipids: a systematic review and meta-analysis of nutritional isoenergetic intervention trials. Am J Clin Nutr. 2017;105(1):42-56. | Sugars | fewer studies than chosen one |
| 33 | Fogacci F, Tocci G, Presta V, Fratter A, Borghi C, Cicero AFG. Effect of resveratrol on blood pressure: A systematic review and meta-analysis of randomized, controlled, clinical trials. Crit Rev Food Sci Nutr. 2019;59(10):1605-18. | Polyphenols | fewer studies than chosen one |
| 34 | Garofalo C, Borrelli S, Provenzano M, De Stefano T, Vita C, Chiodini P, et al. Dietary Salt Restriction in Chronic Kidney Disease: A Meta-Analysis of Randomized Clinical Trials. Nutrients. 2018;10(6). | Minerals | population: ESRD adults |
| 35 | George ES, Marshall S, Mayr HL, Trakman GL, Tatucu-Babet OA, Lassemillante ACM, et al. The effect of high-polyphenol extra virgin olive oil on cardiovascular risk factors: A systematic review and meta-analysis. Critical reviews in food science and nutrition. 2019;59(17):2772-95. | Polyphenols | fewer studies than chosen one |
| 36 | Gow ML, Ho M, Burrows TL, Baur LA, Stewart L, Hutchesson MJ, et al. Impact of dietary macronutrient distribution on BMI and cardiometabolic outcomes in overweight and obese children and adolescents: a systematic review. Nutr Rev. 2014;72(7):453-70. | Patterns of diet | population: children |
| 37 | Graudal N, Hubeck-Graudal T, Jurgens G, McCarron DA. The significance of duration and amount of sodium reduction intervention in normotensive and hypertensive individuals: a meta-analysis. Adv Nutr. 2015;6(2):169-77. | Minerals | population: nomo-/hypertensive |
| 38 | Graudal N, Hubeck-Graudal T, Jurgens G, Taylor RS. Dose-response relation between dietary sodium and blood pressure: a meta-regression analysis of 133 randomized controlled trials. Am J Clin Nutr. 2019;109(5):1273-8. | Minerals | fewer studies than chosen one |
| 39 | Graudal NA, Galloe AM, Garred P. Effects of sodium restriction on blood pressure, renin, aldosterone, catecholamines, cholesterols, and triglyceride: a meta-analysis. Jama. 1998;279(17):1383-91. | Minerals | fewer studies than chosen one |
| 40 | Graudal NA, Hubeck-Graudal T, Jurgens G. Effects of low-sodium diet vs. high-sodium diet on blood pressure, renin, aldosterone, catecholamines, cholesterol, and triglyceride (Cochrane Review). Am J Hypertens. 2012;25(1):1-15. | Minerals | fewer studies than chosen one |
| 41 | Grosso G, Micek A, Godos J, Pajak A, Sciacca S, Bes-Rastrollo M, et al. Long-Term Coffee Consumption Is Associated with Decreased Incidence of New-Onset Hypertension: A Dose-Response Meta-Analysis. Nutrients. 2017;9(8). | Beverages | fewer studies than chosen one |
| 42 | Guo K, Zhou Z, Jiang Y, Li W, Li Y. Meta-analysis of prospective studies on the effects of nut consumption on hypertension and type 2 diabetes mellitus. J Diabetes. 2015;7(2):202-12. | Nuts and seeds | fewer studies than chosen one |
| 43 | Guo XF, Li JM, Tang J, Li D. Effects of resveratrol supplementation on risk factors of non-communicable diseases: A meta-analysis of randomized controlled trials. Crit Rev Food Sci Nutr. 2018;58(17):3016-29. | Polyphenols | fewer studies than chosen one |
| 44 | He FJ, Li JF, MacGregor GA. Effect of longer term modest salt reduction on blood pressure: Cochrane systematic review and meta-analysis of randomised trials. Bmj-British Medical Journal. 2013;346. | Minerals | fewer studies than chosen one |
| 45 | He FJ, MacGregor GA. Effect of modest salt reduction on blood pressure: a meta-analysis of randomized trials. Implications for public health. J Hum Hypertens. 2002;16(11):761-70. | Minerals | fewer studies than chosen one |
| 46 | He FJ, MacGregor GA. Effect of longer-term modest salt reduction on blood pressure. Cochrane Database Syst Rev. 2004(3):Cd004937. | Minerals | fewer studies than chosen one |
| 47 | He J, Zhang F, Han Y. Effect of probiotics on lipid profiles and blood pressure in patients with type 2 diabetes: A meta-analysis of RCTs. Medicine (Baltimore). 2017;96(51):e9166. | Probiotics | fewer studies than chosen one |
| 48 | He S, Hao X. The effect of vitamin D3 on blood pressure in people with vitamin D deficiency: A system review and meta-analysis. Medicine (Baltimore). 2019;98(19):e15284. | Vitamins | population: vitamin D deficient |
| 49 | Hendijani F, Akbari V. Probiotic supplementation for management of cardiovascular risk factors in adults with type II diabetes: A systematic review and meta-analysis. Clin Nutr. 2018;37(2):532-41. | Probiotics | fewer studies than chosen one |
| 50 | Hession M, Roll, C, Kulkarni U, Wise A, Broom J. Systematic review of randomized controlled trials of low-carbohydrate vs. low-fat/low-calorie diets in the management of obesity and its comorbidities. Obes Rev. 2009;10(1):36-50. | Patterns of diet | population: obese or overweight only |
| 51 | Hibi M, Takase H, Iwasaki M, Osaki N, Katsuragi Y. Efficacy of tea catechin-rich beverages to reduce abdominal adiposity and metabolic syndrome risks in obese and overweight subjects: a pooled analysis of 6 human trials. Nutr Res. 2018;55:1-10. | Beverages | population: obese or overweight only |
| 52 | Hooper L, Bartlett C, Davey Smith G, Ebrahim S. Systematic review of long term effects of advice to reduce dietary salt in adults. Bmj. 2002;325(7365):628. | Minerals | fewer studies than chosen one |
| 53 | Hooper L, Kay C, Abdelhamid A, Kroon PA, Cohn JS, Rimm EB, et al. Effects of chocolate, cocoa, and flavan-3-ols on cardiovascular health: a systematic review and meta-analysis of randomized trials. Am J Clin Nutr. 2012;95(3):740-51. | Cocoa | fewer studies than chosen one |
| 54 | Hooper L, Kroon PA, Rimm EB, Cohn JS, Harvey I, Le Cornu KA, et al. Flavonoids, flavonoid-rich foods, and cardiovascular risk: a meta-analysis of randomized controlled trials. Am J Clin Nutr. 2008;88(1):38-50. | Polyphenols | data pooled before inclusion in meta analysis |
| 55 | Huang H, Chen G, Liao D, Zhu Y, Pu R, Xue X. The effects of resveratrol intervention on risk markers of cardiovascular health in overweight and obese subjects: a pooled analysis of randomized controlled trials. Obes Rev. 2016;17(12):1329-40. | Polyphenols | fewer studies than chosen one |
| 56 | Huang H, Chen G, Liao D, Zhu Y, Xue X. Effects of Berries Consumption on Cardiovascular Risk Factors: A Meta-analysis with Trial Sequential Analysis of Randomized Controlled Trials. Sci Rep. 2016;6:23625. | Fruits and vegetables | fewer studies than chosen one |
| 57 | Huntriss R, Campbell M, Bedwell C. The interpretation and effect of a low-carbohydrate diet in the management of type 2 diabetes: a systematic review and meta-analysis of randomised controlled trials. Eur J Clin Nutr. 2018;72(3):311-25. | Patterns of diet | populatoin: prediabetes, T2DM |
| 58 | Huo R, Du T, Xu Y, Xu W, Chen X, Sun K, et al. Effects of Mediterranean-style diet on glycemic control, weight loss and cardiovascular risk factors among type 2 diabetes individuals: a meta-analysis. Eur J Clin Nutr. 2015;69(11):1200-8. | Patterns of diet | populatoin: prediabetes, T2DM |
| 59 | Jafari Azad B, Daneshzad E, Azadbakht L. Peanut and cardiovascular disease risk factors: A systematic review and meta-analysis. Crit Rev Food Sci Nutr. 2019:1-18. | Legumes and pulses | fewer studies than chosen one |
| 60 | Jafari T, Fallah AA, Rostampour N, Mahmoodnia L. Vitamin D ameliorates systolic but not diastolic blood pressure in patients with type 2 diabetes: Results from a meta-analysis of randomized controlled trials. Int J Vitam Nutr Res. 2018;88(1):90-9. | Vitamins | populatoin: prediabetes, T2DM |
| 61 | Jayalath VH, de Souza RJ, Ha V, Mirrahimi A, Blanco-Mejia S, Di Buono M, et al. Sugar-sweetened beverage consumption and incident hypertension: a systematic review and meta-analysis of prospective cohorts. Am J Clin Nutr. 2015;102(4):914-21. | Beverages | same number of studies, but older than chosen one |
| 62 | Jee SH, He J, Whelton PK, Suh I, Klag MJ. The effect of chronic coffee drinking on blood pressure: a meta-analysis of controlled clinical trials. Hypertension. 1999;33(2):647-52. | Beverages | fewer studies than chosen one |
| 63 | Jee SH, Miller ER, 3rd, Guallar E, Singh VK, Appel LJ, Klag MJ. The effect of magnesium supplementation on blood pressure: a meta-analysis of randomized clinical trials. Am J Hypertens. 2002;15(8):691-6. | Minerals | fewer studies than chosen one |
| 64 | Kanerva N, Kaartinen NE, Rissanen H, Knekt P, Eriksson JG, Saaksjarvi K, et al. Associations of the Baltic Sea diet with cardiometabolic risk factors--a meta-analysis of three Finnish studies. Br J Nutr. 2014;112(4):616-26. | Patterns of diet | population: Finnish studies only |
| 65 | Kass L, Weekes J, Carpenter L. Effect of magnesium supplementation on blood pressure: a meta-analysis. Eur J Clin Nutr. 2012;66(4):411-8. | Minerals | fewer studies than chosen one |
| 66 | Kastorini CM, Milionis HJ, Esposito K, Giugliano D, Goudevenos JA, Panagiotakos DB. The effect of Mediterranean diet on metabolic syndrome and its components: a meta-analysis of 50 studies and 534,906 individuals. J Am Coll Cardiol. 2011;57(11):1299-313. | Patterns of diet | fewer studies than chosen one |
| 67 | Ke L, Mason RS, Kariuki M, Mpofu E, Brock KE. Vitamin D status and hypertension: a review. Integr Blood Press Control. 2015;8:13-35. | Vitamins | fewer studies than chosen one |
| 68 | Kelishadi R, Mansourian M, Heidari-Beni M. Association of fructose consumption and components of metabolic syndrome in human studies: a systematic review and meta-analysis. Nutrition. 2014;30(5):503-10. | Sugars | fewer studies than chosen one |
| 69 | Kelly J, Khalesi S, Dickinson K, Hines S, Coombes JS, Todd AS. The effect of dietary sodium modification on blood pressure in adults with systolic blood pressure less than 140 mmHg: a systematic review. JBI Database System Rev Implement Rep. 2016;14(6):196-237. | Minerals | fewer studies than chosen one |
| 70 | Khalesi S, Irwin C, Schubert M. Flaxseed consumption may reduce blood pressure: a systematic review and meta-analysis of controlled trials. J Nutr. 2015;145(4):758-65. | Nuts and seeds | fewer studies than chosen one |
| 71 | Khalesi S, Sun J, Buys N, Jamshidi A, Nikbakht-Nasrabadi E, Khosravi-Boroujeni H. Green tea catechins and blood pressure: a systematic review and meta-analysis of randomised controlled trials. Eur J Nutr. 2014;53(6):1299-311. | Beverages | fewer studies than chosen one |
| 72 | Kou T, Wang Q, Cai J, Song J, Du B, Zhao K, et al. Effect of soybean protein on blood pressure in postmenopausal women: a meta-analysis of randomized controlled trials. Food Funct. 2017;8(8):2663-71. | Proteins | fewer studies than chosen one |
| 73 | Kwak JS, Kim JY, Paek JE, Lee YJ, Kim HR, Park DS, et al. Garlic powder intake and cardiovascular risk factors: a meta-analysis of randomized controlled clinical trials. Nutr Res Pract. 2014;8(6):644-54. | Fruits and vegetables | fewer studies than chosen one |
| 74 | Lee KJ, Lee YJ. Effects of vitamin D on blood pressure in patients with type 2 diabetes mellitus. Int J Clin Pharmacol Ther. 2016;54(4):233-42. | Vitamins | populatoin: prediabetes, T2DM |
| 75 | Li B, Li F, Wang L, Zhang D. Fruit and Vegetables Consumption and Risk of Hypertension: A Meta-Analysis. J Clin Hypertens (Greenwich). 2016;18(5):468-76. | Fruits and vegetables | fewer studies than chosen one |
| 76 | Li G, Zhang Y, Thabane L, Mbuagbaw L, Liu A, Levine MA, et al. Effect of green tea supplementation on blood pressure among overweight and obese adults: a systematic review and meta-analysis. J Hypertens. 2015;33(2):243-54. | Beverages | population: obese or overweight only |
| 77 | Li X, Xu J. Lycopene supplement and blood pressure: an updated meta-analysis of intervention trials. Nutrients. 2013;5(9):3696-712. | Phytochemicals | fewer studies than chosen one |
| 78 | Liu XX, Li SH, Hui RT. Effect of soy isoflavones on blood pressure. A meta-analysis of randomized controlled trials. Journal of Molecular and Cellular Cardiology. 2010;48(5):S82. | Proteins | fewer studies than chosen one |
| 79 | Liu Y, Ma W, Zhang P, He S, Huang D. Effect of resveratrol on blood pressure: a meta-analysis of randomized controlled trials. Clin Nutr. 2015;34(1):27-34. | Polyphenols | fewer studies than chosen one |
| 80 | Ma XM, Xu Y. The association between breakfast skipping and the risk of obesity, diabetes, hypertension, or dyslipidemia-a meta-analysis from 44 trials including 65,233 cases and 381,051 controls. Diabetes. 2018;67:A364. | Patterns of diet | fewer studies than chosen one |
| 81 | Manousopoulou A, Al-Daghri NM, Garbis SD, Chrousos GP. Vitamin D and cardiovascular risk among adults with obesity: a systematic review and meta-analysis. Eur J Clin Invest. 2015;45(10):1113-26. | Vitamins | fewer studies than chosen one |
| 82 | Mansoor N, Vinknes KJ, Veierod MB, Retterstol K. Effects of low-carbohydrate diets v. low-fat diets on body weight and cardiovascular risk factors: a meta-analysis of randomised controlled trials. Br J Nutr. 2016;115(3):466-79. | Patterns of diet | fewer studies than chosen one |
| 83 | Marx W, Kelly J, Marshall S, Nakos S, Campbell K, Itsiopoulos C. The Effect of Polyphenol-Rich Interventions on Cardiovascular Risk Factors in Haemodialysis: A Systematic Review and Meta-Analysis. Nutrients. 2017;9(12). | Polyphenols | population: ESRD adults |
| 84 | Mejia SB, Kendall CW, Viguiliouk E, Augustin LS, Ha V, Cozma AI, et al. Tree nut consumption on metabolic syndrome criteria: A systematic review and meta-analysis of randomized controlled trials. Canadian Journal of Diabetes. 2015;39:S56. | Nuts and seeds | fewer studies than chosen one |
| 85 | Mesas AE, Leon-Munoz L, Rodriguez-Artalejo F, Lopez-Garcia E. The effect of coffee on blood pressure and cardiovascular disease among hypertensive individuals: Meta-analysis. Journal of Clinical Hypertension. 2011;13(4):A42. | Beverages | abstract |
| 86 | Mesas AE, Leon-Munoz LM, Rodriguez-Artalejo F, Lopez-Garcia E. The effect of coffee on blood pressure and cardiovascular disease in hypertensive individuals: a systematic review and meta-analysis. Am J Clin Nutr. 2011;94(4):1113-26. | Beverages | fewer studies than chosen one |
| 87 | Midgley JP, Matthew AG, Greenwood CM, Logan AG. Effect of reduced dietary sodium on blood pressure: a meta-analysis of randomized controlled trials. Jama. 1996;275(20):1590-7. | Minerals | fewer studies than chosen one |
| 88 | Nissensohn M, Roman-Vinas B, Sanchez-Villegas A, Piscopo S, Serra-Majem L. The Effect of the Mediterranean Diet on Hypertension: A Systematic Review and Meta-Analysis. J Nutr Educ Behav. 2016;48(1):42-53.e1. | Patterns of diet | fewer studies than chosen one |
| 89 | Nordmann AJ, Nordmann A, Briel M, Keller U, Yancy WS, Jr., Brehm BJ, et al. Effects of low-carbohydrate vs low-fat diets on weight loss and cardiovascular risk factors: a meta-analysis of randomized controlled trials. Arch Intern Med. 2006;166(3):285-93. | Patterns of diet | fewer studies than chosen one |
| 90 | Nordmann AJ, Suter-Zimmermann K, Bucher HC, Shai I, Tuttle KR, Estruch R, et al. Meta-analysis comparing Mediterranean to low-fat diets for modification of cardiovascular risk factors. Am J Med. 2011;124(9):841-51.e2. | Patterns of diet | fewer studies than chosen one |
| 91 | Peng X, Zhou R, Wang B, Yu X, Yang X, Liu K, et al. Effect of green tea consumption on blood pressure: a meta-analysis of 13 randomized controlled trials. Sci Rep. 2014;4:6251. | Beverages | fewer studies than chosen one |
| 92 | Peng YG, Li W, Wen XX, Li Y, Hu JH, Zhao LC. Effects of salt substitutes on blood pressure: a meta-analysis of randomized controlled trials. Am J Clin Nutr. 2014;100(6):1448-54. | Minerals | fewer studies than chosen one |
| 93 | Penninkilampi R, Eslick EM, Eslick GD. The association between consistent licorice ingestion, hypertension and hypokalaemia: a systematic review and meta-analysis. Journal of Human Hypertension. 2017;31(11):699-707. | Herb, spice, and condiment | fewer studies than chosen one |
| 94 | Pittas AG, Chung M, Trikalinos T, Mitri J, Brendel M, Patel K, et al. Systematic review: Vitamin D and cardiometabolic outcomes. Ann Intern Med. 2010;152(5):307-14. | Vitamins | fewer studies than chosen one |
| 95 | Poorolajal J, Zeraati F, Soltanian AR, Sheikh V, Hooshm, E, et al. Oral potassium supplementation for management of essential hypertension: A meta-analysis of randomized controlled trials. PLoS One. 2017;12(4):e0174967. | Minerals | fewer studies than chosen one |
| 96 | Qi D, Nie X, Cai J. The effect of vitamin D supplementation on hypertension in non-CKD populations: A systemic review and meta-analysis. Int J Cardiol. 2017;227:177-86. | Vitamins | fewer studies than chosen one |
| 97 | Qin LQ, Xu JY, Dong JY, Zhao Y, van Bladeren P, Zhang W. Lactotripeptides intake and blood pressure management: a meta-analysis of randomised controlled clinical trials. Nutr Metab Cardiovasc Dis. 2013;23(5):395-402. | Milk and dairy | fewer studies than chosen one |
| 98 | Reinhart KM, Coleman CI, Teevan C, Vachhani P, White CM. Effects of garlic on blood pressure in patients with and without systolic hypertension: a meta-analysis. Ann Pharmacother. 2008;42(12):1766-71. | Fruits and vegetables | fewer studies than chosen one |
| 99 | Ried K, Fakler P. Protective effect of lycopene on serum cholesterol and blood pressure: Meta-analyses of intervention trials. Maturitas. 2011;68(4):299-310. | Phytochemicals | fewer studies than chosen one |
| 100 | Ried K, Frank OR, Stocks NP, Fakler P, Sullivan T. Effect of garlic on blood pressure: a systematic review and meta-analysis. BMC Cardiovasc Disord. 2008;8:13. | Fruits and vegetables | fewer studies than chosen one |
| 101 | Ried K, Sullivan T, Fakler P, Frank OR, Stocks NP. Does chocolate reduce blood pressure? A meta-analysis. BMC Med. 2010;8:39. | Cocoa | population: nomo-/hypertensive |
| 102 | Rohner A, Ried K, Sobenin IA, Bucher HC, Nordmann AJ. A systematic review and metaanalysis on the effects of garlic preparations on blood pressure in individuals with hypertension. Am J Hypertens. 2015;28(3):414-23. | Fruits and vegetables | fewer studies than chosen one |
| 103 | Rosticci M, Cicero AFG, Veronesi M, Gerocarni B, Laghi L, Borghi C. Blood pressure lowering effect of lactotripeptides consumed as functional foods: A meta-analysis of currently available clinical trials. High Blood Pressure and Cardiovascular Prevention. 2010;17(3):141. | Milk and dairy | fewer studies than chosen one |
| 104 | Sahebkar A, Serban C, Ursoniu S, Wong ND, Muntner P, Graham IM, et al. Lack of efficacy of resveratrol on C-reactive protein and selected cardiovascular risk factors--Results from a systematic review and meta-analysis of randomized controlled trials. Int J Cardiol. 2015;189:47-55. | Polyphenols | fewer studies than chosen one |
| 105 | Saneei P, Salehi-Abargouei A, Esmaillzadeh A, Azadbakht L. Influence of Dietary Approaches to Stop Hypertension (DASH) diet on blood pressure: a systematic review and meta-analysis on randomized controlled trials. Nutr Metab Cardiovasc Dis. 2014;24(12):1253-61. | Patterns of diet | fewer studies than chosen one |
| 106 | Santos FL, Esteves SS, da Costa Pereira A, Yancy WS, Jr., Nunes JP. Systematic review and meta-analysis of clinical trials of the effects of low carbohydrate diets on cardiovascular risk factors. Obes Rev. 2012;13(11):1048-66. | Patterns of diet | fewer studies than chosen one |
| 107 | Schwingshackl L, Schwedhelm C, Hoffmann G, Knuppel S, Iqbal K, Andriolo V, et al. Food Groups and Risk of Hypertension: A Systematic Review and Dose-Response Meta-Analysis of Prospective Studies. Adv Nutr. 2017;8(6):793-803. | Meat, poultry, egg, and fish | fewer studies than chosen one |
| 108 | Serban MC, Sahebkar A, Zanchetti A, Mikhailidis DP, Howard G, Antal D, et al. Effects of Quercetin on Blood Pressure: A Systematic Review and Meta-Analysis of Randomized Controlled Trials. J Am Heart Assoc. 2016;5(7). | Polyphenols | fewer studies than chosen one |
| 109 | Shu L, Huang K. Effect of vitamin D supplementation on blood pressure parameters in patients with vitamin D deficiency: a systematic review and meta-analysis. J Am Soc Hypertens. 2018;12(7):488-96. | Vitamins | fewer studies than chosen one |
| 110 | Siervo M, Lara J, Ogbonmwan I, Mathers JC. Inorganic nitrate and beetroot juice supplementation reduces blood pressure in adults: a systematic review and meta-analysis. J Nutr. 2013;143(6):818-26. | Nitrates | fewer studies than chosen one |
| 111 | Silagy CA, Neil HA. A meta-analysis of the effect of garlic on blood pressure. J Hypertens. 1994;12(4):463-8. | Fruits and vegetables | fewer studies than chosen one |
| 112 | Sood N, Baker WL, Coleman CI. Effect of glucomannan on plasma lipid and glucose concentrations, body weight, and blood pressure: systematic review and meta-analysis. Am J Clin Nutr. 2008;88(4):1167-75. | Carbohydrates | fewer studies than chosen one |
| 113 | Streppel MT, Arends LR, van 't Veer P, Grobbee DE, Geleijnse JM. Dietary fiber and blood pressure: a meta-analysis of randomized placebo-controlled trials. Arch Intern Med. 2005;165(2):150-6. | Carbohydrates | fewer studies than chosen one |
| 114 | Swart KM, Lips P, Brouwer IA, Jorde R, Heymans MW, Grimnes G, et al. Effects of vitamin D supplementation on markers for cardiovascular disease and type 2 diabetes: an individual participant data meta-analysis of randomized controlled trials. Am J Clin Nutr. 2018;107(6):1043-53. | Vitamins | fewer studies than chosen one |
| 115 | Taku K, Lin N, Cai D, Hu J, Zhao X, Zhang Y, et al. Effects of soy isoflavone extract supplements on blood pressure in adult humans: Systematic review and meta-analysis of randomized placebo-controlled trials. Atherosclerosis Supplements. 2010;11(2):212. | Proteins | fewer studies than chosen one |
| 116 | Talukder MR, Rutherford S, Huang C, Phung D, Islam MZ, Chu C. Drinking water salinity and risk of hypertension: A systematic review and meta-analysis. Arch Environ Occup Health. 2017;72(3):126-38. | Minerals | population: children |
| 117 | Tamtaji OR, Milajerdi A, Dadgostar E, Kolahdooz F, Chamani M, Amirani E, et al. The Effects of Quercetin Supplementation on Blood Pressures and Endothelial Function Among Patients with Metabolic Syndrome and Related Disorders: A Systematic Review and Meta-analysis of Randomized Controlled Trials. Curr Pharm Des. 2019;25(12):1372-84. | Polyphenols | fewer studies than chosen one |
| 118 | Taubert D, Roesen R, Schomig E. Effect of cocoa and tea intake on blood pressure: a meta-analysis. Arch Intern Med. 2007;167(7):626-34. | Cocoa | population: nomo-/hypertensive |
| 119 | Taylor B, Irving HM, Baliunas D, Roerecke M, Patra J, Mohapatra S, et al. Alcohol and hypertension: gender differences in dose-response relationships determined through systematic review and meta-analysis. Addiction. 2009;104(12):1981-90. | Beverages | fewer studies than chosen one |
| 120 | Taylor RS, Ashton KE, Moxham T, Hooper L, Ebrahim S. Reduced dietary salt for the prevention of cardiovascular disease: a meta-analysis of randomized controlled trials (Cochrane review). Am J Hypertens. 2011;24(8):843-53. | Minerals | fewer studies than chosen one |
| 121 | Tielemans SM, Altorf-van der Kuil W, Engberink MF, Brink EJ, van Baak MA, Bakker SJ, et al. Intake of total protein, plant protein and animal protein in relation to blood pressure: a meta-analysis of observational and intervention studies. J Hum Hypertens. 2013;27(9):564-71. | Proteins | fewer studies than chosen one |
| 122 | Toews I, Lohner S, de Gaudry DK, Sommer H, Meerpohl JJ. Association between intake of non-sugar sweeteners and health outcomes: systematic review and meta-analyses of randomised and non-randomised controlled trials and observational studies. Bmj-British Medical Journal. 2019;364. | Sweeteners | fewer studies than chosen one |
| 123 | Turpeinen AM, Jarvenpaa S, Kautiainen H, Korpela R, Vapaatalo H. Antihypertensive effects of bioactive tripeptides-a random effects meta-analysis. Ann Med. 2013;45(1):51-6. | Milk and dairy | fewer studies than chosen one |
| 124 | van Bommel E, Cleophas T. Potassium treatment for hypertension in patients with high salt intake: a meta-analysis. Int J Clin Pharmacol Ther. 2012;50(7):478-82. | Minerals | fewer studies than chosen one |
| 125 | van Mierlo LA, Arends LR, Streppel MT, Zeegers MP, Kok FJ, Grobbee DE, et al. Blood pressure response to calcium supplementation: a meta-analysis of randomized controlled trials. J Hum Hypertens. 2006;20(8):571-80. | Minerals | fewer studies than chosen one |
| 126 | van Zuuren EJ, Fedorowicz Z, Kuijpers T, Pijl H. Effects of low-carbohydrate- compared with low-fat-diet interventions on metabolic control in people with type 2 diabetes: a systematic review including GRADE assessments. Am J Clin Nutr. 2018;108(2):300-31. | Patterns of diet | populatoin: prediabetes, T2DM |
| 127 | Verma H, Garg R. Effect of magnesium supplementation on type 2 diabetes associated cardiovascular risk factors: a systematic review and meta-analysis. J Hum Nutr Diet. 2017;30(5):621-33. | Minerals | populatoin: prediabetes, T2DM |
| 128 | Verma H, Garg R. Effect of Vitamin K Supplementation on Cardiometabolic Risk Factors: A Systematic Review and Meta-Analysis. Endocr Metab Immune Disord Drug Targets. 2019;19(1):13-25. | Vitamins | population: postmenoposal only |
| 129 | Waldron M, Patterson SD, Tallent J, Jeffries O. The Effects of Oral Taurine on Resting Blood Pressure in Humans: a Meta-Analysis. Curr Hypertens Rep. 2018;20(9):81. | Proteins | fewer studies than chosen one |
| 130 | Wang HP, Yang J, Qin LQ, Yang XJ. Effect of garlic on blood pressure: a meta-analysis. J Clin Hypertens (Greenwich). 2015;17(3):223-31. | Fruits and vegetables | fewer studies than chosen one |
| 131 | Wang M, Moran AE, Liu J, Qi Y, Xie W, Tzong K, et al. A Meta-Analysis of Effect of Dietary Salt Restriction on Blood Pressure in Chinese Adults. Glob Heart. 2015;10(4):291-9.e6. | Minerals | fewer studies than chosen one |
| 132 | Whelton PK, He J, Cutler JA, Brancati FL, Appel LJ, Follmann D, et al. Effects of oral potassium on blood pressure. Meta-analysis of randomized controlled clinical trials. Jama. 1997;277(20):1624-32. | Minerals | fewer studies than chosen one |
| 133 | Witham MD, Nadir MA, Struthers AD. Effect of vitamin D on blood pressure: a systematic review and meta-analysis. J Hypertens. 2009;27(10):1948-54. | Vitamins | fewer studies than chosen one |
| 134 | Wu SH, Ho SC, Zhong L. Effects of vitamin D supplementation on blood pressure. South Med J. 2010;103(8):729-37. | Vitamins | fewer studies than chosen one |
| 135 | Wycherley TP, Moran LJ, Clifton PM, Noakes M, Brinkworth GD. Effects of energy-restricted high-protein, low-fat compared with standard-protein, low-fat diets: a meta-analysis of randomized controlled trials. Am J Clin Nutr. 2012;96(6):1281-98. | Patterns of diet | fewer studies than chosen one |
| 136 | Xi B, Huang Y, Reilly KH, Li S, Zheng R, Barrio-Lopez MT, et al. Sugar-sweetened beverages and risk of hypertension and CVD: a dose-response meta-analysis. Br J Nutr. 2015;113(5):709-17. | Beverages | fewer studies than chosen one |
| 137 | Xin X, He J, Frontini MG, Ogden LG, Motsamai OI, Whelton PK. Effects of alcohol reduction on blood pressure: a meta-analysis of randomized controlled trials. Hypertension. 2001;38(5):1112-7. | Beverages | fewer studies than chosen one |
| 138 | Xiong XJ, Wang PQ, Li SJ, Li XK, Zhang YQ, Wang J. Garlic for hypertension: A systematic review and meta-analysis of randomized controlled trials. Phytomedicine. 2015;22(3):352-61. | Fruits and vegetables | fewer studies than chosen one |
| 139 | Yaguang P, Xiaoxiao W, Ying L, Wei L, Liancheng Z. Effect of long-term salt substitutes on blood pressure: A meta-analysis of randomized control trails. Cardiology (Switzerland). 2014;129:1. | Minerals | fewer studies than chosen one |
| 140 | Yang L, Ling W, Du Z, Chen Y, Li D, Deng S, et al. Effects of Anthocyanins on Cardiometabolic Health: A Systematic Review and Meta-Analysis of Randomized Controlled Trials. Adv Nutr. 2017;8(5):684-93. | Polyphenols | fewer studies than chosen one |
| 141 | Yarmolinsky J, Gon G, Edwards P. Effect of tea on blood pressure for secondary prevention of cardiovascular disease: a systematic review and meta-analysis of randomized controlled trials. Nutr Rev. 2015;73(4):236-46. | Beverages | fewer studies than chosen one |
| 142 | Yu Z, Nan F, Wang LY, Jiang H, Chen W, Jiang Y. Effects of high-protein diet on glycemic control, insulin resistance and blood pressure in type 2 diabetes: A systematic review and meta-analysis of randomized controlled trials. Clin Nutr. 2019. | Patterns of diet | populatoin: prediabetes, T2DM |
| 143 | Zhang HL, Liu S, Li L, Liu SS, Liu SQ, Mi J, et al. The impact of grape seed extract treatment on blood pressure changes A meta-analysis of 16 randomized controlled trials. Medicine. 2016;95(33). | Fruits and vegetables | fewer studies than chosen one |
| 144 | Zhang Z, Hu G, Caballero B, Appel L, Chen L. Habitual coffee consumption and risk of hypertension: a systematic review and meta-analysis of prospective observational studies. Am J Clin Nutr. 2011;93(6):1212-9. | Beverages | fewer studies than chosen one |
| 145 | Zhou D, Yu H, He F, Reilly KH, Zhang J, Li S, et al. Nut consumption in relation to cardiovascular disease risk and type 2 diabetes: a systematic review and meta-analysis of prospective studies. Am J Clin Nutr. 2014;100(1):270-7. | Nuts and seeds | fewer studies than chosen one |
| 146 | Zhu Y, Bo Y, Wang X, Lu W, Wang X, Han Z, et al. The Effect of Anthocyanins on Blood Pressure: A PRISMA-Compliant Meta-Analysis of Randomized Clinical Trials. Medicine (Baltimore). 2016;95(15):e3380. | Polyphenols | fewer studies than chosen one |
| 147 | Abolfazl Lari, Somaye Fatahi, Mohammad Hassan Sohouli, Farzad Shidfar. The Impact of Chromium Supplementation on Blood Pressure: A Systematic Review and Dose-Response Meta‑Analysis of Randomized‑Controlled Trials. High Blood Press Cardiovasc Prev. 2021 Jul;28(4):333-342. | Minerals | fewer studies than chosen one |
| 148 | Omid Asbaghi, Fatemeh Naeini, Damoon Ashtary-Larky, Mojtaba Kaviani, et al. Effects of chromium supplementation on blood pressure, body mass index, liver function enzymes and malondialdehyde in patients with type 2 diabetes: A systematic review and dose-response meta-analysis of randomized controlled trials. Complement Ther Med. 2021 Aug;60:102755. | Minerals | populatoin: prediabetes, T2DM |
| 149 | Wisit Cheungpasitporn, Charat Thongprayoon, Peter J Edmonds, Narat Srivali, et al. Sugar and artificially sweetened soda consumption linked to hypertension: a systematic review and meta-analysis. Clin Exp Hypertens . 2015;37(7):587-93. | Beverages | fewer studies than chosen one |
| 150 | Karen Rees, Lena Al-Khudairy, Andrea Takeda, Saverio Stranges . Vegan dietary pattern for the primary and secondary prevention of cardiovascular diseases. Cochrane Database Syst Rev. 2021 Feb 25;2(2):CD013501. | Patterns of diet | fewer studies than chosen one |
| 151 | J Xu, L Qin, P Want, W Li, and C Chang. Effect of milk tripeptides on blood pressure: a meta-analysis of randomized controlled trials. Nutrition 2008; 24(10):933-40. | Proteins | fewer studies than chosen one |
| 152 | Gowri Raman, Esther E Avendano, Siyu Chen, Jiaqi Wang, et al. Dietary intakes of flavan-3-ols and cardiometabolic health: systematic review and meta-analysis of randomized trials and prospective cohort studies. Am J Clin Nutr. 2019; 110(5):1067-1078. | Polyphenols | fewer studies than chosen one (only OS superseeded) |
| 153 | Elena Jovanovski, Any de Castro Ruiz Marques, Dandan Li, Hoang V T Ho, et al. Effect of high-carbohydrate or high‐monounsaturated fatty acid diets on blood pressure: a systematic review and meta-analysis of randomized controlled trials. Nutr Rev. 2019 Jan; 77(1): 19–31. | Patterns of diet | fewer studies than chosen one |
| 154 | Emad H Kutbi, Mohammad Hassan Sohouli, Somaye Fatahi, Abolfazl Lari. The beneficial effects of cinnamon among patients with metabolic diseases: A systematic review and dose-response meta-analysis of randomized-controlled trials. Crit Rev Food Sci Nutr. 2022;62(22):6113-6131. | Herb, spice, and condiment | fewer studies than chosen one |
| 155 | Nicole Martin, Roberta Germanò, Louise Hartley, Alma J Adler, et al. Nut consumption for the primary prevention of cardiovascular disease. Cochrane Database Syst Rev. 2015 Sep 28;2015(9):CD011583. | Nuts and seeds | fewer studies than chosen one |
| 156 | Zahra Aslani, Omid Sadeghi, Motahar Heidari-Beni, Hoda Zahedi, et al. Association of dietary inflammatory potential with cardiometabolic risk factors and diseases: a systematic review and dose-response meta-analysis of observational studies. Diabetol Metab Syndr; 2020, 7;12:86. | Patterns of diet | fewer studies than chosen one |
| 157 | Munji Choi, Seongmin Park, Myoungsook Lee. L-Carnitine's Effect on the Biomarkers of Metabolic Syndrome: A Systematic Review and Meta-Analysis of Randomized Controlled Trials. Nutrients. 2020 Sep 12;12(9):2795. | Proteins | fewer studies than chosen one |
| 158 | Gabriela Cormick, Agustín Ciapponi, María Luisa Cafferata, José M Belizán. Calcium supplementation for prevention of primary hypertension. Cochrane Database Syst Rev. 2015 Jun 30;2015(6):CD010037. | Minerals | fewer studies than chosen one |
| 159 | Owen R Cowell, Nathan Mistry, Kevin Deighton, Jamie Matu, et al. Effects of a Mediterranean diet on blood pressure: a systematic review and meta-analysis of randomized controlled trials and observational studies. J Hypertens. 2021 Apr 1;39(4):729-739. | Patterns of diet | fewer studies than chosen one |
| 160 | Ejtahed, H. S., Ardeshirlarijani, E., Tabatabaei-Malazy, O., Hoseini-Tavassol, Z., Hasani-Ranjbar, S., Soroush, A. R., & Larijani, B. (2020, Jun). Effect of probiotic foods and supplements on blood pressure: a systematic review of meta-analyses studies of controlled trials. J Diabetes Metab Disord, 19(1), 617-623 | Probiotics | fewer studies than chosen one |
| 161 | Farapti, F., Fadilla, C., Yogiswara, N., & Adriani, M. (2020). Effects of vitamin D supplementation on 25(OH)D concentrations and blood pressure in the elderly: a systematic review and meta-analysis. F1000Res, 9, 633. | Vitamins | fewer studies than chosen one |
| 162 | Elena Fattore, Francesca Botta, Cristina Bosetti. Effect of fructose instead of glucose or sucrose on cardiometabolic markers: a systematic review and meta-analysis of isoenergetic intervention trials. Nutr Rev. 2021 Jan 9;79(2):209-226. | Sugars | fewer studies than chosen one |
| 163 | Qi Gao, Li-Qiang Qin, Ahmed Arafa, Ehab S Eshak, et al. Effects of strawberry intervention on cardiovascular risk factors: a meta-analysis of randomised controlled trials. Br J Nutr. 2020 Aug 14;124(3):241-246. | Fruits and vegetables | fewer studies than chosen one |
| 164 | Matin Ghanavati, Jamal Rahmani, Cain C T Clark, Susan Mohammadi Hosseinabadi, et al. Pistachios and cardiometabolic risk factors: A systematic review and meta-analysis of randomized controlled clinical trials. Complement Ther Med. 2020 Aug;52:102513. | Nuts and seeds | fewer studies than chosen one |
| 165 | Yuanyuan Guan, Pengju Dai, Hongwu Wang. Effects of vitamin C supplementation on essential hypertension: A systematic review and meta-analysis. Medicine (Baltimore). 2020 Feb;99(8):e19274. | Vitamins | fewer studies than chosen one |
| 166 | Ebuwa Igho-Osagie, Kelly Cara, Deena Wang, Qisi Yao , et al. Short-Term Tea Consumption Is Not Associated with a Reduction in Blood Lipids or Pressure: A Systematic Review and Meta-Analysis of Randomized Controlled Trials. J Nutr. 2020 Dec 10;150(12):3269-3279. | Beverages | fewer studies than chosen one |

**Supplementary Table 3 Number of meta-analyses and publications by diet exposure and study type^1^**

| Diet exposure | Study type | Number of meta-analyses | Number of publications | Reference |
| --- | --- | --- | --- | --- |
| *Patterns of diet* | RCT | 62 | 23 | (Baye et al., 2017; Bonnet, 2020; Bueno et al., 2013; Chiavaroli et al., 2018; Dong, 2020; Filippou et al., 2020; Filippou, 2021; Gay et al., 2016; Ghaedi et al., 2019; Gibbs, 2021; Hu et al., 2012; Kirkham, 2021; Lee, 2020; Lopez et al., 2019; Mazzaro et al., 2014; Naude et al., 2014; Ramezani-Jolfaie et al., 2019; Santesso et al., 2012; Schwingshackl & Hoffmann, 2013; Schwingshackl et al., 2011; Shah et al., 2007; Toh, 2020; Yang, 2021) |
|  | OS | 15 | 7 | (Benatar & Stewart, 2018; Dehghan & Abbasalizad Farhangi, 2020; Farhangi et al., 2020; Godos et al., 2017; Parohan et al., 2019; Picasso et al., 2019; Wang et al., 2016) |
| *Meat, poultry, egg, and fish* | RCT | 6 | 3 | (Alhassan et al., 2017; Kolahdouz-Mohammadi, 2020; O'Connor et al., 2017) |
|  | OS | 6 | 2 | (Schwingshackl et al., 2017; Zhang & Zhang, 2018) |
| *Milk and dairy product* | RCT | 6 | 2 | (Benatar et al., 2013; Fekete et al., 2015) |
|  | OS | 11 | 5 | (Heidari, 2021; Lee et al., 2018; Ralston et al., 2012; Schwingshackl et al., 2017; Soedamah-Muthu et al., 2012) |
| *Fruits and vegetables* | | | | |
| Fruits | RCT | 28 | 14 | (Carvalho, 2021; D'Elia, 2021; Guo et al., 2017; Hadi et al., 2019; Han, 2020; Hawkins et al., 2021; Liu et al., 2013; Luis et al., 2018a; Motallaei, 2021; Onakpoya et al., 2017; Onakpoya, O'Sullivan, et al., 2015; Pourmasoumi et al., 2020; Sahebkar et al., 2017; Suksomboon et al., 2019) |
|  | OS | 1 | 1 | (Wu et al., 2016) |
| Vegetables | RCT | 10 | 5 | (Bahadoran et al., 2017; Hasani et al., 2019; Jandari, 2020; Ried, 2016; Wang, 2020) |
|  | OS | 1 | 1 | (Wu et al., 2016) |
| Fruits and vegetables | RCT | 2 | 1 | (Shin et al., 2015) |
|  | OS | 1 | 1 | (Wu et al., 2016) |
| *Starchy foods* | RCT | 2 | 2 | (Kelly et al., 2017; Reynolds et al., 2019) |
|  | OS | 5 | 2 | (Schwingshackl et al., 2019; Schwingshackl et al., 2017) |
| *Legumes and pulses* | RCT | 6 | 2 | (Jayalath, de Souza, et al., 2014; Mohammadifard et al., 2015) |
|  | OS | 1 | 1 | (Schwingshackl et al., 2017) |
| *Nuts and seeds* | RCT | 20 | 9 | (Asbaghi, Hadi, et al., 2021; Blanco Mejia et al., 2014; Eslampour, 2020; Huang, 2021; Jalali, 2020; Li, 2020; Sahebkar et al., 2016; Teoh et al., 2018; Ursoniu et al., 2016) |
|  | OS | 1 | 1 | (Schwingshackl et al., 2017) |
| *Cocoa* | RCT | 2 | 1 | (Ried et al., 2017) |
|  | OS | 1 | 1 | (Morze et al., 2020) |
| *Herb, spice, and condiment* | RCT | 10 | 4 | (Ashtary-Larky, 2021; Luis et al., 2018b; Mousavi et al., 2019; Setayesh, 2021) |
| *Beverages* |  |  |  |  |
| Alcohol | RCT | 4 | 2 | (Roerecke et al., 2017; Spaggiari, 2020) |
|  | OS | 2 | 1 | (Roerecke et al., 2018) |
| Coffee | RCT | 4 | 2 | (Ramli, 2021; Steffen et al., 2012) |
|  | OS | 1 | 1 | (Xie et al., 2018) |
| Tea | RCT | 8 | 4 | (Liu et al., 2014; Ma, 2021; Serban et al., 2015; Xu, 2020) |
| Soft drinks | OS | 3 | 2 | (Azad et al., 2017; Kim & Je, 2016) |
| *Carbohydrates* | RCT | 22 | 7 | (Clark, 2020; Evans et al., 2015; Faghihimani, 2021; Hashemi Tari, 2021; Khan et al., 2018; Moraru et al., 2018; Whelton et al., 2005) |
| *Sugars* | RCT | 4 | 2 | (Ha et al., 2012; Te Morenga et al., 2014) |
|  | OS | 1 | 1 | (Jayalath, Sievenpiper, et al., 2014) |
| *Proteins* | RCT | 24 | 9 | (Askarpour et al., 2019; Badely et al., 2019; Dong et al., 2011; Guan, 2020; Hidayat et al., 2017; Mirenayat et al., 2018; Pripp, 2008; Rebholz et al., 2012; Sohouli, 2021) |
|  | OS | 2 | 1 | (Liu et al., 2002) |
| *Fats and oils* | RCT | 15 | 7 | (AbuMweis et al., 2018; Guo et al., 2019; Miller et al., 2014; Morris et al., 1993; Wendland et al., 2006; Yang et al., 2015; Zamora-Zamora et al., 2018) |
|  | OS | 3 | 1 | (Yang et al., 2016) |
| *Minerals* | RCT | 20 | 10 | (Aburto et al., 2013; Allender et al., 1996; Geleijnse et al., 2003; Ghanbari, 2021; Griffith et al., 1999; Hernandez et al., 2019; Khazdouz et al., 2020; Ndanuko, 2021; Whelton et al., 1997; Zhang et al., 2016) |
|  | RCT - hypertension | 2 | 1 | (Beyer et al., 2006) |
|  | OS | 5 | 4 | (Han et al., 2017; Jayedi & Zargar, 2019; Kuruppu et al., 2014; Ziaei, 2020) |
| *Vitamins* | RCT | 18 | 9 | (Asbaghi, 2021; Emami et al., 2019; Golzarand et al., 2016; Juraschek et al., 2012; Li et al., 2018; X. H. Li et al., 2015; Mirhosseini et al., 2018; Tabrizi et al., 2018; Wu & Sun, 2017) |
|  | OS | 4 | 2 | (Kunutsor et al., 2013; Ran, 2020) |
| *Probiotics* | RCT | 6 | 3 | (Ghavami, 2020; Lewis-Mikhael, 2020; Qi, 2020) |
| *Polyphenols* | RCT | 28 | 13 | (Akbari et al., 2019; Asbaghi, Naeini, et al., 2021; Daneshzad et al., 2019; Feringa et al., 2011; Garcia-Conesa et al., 2018; Hohmann et al., 2015; S. H. Li et al., 2015; Menezes et al., 2017; Mohammadi et al., 2019; Raman et al., 2019; Ren, 2021; Shrime et al., 2011; Weaver, 2021) |
|  | RCT - hypertension | 4 | 1 | (Ellwood et al., 2019) |
|  | OS | 6 | 1 | (Godos et al., 2019) |
| *Phytochemicals* | RCT | 18 | 8 | (Amerizadeh, 2021; Ghaedi, 2020; Han et al., 2019; Ismail, 2021; Li et al., 2020; Noordzij et al., 2005; Onakpoya, Spencer, et al., 2015; Tierney, 2020) |
| *Nitrates* | RCT | 2 | 1 | (He, 2021) |
| *Sweeteners* | RCT | 6 | 1 | (Onakpoya & Heneghan, 2015) |
| *Others* | RCT | 2 | 1 | (Xia, 2020) |

^1^ hypertension, hypertensive people only; OS, observation study; RCT, randomized control trials

**Supplementary Table 4 Summary of randomised control trials by diet exposure**

| **Author, Year (Ref)** | **Comparison** | **Population** | **Regions** | **Study duration** | **Age range (mean)** | **Outcome** | **No. of primary studies/**  **estimates** | **No. of case/ control** |  | **BP Change (95%CI)*** | **I^2^** | **NutriGrade** | **Quality**  **tool** | **AMSTAR** |
| --- | --- | --- | --- | --- | --- | --- | --- | --- | --- | --- | --- | --- | --- | --- |
| ***Diet exposure: Patterns of diet*** | | | | | | | | | | | | | | |
| Baye, 2017 (Baye et al., 2017) | Low AGE products diet vs. high AGE diet or standard (usual) diet | F,M; Healthy, some with Ow, Ob,  DM, O | NA,SA | 8 w to  1 y | 30 to 55 | DBP | 4/4 | 240/  213 | WMCD | 1.80 (-0.80, 4.40) | 0% | Very Low | NR | Moderate |
|  |  |  |  |  |  | SBP | 4/4 | 240/ 213 | WMCD | 1.60 (-2.10, 5.20) | 0% | Very Low |  |  |
| Bonnet, 2020 (Bonnet, 2020) | Breakfast skipping vs.  breakfast consumption | F,M; Healthy, some with Ow | EU,NA | 4 w to  6 w | 34 to 36 (m) | DBP | 2/2 | 69  (total) | WMCD | -2.43 (-5.96, 1.09) | 0% | Very Low | NIH | Critically Low |
|  |  |  |  |  |  | SBP | 2/2 | 69  (total) | WMCD | -6.03 (-14.73, 2.14) | 76% | Very Low |  |  |
| Bueno, 2013 (Bueno et al., 2013) | Very-low-carbohydrate ketogenic diet vs. low fat diet | F,M; Healthy, some with DM | EU,NA,  AU,ME | 12 m to 24 m | 40 to 60 (m) | DBP | 11/11 | 652/  646 | WMCD | -1.43 (-2.49, -0.37)* | 3% | High | Cochrane | High |
|  |  |  |  |  |  | SBP | 11/11 | 652/ 646 | WMCD | -1.47 (-3.44, 0.50) | 33% | Moderate |  |  |
| Chiavaroli, 2018 (Chiavaroli et al., 2018) | Portfolio diet vs. National Cholesterol Education Program (NCEP) Step II diet | F,M; Healthy, some with O | NR | 2 w to 24 w | 55 to 65 (m) | DBP | 5/5 | 439 (total) | MD or MCD | -1.36 (-2.33, -0.38)* | 0% | Low | GRADE | Moderate |
|  |  |  |  |  |  | SBP | 5/7 | 439 (total) | MD or MCD | -1.75 (-3.23, -0.26)* | 0% | Low |  |  |
| Dong, 2020 (Dong, 2020) | Low-carbohydrate diet vs. controls | F,M; Healthy, some with O | NA, AU,  SA, EU | > 6 m | 31 to 65 (m) | DBP | 9/18 | 1420/  1406 | MCD | -1.71 (-2.36, -1.06)* | 14% | Moderate | Cochrane | Low |
|  |  |  |  |  |  | SBP | 10/21 | 1645/  1625 | MCD | -1.41 (-2.26, -0.56)* | 0% | Moderate |  |  |
| Filippou, 2020, (Filippou et al., 2020) | Dietary Approaches to Stop Hypertension (DASH) diet vs. controls | F,M; Healthy, some with HTN | NR | 2 w to 52 w | 31 to 62 (m) | DBP | 30/30 | 2742/  2803 | MCD | -2.50 (-3.50, -1.50)* | 72% | High | GRADE | Low |
|  |  |  |  |  |  | SBP | 30/30 | 2742/  2803 | MCD | -3.20 (-4.20, -2.30)* | 35% | High |  |  |
| Filippou, 2021 (Filippou, 2021) | Mediterranean diet vs. usual diet or other dietary intervention | F,M; Healthy, some with Ob, Ow,  DM, MetS, O | NR | 6 w to  192 w | 23 to 71 (m) | DBP | 35/35 | 8250/  5806 | MCD | -0.90 (-1.50, -0.30)* | 12% | Moderate | GRADE | Low |
|  |  |  |  |  |  | SBP | 35/35 | 8250/  5806 | MCD | -1.50 (-2.80, -0.10)* | 56% | Moderate |  |  |
|  | Mediterranean diet vs. usual diet |  |  | 12 w to 192 w | 23 to 71 (m) | DBP | 15/15 | 1908/  1871 | MCD | -1.60 (-2.60, -0.60)* | 10% | Moderate |  |  |
|  |  |  |  |  |  | SBP | 15/15 | 1908/  1871 | MCD | -3.10 (-4.80, -1.30)* | 33% | Moderate |  |  |
| **Author, Year (Ref)** | **Comparison** | **Population** | **Regions** | **Study duration** | **Age range (mean)** | **Outcome** | **No. of primary studies/**  **estimates** | **No. of case/ control** |  | **BP Change (95%CI)*** | **I^2^** | **NutriGrade** | **Quality**  **tool** | **AMSTAR** |
| Gay, 2016 (Gay et al., 2016) | Dietary interventions vs. controls | F,M; Healthy, some with DM | EU,NA,  AU,SA | 6 m to 48 m | 34 to 67 | DBP | 24/24 | 23858  (total) | WMCD | -1.81 (-2.24, -1.38)* | 86% | Moderate | Cochrane | Moderate |
|  |  |  |  |  |  | SBP | 24/39 | 23858  (total) | WMCD | -3.07 (-3.83, -2.30)* | 89% | Moderate |  |  |
|  | Low calorie/fat diet vs. controls | F,M; Healthy, some with DM | NR | NR | 34 to 67 | DBP | 12/12 | 5090  (total) | WMCD | -1.28 (-1.88, -0.69)* | 60% | Moderate |  |  |
|  |  |  |  |  |  | SBP | 12/13 | 5090  (total) | WMCD | -3.18 (-4.24, -2.11)* | 69% | Moderate |  |  |
|  | Low sodium diet vs. controls | F,M; Healthy, some with DM | NR | NR | 34 to 67 | DBP | 6/6 | 1139  (total) | WMCD | -1.30 (-2.37, -0.23)* | 79% | Moderate |  |  |
|  |  |  |  |  |  | SBP | 6/6 | 1139  (total) | WMCD | -2.06 (-3.50, -0.63)* | 74% | Moderate |  |  |
|  | Low sodium, low calorie/fat diet vs. controls | F,M; Healthy, some with DM | NR | NR | 34 to 67 | DBP | 5/5 | 1509  (total) | WMCD | -1.33 (-2.03, -0.62)* | 37% | Moderate |  |  |
|  |  |  |  |  |  | SBP | 5/5 | 1509  (total) | WMCD | -2.39 (-3.79, -0.98)* | 67% | Moderate |  |  |
|  | Low sodium/high potassium diet vs. controls | F,M; Healthy, some with DM | NR | NR | 34 to 67 | DBP | 4/4 | 370  (total) | WMCD | -2.01 (-3.40, -0.63)* | 66% | Moderate |  |  |
|  |  |  |  |  |  | SBP | 4/5 | 370  (total) | WMCD | -3.14 (-6.27, -0.02)* | 82% | Moderate |  |  |
| Ghaedi, 2019 (Ghaedi et al., 2019) | Paleolithic diet vs. standard (usual) diet | F,M; Healthy, some with DM, MetS | EU,NA,  AU | 14 d to 720 d | 47 to 65 | DBP | 6/6 | 115/  98 | WMCD | -2.95 (-4.72, -1.18)* | 0% | Low | Cochrane | Moderate |
|  |  |  |  |  |  | SBP | 6/6 | 115/  98 | WMCD | -4.24 (-7.11, -1.38)* | 0% | Low |  |  |
| Gibbs, 2021 (Gibbs, 2021) | Plant-based diet vs. controls | F,M; Healthy, some with DM, MetS | EU, NA,  AS, AU, ME | 1.4 w to 208 w | 49.2 (m) | DBP | 21/21 | 1023/  872 | MCD | -2.79 (-4.33, -1.24)* | 88% | Moderate | GRADE | Critically Low |
|  |  |  |  |  |  | SBP | 21/21 | 1023/  872 | MCD | -4.29 (-6.27, -2.31)* | 87% | Moderate |  |  |
| Hu, 2012 (Hu et al., 2012) | Low carb diet vs. low fat diet | F,M; Healthy, some with Ow, Ob, DM, MetS | EU,NA,  AU,ME | 6 m to 24 m | 27 to 60 (m) | DBP | 18/18 | 1201  (total) | MCD | -0.70 (-1.60, 0.20) | 41% | Very Low | NR | Low |
|  |  |  |  |  |  | SBP | 18/18 | 1201  (total) | MCD | -1.00 (-3.50, 1.50) | 92% | Very Low |  |  |
| Kirkham, 2021 (Kirkham, 2021) | Caloric restriction diet vs. controls | F,M; Healthy, some with Ow, Ob, HTN | NR | 12 d to  3 y | 48 to 49 (m) | DBP | 32/44 | 1722 (total) | WMD or WMCD | -2.70 (-3.60, -1.70)* | 76% | Low | SIGN | Critically low |
|  |  |  |  |  |  | SBP | 32/44 | 1700 (total) | WMD or WMCD | -4.50 (-6.30, -2.70)* | 29% | Low |  |  |
| **Author, Year (Ref)** | **Comparison** | **Population** | **Regions** | **Study duration** | **Age range (mean)** | **Outcome** | **No. of primary studies/**  **estimates** | **No. of case/ control** |  | **BP Change (95%CI)*** | **I^2^** | **NutriGrade** | **Quality**  **tool** | **AMSTAR** |
| Lee, 2020 (Lee, 2020) | Vegetarian vs. omnivore | F,M: Healthy, some with DM | NA,SA,AU | 3 w to  24 m | 33 to 64 (m) | DBP | 14/15 | 870 (total) | WMCD | -1.65 (-2.96, -0.35)* | 99% | Moderate | Cochrane  GRADE | Critically low |
|  |  |  |  |  |  | SBP | 14/15 | 870 (total) | WMCD | -2.51 (-3.63, -1.39)* | 98% | Moderate |  |  |
| Lopez, 2019 (Lopez et al., 2019) | Vegan diet vs. standard (usual) diet | F,M; Healthy, some with DM, O | NR | 3 w to 74 w | 33 to 61 (m) | DBP | 11/11 | 983  (total) | WMCD | -1.21 (-3.06, 0.65) | 54% | Moderate | Own designed | Moderate |
|  |  |  |  |  |  | SBP | 11/11 | 983  (total) | WMCD | -1.33 (-3.50, 0.84) | 30% | Moderate |  |  |
| Mazzaro, 2014 (Mazzaro et al., 2014) | Hyperproteic diet vs. controls | F,M; Healthy, some with O | SA | 2 m to  3 m | 23 to 42 (m) | DBP | 3/3 | 37/  33 | WMCD | -3.27 (-13.70, 7.16) | 71% | Moderate | Cochrane | Moderate |
|  |  |  |  |  |  | SBP | 3/3 | 37/  33 | WMCD | 4.95 (-1.25, 11.15) | 0% | Moderate |  |  |
| Naude, 2014 (Naude et al., 2014) | Low carb, high fat diet vs. balanced weight loss diet | F,M; Healthy, some with Ow, Ob | EU,NA,  AU | 3 m to  6 m | 18 to 70 | DBP | 6/6 | 624/  648 | MD or MCD | -0.39 (-1.65, 0.87) | 34% | Moderate | GRADE, Cochrane | Moderate |
|  |  |  |  |  |  | SBP | 5/5 | 477/ 490 | MD or MCD | -1.41 (-2.90, 0.08) | 0% | Moderate |  |  |
|  |  |  |  | 1 y to  2 y | 18 to 70 | DBP | 4/4 | 418/  418 | MD or MCD | 0.01 (-1.67, 1.69) | 30% | Moderate |  |  |
|  |  |  |  |  |  | SBP | 4/4 | 418/ 418 | MD or MCD | -1.38 (-4.07, 1.32) | 35% | Moderate |  |  |
|  | Low carb, high protein diet vs. balanced weight loss diet | F,M; Healthy, some with Ow, Ob | AU | 3 m to  6 m | 20 to 65 | DBP | 2/2 | 44/  46 | MD or MCD | 0.85 (-5.97, 7.68) | 77% | Moderate |  |  |
|  |  |  |  |  |  | SBP | 2/2 | 44/  46 | MD or MCD | -0.79 (-7.32, 5.74) | 27% | Moderate |  |  |
|  |  |  |  | 1 y to  2 y | 20 to 65 | DBP | 2/2 | 38/  40 | MD or MCD | -1.21 (-8.89, 6.48) | 64% | Moderate |  |  |
|  |  |  |  |  |  | SBP | 2/2 | 38/  40 | MD or MCD | -6.54 (-21.01, 8.10) | 77% | Moderate |  |  |
| Ramezani-Jolfaie, 2019 (Ramezani-Jolfaie et al., 2019) | Nordic diet vs. standard (usual) diet | F,M; Healthy, some with Ob, MetS,  O | EU | 42 d to 182 d | 41 to 59 (m) | DBP | 4/4 | 192  (total) | WMCD | -2.08 (-3.44, -0.73)* | 0% | Low | Cochrane | High |
|  |  |  |  |  |  | SBP | 4/4 | 492  (total) | WMCD | -3.97 (-6.40, -1.54)* | 26% | Low |  |  |
| Santesso, 2012 (Santesso et al., 2012) | High protein diet vs. low protein diet | F,M; Healthy, some with Ow, Ob,  HTN | EU,NA,  AU | 28 d to 180 d | 26 to 54 (m) | DBP | 15/15 | 587/ 599 | MCD or SMD | -0.18 (-0.29, -0.06)* | 2% | Moderate | GRADE | High |
|  |  |  |  |  |  | SBP | 15/15 | 587/ 599 | MCD or SMD | -0.21 (-0.32, -0.09)* | 0% | Moderate |  |  |
| **Author, Year (Ref)** | **Comparison** | **Population** | **Regions** | **Study duration** | **Age range (mean)** | **Outcome** | **No. of primary studies/**  **estimates** | **No. of case/ control** |  | **BP Change (95%CI)*** | **I^2^** | **NutriGrade** | **Quality**  **tool** | **AMSTAR** |
| Schwingshackl, 2011 (Schwingshackl et al., 2011) | High monounsaturated fatty acids diet vs. high glycemic index or low glycemic index or high protein or high polyunsaturated fatty acids or low total fat diets | NR; Healthy, some with Ob, DM,  HTN | NR | 6 m to  4 y | From 18 | DBP | 9/9 | 801/ 779 | WMCD | -1.15 (-1.96, -0.34)* | 3% | Moderate | JADAD | Moderate |
|  |  |  |  |  |  | SBP | 9/11 | 790/ 780 | WMCD | -2.26 (-4.28, -0.25)* | 50% | Moderate |  |  |
| Schwingshackl, 2013 (Schwingshackl & Hoffmann, 2013) | Low fat, high protein diet vs. low fat, low protein diet | F,M; Healthy, some with DM | NR | >12 m | From 18 | DBP | 11/11 | 1402  (total) | WMD or WMCD | -0.42 (-1.37, 0.54) | 0% | Moderate | JADAD, Cochrane | Moderate |
|  |  |  |  |  |  | SBP | 11/11 | 1414  (total) | WMD or WMCD | -1.61 (-3.45, 0.23) | 41% | Low |  |  |
| Shah, 2007 (Shah et al., 2007) | High carb diet vs. high cis-MUFA | F,M; Healthy, some with DM, HTN, O | NR | 3 w to 14 w | From 18 | DBP | 7/7 | 328  (total) | WMCD | 0.90 (-0.10, 1.90) | Sig | Low | NR | Critically Low |
|  |  |  |  |  |  | SBP | 7/7 | 328  (total) | WMCD | 1.30 (-0.10, 2.60) | Sig | Low |  |  |
| Toh, 2020 (Toh, 2020) | High fruit and vegetable diet vs. low fruit and vegetable diet | F,M; Healthy, some with MetS | NR | 4 w to  2 y | 44 to 49 (m) | DBP | 3/4 | 300 (total) | WMCD | -1.99 (-2.28, -1.70)* | 0% | Very low | Cochrane | Critically Low |
|  |  |  |  |  |  | SBP | 3/4 | 300 (total) | WMCD | 0.72 (-3.39, 4.83) | 76% | Very low |  |  |
| Yang, 2021 (Yang, 2021) | Low fat, high carbohydrate diet vs.  Low carbohydrate, high fat diet | F,M; Healthy | NA,AU | 8 w to  52 w | 37 to 55 (m) | DBP | 6/6 | 191/  198 | SMD | -0.37 (-1.45, 0.71) | 96% | Moderate | Cochrane | Critically Low |
|  |  |  |  |  |  | SBP | 6/6 | 191/  198 | SMD | -0.44 (-1.28, 0.40) | 93% | Moderate |  |  |
| ***Diet exposure: Meat, poultry, egg, and fish*** | | | | | | | | | | | | | | |
| Alhassan, 2017 (Alhassan et al., 2017) | Fish vs. no fish diet, sunflower oil capsules | F,M; Healthy, some with Ow, Ob, MetS, O | EU,NA,  AS,AU | 4 w to 24 w | 23 to 70 (m) | DBP | 5/5 | 308/ 281 | WMCD | -1.06 (-2.61, 0.48) | 11% | Moderate | JADAD | Moderate |
|  |  |  |  |  |  | SBP | 5/5 | 308/ 281 | WMCD | -1.51 (-4.08, 1.06) | 21% | Moderate |  |  |
| O'Connor, 2017 (O'Connor et al., 2017) | Total red meat vs. no red meat with fish, soy, chicken | F,M; Healthy, some with HTN,O | NR | 6 w to 14 w | 33 to 70 (m) | DBP | 6/6 | 323  (total) | WMCD | 0.10 (-1.20, 1.50) | 49% | Very Low | Cochrane | Moderate |
|  |  |  |  |  |  | SBP | 6/6 | 323  (total) | WMCD | -1.00 (-2.40, 0.80) | 11% | Very Low |  |  |
| Kolahdouz-Mohammadi, 2020  (Kolahdouz-Mohammadi, 2020) | Egg vs. controls | F,M; Healthy, some with DM, MetS,O | NA,SA,  AS, AU | 3 w to 12 w | 23 to 67 (m) | DBP | 15/15 | 748  (total) | WMCD | -0.60 (-1.52, 0.31) | 38% | Moderate | Cochrane, NutriGrade | Critically Low |
|  |  |  |  |  |  | SBP | 15/15 | 748  (total) | WMCD | 0.05 (-0.79, 0.88) | 0% | Moderate |  |  |
| **Author, Year (Ref)** | **Comparison** | **Population** | **Regions** | **Study duration** | **Age range (mean)** | **Outcome** | **No. of primary studies/**  **estimates** | **No. of case/ control** |  | **BP Change (95%CI)*** | **I^2^** | **NutriGrade** | **Quality**  **tool** | **AMSTAR** |
| ***Diet exposure: Milk and dairy*** | | | | | | | | | | | | | | |
| Benatar, 2013 (Benatar et al., 2013) | Whole fat dairy products vs. standard (usual) diet | F,M; Healthy, some with Ow, Ob, MetS, O | EU,NA,  AU | 4 w to 26 w | 20 to 51 (m) | DBP | 4/4 | 179/ 190 | WMCD | -0.69 (-3.20, 1.83) | NR | Low | JADAD | Moderate |
|  |  |  |  |  |  | SBP | 4/4 | 179/ 190 | WMCD | 0.07 (-1.69, 1.83) | NR | Low |  |  |
|  | Low fat dairy vs. standard (usual) diet | F,M; Healthy, some with Ow, Ob,  MetS, O | EU,NA,  AU | 8 w to 26 w | 47 to 65 (m) | DBP | 3/3 | 169/ 173 | WMCD | -0.43 (-1.68, 0.82) | NR | Low | JADAD |  |
|  |  |  |  |  |  | SBP | 3/3 | 169/ 173 | WMCD | -0.85 (-2.55, 0.84) | NR | Low |  |  |
| Fekete, 2015 (Fekete et al., 2015) | Lactotripeptides vs. controls | F,M; Healthy, some with HTN | EU,NA,  AS | 2 w to 21 w | 30 to 75 (m) | DBP | 30/30 | 2200  (total) | WMCD | -1.51 (-2.21, -0.80)* | 49% | Moderate | PRISMA, Cochrane | Moderate |
|  |  |  |  |  |  | SBP | 30/33 | 2200  (total) | WMCD | -2.95 (-4.17, -1.73)* | 77% | Moderate |  |  |
| ***Diet exposure: Fruits and vegetables*** | | | | | | | | | | | | | | |
| Carvalho, 2021  (Carvalho, 2021) | Blueberry vs. controls | F,M; Health, some with O, MetS | NR | 1 w to  24 w | 40 to 73 (m) | DBP | 8/8 | 147/  149 | MCD | -2.02 (-3.84, -0.19)* | 12% | Moderate | GRADE | Low |
|  |  |  |  |  |  | SBP | 8/8 | 147/  149 | MCD | -2.65 (-5.76, 0.46) | 0% | Moderate |  |  |
| D’Elia, 2021 (D'Elia, 2021) | 100% fruit juice vs. controls | F,M; Healthy,  Some with Ow, MetS, HTN, O | EU,NA,  AS, ME,  SA | 1 w to  16 w | 21 to  78 (m) | DBP | 25/26 | 1320  (total) | WMD | -1.68 (-2.94, -0.43)* | 31% | Very low | Cochrane, GRADE | Low |
|  |  |  |  |  |  | SBP | 25/26 | 1320  (total) | WMD | -3.14 (-4.43, -1.85)* | 0% | Very low |  |  |
| Guo, 2017 (Guo et al., 2017) | Goji berry vs. controls | F,M; Healthy, some with DM | NA,AS | 14 d to  3 m | 31 to 67 (m) | DBP | 6/6 | 243/  242 | WMCD | -1.59 (-4.57, 1.40) | 64% | Low | JADAD | Moderate |
|  |  |  |  |  |  | SBP | 6/7 | 243/ 242 | WMCD | 0.02 (-4.26, 4.30) | 54% | Low |  |  |
| Hawkins, 2021  (Hawkins et al., 2021) | Aronia berry vs. controls | NR; Health, some with HTN, O | EU, NA | 6 w to  8 w | 18 to 66 (m) | DBP | 4/4 | 149/  141 | SMD | 0.08 (-0.27, 0.42) | 56% | Very low | Downs and Black | Critically low |
|  |  |  |  |  |  | SBP | 4/4 | 149/  141 | SMD | -0.33 (-0.56, -0.10)* | 8% | Very low |  |  |
| Hadi, 2019 (Hadi et al., 2019) | Strawberry vs. controls | F,M; Healthy, some with DM,O | NA,ME | 4 w to 12 w | 47 to 62 (m) | DBP | 11/11 | 312  (total) | WMCD | -2.22 (-4.26, -0.18)* | 66% | Low | Cochrane | Low |
|  |  |  |  |  |  | SBP | 11/11 | 312  (total) | WMCD | -0.75 (-2.58, 1.09) | 0% | Moderate |  |  |
| Han, 2020  (Han, 2020) | Sour cherry vs. controls | F,M; Healthy, | NA, EU | 6 w to 12 w | 30 to 80 | DBP | 3/3 | 102  (total) | WMD | -2.32 (-4.45, -0.19)* | 39% | Low | Cochrane | Critically low |
|  |  |  |  |  |  | SBP | 3/3 | 102  (total) | WMD | -2.64 (-5.84, 0.56) | 33% | Low |  |  |
| **Author, Year (Ref)** | **Comparison** | **Population** | **Regions** | **Study duration** | **Age range (mean)** | **Outcome** | **No. of primary studies/**  **estimates** | **No. of case/ control** |  | **BP Change (95%CI)*** | **I^2^** | **NutriGrade** | **Quality**  **tool** | **AMSTAR** |
| Liu, 2013 (Liu et al., 2013) | Fruit juice vs. controls | F,M; Healthy, some with Ow, DM,  HTN, O | EU,NA,  ME | 4 w to  3 m | From 18 | DBP | 7/7 | 267  (total) | WMD | -2.07 (-3.75, -0.39)* | 46% | Low | JADAD | Moderate |
|  |  |  |  |  |  | SBP | 7/8 | 267  (total) | WMD | -2.03 (-4.47, 0.41) | 0% | Low |  |  |
| Luis, 2018 (Luis et al., 2018b) | Berries vs. controls | F,M; Healthy, some with Ow, MetS, HTN, O | EU,NA,  AS | 8 w to 24 w | From 18 | DBP | 34/34 | 1638  (total) | WMCD | -1.43 (-2.48, -0.39)* | 40% | Moderate | Cochrane | Moderate |
|  |  |  |  |  |  | SBP | 33/33 | 1600  (total) | WMCD | -2.07 (-3.50, -0.64)* | 17% | Moderate |  |  |
| Motallaei, 2021 (Motallaei, 2021) | Orange juice vs. controls | F,M; Healthy, some with Ob, HTN | AS, SA, EU, ME | 2 w to  12 w | From 18 | DBP | 5/5 | 116/  115 | WMCD | 0.61 (-3.82, 5.05) | 76% | Low | Cochrane | Low |
|  |  |  |  |  |  | SBP | 6/6 | 165/  164 | WMCD | -0.22 (-3.92, 3.47) | 71% | Low |  |  |
| Onakpoya, 2015 (Onakpoya, O'Sullivan, et al., 2015) | Cactus pear vs. controls | F,M; Healthy, some with Ow, Ob, MetS | EU,SA | 8 w to 12 w | 20 to 60 | DBP | 2/2 | 190  (total) | MD | -1.14 (-1.61, -0.67)* | 0% | Moderate | Cochrane | Moderate |
|  |  |  |  |  |  | SBP | 2/2 | 190  (total) | MD | -0.88 (-1.76, -0.01)* | 0% | Moderate |  |  |
| Onakpoya, 2017 (Onakpoya et al., 2017) | Grapefruit vs. controls | F,M; Healthy, some with  Ow | NR | 6 w to 12 w | From 18 | DBP | 3/3 | 233  (total) | MD | -1.65 (-3.92, 0.63) | 0% | Low | GRADE | Moderate |
|  |  |  |  |  |  | SBP | 3/3 | 155/  78 | MD | -2.43 (-4.77, -0.09)* | 0% | Low |  |  |
| Pourmasoumi, 2020 (Pourmasoumi et al., 2020) | Cranberry vs. controls | F,M; Healthy, some with Ow, DM, MetS, O | EU,NA,  ME | 28 d to 56 d | 22 to 62 (m) | DBP | 6/6 | 174/ 162 | WMCD | -2.03 (-5.02, 0.96) | 62% | Low | Cochrane | Moderate |
|  |  |  |  |  |  | SBP | 6/7 | 159/ 147 | WMCD | -3.63 (-6.27, -0.98)* | 32% | Low |  |  |
| Sahebkar, 2017 (Sahebkar et al., 2017) | Pomegranate vs. controls | F,M; Healthy, some with HTN,O | NR | 4 w to 18 m | 36 to 69 (m) | DBP | 8/8 | 333/ 290 | WMCD | -2.01 (-3.72, -0.31)* | NR | Moderate | Cochrane | Critically Low |
|  |  |  |  |  |  | SBP | 8/8 | 333/ 290 | WMCD | -4.96 (-7.67, -2.23)* | NR | Moderate |  |  |
| Suksomboon, 2019 (Suksomboon et al., 2019) | Kiwi fruit vs. controls | F,M; Healthy, some with HTN,O | EU,AS  ,AU | 4 w to  8 w | 30 to 75 | DBP | 4/4 | 162/ 165 | WMCD | -2.35 (-5.10, 0.41) | 0% | Low | Cochrane | Moderate |
|  |  |  |  |  |  | SBP | 4/4 | 162/ 165 | WMCD | -1.72 (-4.27, 0.84) | 37% | Low |  |  |
| Bahadoran, 2017 (Bahadoran et al., 2017) | Beetroot vs. controls | F,M; Healthy, some with Ow, DM,  HTN,O | NR | 2 d to  56 d | 21 to 69 (m) | DBP | 19/19 | 650/ 598 | WMCD | -1.32 (-1.97, -0.68)* | 0% | Moderate | JADAD | Moderate |
|  |  |  |  |  |  | SBP | 19/43 | 650/ 598 | WMCD | -3.55 (-4.55, -2.54)* | 30% | Moderate |  |  |
| **Author, Year (Ref)** | **Comparison** | **Population** | **Regions** | **Study duration** | **Age range (mean)** | **Outcome** | **No. of primary studies/**  **estimates** | **No. of case/ control** |  | **BP Change (95%CI)*** | **I^2^** | **NutriGrade** | **Quality**  **tool** | **AMSTAR** |
| Hasani, 2019 (Hasani et al., 2019) | Ginger vs. controls | F,M; Healthy, some with Ow,DM,O | ME | 7 w to 12 w | 22 to 54 (m) | DBP | 6/6 | 175/  170 | MCD | -2.12 (-3.92, -0.31)* | 73% | Low | JADAD | Low |
|  |  |  |  |  |  | SBP | 6/6 | 175/ 170 | MCD | -6.36 (-11.27, -1.46)* | 90% | Low |  |  |
| Jandari, 2020  (Jandari, 2020) | Bitter melon vs. controls | F,M; Healthy, some with DM | AF,AS,  SA | 4 w to 16 w | 47 to 57 (m) | DBP | 5/6 | 154/  151 | WMD | -0.80 (-2.65, 1.04) | 38% | Moderate | Cochrane | Low |
|  |  |  |  |  |  | SBP | 5/6 | 154/  151 | WMD | -2.28 (-6.62, 2.05) | 69% | Moderate |  |  |
| Ried, 2016 (Ried, 2016) | Garlic vs. controls | NR; Healthy, some with HTN | NR | 2 w to 24 w | From 18 | DBP | 20/20 | 485/ 487 | WMCD | -2.48 (-4.07, -0.89)* | 72% | Low | NR | Critically Low |
|  |  |  |  |  |  | SBP | 19/25 | 453/ 455 | WMCD | -5.07 (-7.30, -2.85)* | 71% | Low |  |  |
|  |  |  |  |  |  | SBP | 5/5 | 143/ 136 | **SMD** | **-0.03 (-0.80, 0.74)** | 86% | Low |  |  |
| Wang, 2020 (Wang, 2020) | Tomato vs. controls | F,M; Healthy | EU,AS | 4 w to 12 w | From 18 | DBP | 5/7 | 294/  294 | WMCD | -0.49 (-1.52, 0.55) | 0% | Low | Cochrane | Critically Low |
|  |  |  |  |  |  | SBP | 5/7 | 294/  294 | WMCD | -1.68 (-7.33, 3.97) | 89% | Low |  |  |
| Shin, 2015 (Shin et al., 2015) | Fruits and vegetable vs. controls | NR; Healthy, some with MetS | EU,NA,  ME | 6 w to 20 w | From 18 | DBP | 5/5 | 143/ 136 | **SMD** | **-0.29 (-0.57, -0.02)*** | 0% | Low | Nordic Cochrane | Moderate |
| ***Diet exposure: Starchy foods*** | | | | | | | | | | | | | | |
| Kelly, 2017 (Kelly et al., 2017) | Whole grains vs. low whole grains | F,M; Healthy, some with Ob, DM, HTN, O | EU,NA,  AS | 12 w to 16 w | From 18 | DBP | 7/7 | 424/  344 | MCD | 0.16 (-0.89, 1.21) | 0% | Low | GRADE, Cochrane | Moderate |
| Reynolds, 2019 (Reynolds et al., 2019) | Whole grains vs. low whole grains | F,M; Healthy, some with Ow | EU,NA | 4 w to 12 w | From 18 | SBP | 8/8 | 493/ 432 | **SMD** | -1.01 (-2.46, 0.44) | NR | Low | GRADE, Cochrane | Moderate |
| ***Diet exposure: Legumes and pulses*** | | | | | | | | | | | | | | |
| Jayalath, 2014 (Jayalath, de Souza, et al., 2014) | Pulses vs. standard (usual) diet | F,M; Healthy, some with Ow, Ob,DM, MetS | NR | 29 d to  1 y | 28 to 60 (m) | DBP | 8/8 | 554  (total) | WMCD | -0.71 (-1.74, 0.31) | 58% | Moderate | Heyland MQS, Cochrane | High |
|  |  |  |  |  |  | SBP | 8/8 | 554  (total) | WMCD | -2.25 (-4.22, -0.28)* | 73% | Moderate |  |  |
| Mohammadifard, 2015 (Mohammadifard et al., 2015) | Peanuts vs. standard (usual) diet | F,M; Healthy, some with O | EU,ME | 2 w to  4 w | 23 to 54 (m) | DBP | 2/2 | 79  (total) | WMCD | -1.62 (-4.47, 1.22) | 0% | Low | Cochrane | Moderate |
|  |  |  |  |  |  | SBP | 2/2 | 79  (total) | WMCD | 2.51 (-1.01, 6.03) | 0% | Low |  |  |
|  | Soy nuts vs. controls | F; Healthy, some with MetS | ME | 4 w to 12 w | From 18 | DBP | 2/2 | 92  (total) | WMCD | 0.17 (-2.53, 2.86) | 52% | Low |  |  |
|  |  |  |  |  |  | SBP | 2/2 | 92  (total) | WMCD | -1.19 (-4.26, 1.88) | 67% | Low |  |  |
| **Author, Year (Ref)** | **Comparison** | **Population** | **Regions** | **Study duration** | **Age range (mean)** | **Outcome** | **No. of primary studies/**  **estimates** | **No. of case/ control** |  | **BP Change (95%CI)*** | **I^2^** | **NutriGrade** | **Quality**  **tool** | **AMSTAR** |
| ***Diet exposure: Nuts and seeds*** | | | | | | | | | | | | | | |
| Asbaghi, 2021 (Asbaghi, Hadi, et al., 2021) | Pistachios vs. controls | F,M; Healthy, some with  DM,Ow | NA,EU,  ME | 4 w to  16 w | 22 to 60 (m) | DBP | 6/7 | 208/  208 | MCD | 0.33 (-1.38, 2.03) | 65% | Low | Cochrane | Moderate |
|  |  |  |  |  |  | SBP | 6/7 | 208/  208 | MCD | -2.12 (-3.65, -0.59)* | 30% | Low |  |  |
| Blanco Mejia, 2014 (Blanco Mejia et al., 2014) | Mixed nuts vs. standard (usual) diet | F,M; Healthy, some with DM, MetS | EU,NA | 12 w to 12 w | 51 to 63 (m) | DBP | 2/2 | 129  (total) | MCD | -0.21 (-3.23, 2.81) | NR | Low | Heyland MQS, Cochrane | Moderate |
|  | Tree nuts vs. standard (usual) diet | F,M; Healthy, some with DM, MetS,O | EU,NA,  AS,AF,  AU,ME | 4 w to 18 m | 24 to 66 (m) | DBP | 20/20 | 835/  670 | MCD | 0.23 (-0.38, 0.83) | 34% | Low |  |  |
|  |  |  |  |  |  | SBP | 20/20 | 835/ 670 | MCD | 0.07 (-1.54, 1.68) | 64% | Very low |  |  |
| Eslampour, 2020 (Eslampour, 2020) | Almonds vs. controls | F,M; Healthy, some with DM, MetS, Ow, Ob, Q | EU,NA, AS,AU,  ME,SA | 3 w to 77 w | From 18 | DBP | 16/23 | 685/  443 | WMCD | -1.30 (-2.31, -0.30)* | 0% | Low | Cochrane | Low |
|  |  |  |  |  |  | SBP | 16/23 | 685/  443 | WMCD | -0.83 (-2.55, 0.89) | 59% | Low |  |  |
| Huang, 2021  (Huang, 2021) | Sesame vs. controls | F,M; Healthy, some with Ow, Ob, DM, HTN, O | AS,AU,  ME | 4 w to  8 w | From 18 | DBP | 10/10 | 895  (total) | WMD | -5.14 (-8.48, -1.80)* | 95% | Moderate | GRADE | Low |
|  |  |  |  |  |  | SBP | 10/10 | 895  (total) | WMD | -5.26 (-9.28, -1.44)* | 92% | Moderate |  |  |
| Jalali, 2020 (Jalali, 2020) | Cashew nuts vs. controls | F,M; Healthy, some with DM, MetS | AF, AS | 8 w to 12 w | 45 to 51 (m) | DBP | 2/2 | 150/  162 | WMCD | -1.45 (-3.16, 0.26) | 0% | Low | Cochrane | Low |
|  |  |  |  |  |  | SBP | 2/2 | 150/  162 | WMCD | -3.39 (-6.13, -0.65)* | 0% | Low |  |  |
| Li, 2020 (Li, 2020) | Walnuts vs. controls | F,M; Healthy, some with Ow, Ob, DM, MetS, O | EU,NA,  ME,AS, AU | 4 w to  6 m | From 18 | DBP | 17/18 | 917/  882 | WMCD | 0.08 (-0.26, 0.42) | 35% | Moderate | AHRQ | Critically Low |
|  |  |  |  |  |  | SBP | 17/18 | 917/  882 | WMCD | 0.08 (-0.69, 0.85) | 85% | Moderate |  |  |
| Mohammadifard, 2015 (Mohammadifard et al., 2015) | Mixed nuts vs. controls | F,M; Healthy, some with DM, MetS | EU,NA | 12 w to NR | 52 to 69 (m) | SBP | 3/4 | 682 | WMCD | -0.73 (-5.16, 3.69) | 79% | Low | Cochrane | Moderate |
| Sahebkar, 2016 (Sahebkar et al., 2016) | Nigella seeds vs. controls | F,M; Healthy, some with DM,HTN,O | NR | 4 w to 12 w | 39 to 56 (m) | DBP | 10/10 | 370/  361 | WMCD | -2.80 (-4.28, -1.32)* | 60% | Moderate | Cochrane | Moderate |
|  |  |  |  |  |  | SBP | 11/11 | 413/ 402 | WMCD | -3.26 (-5.10, -1.42)* | 59% | Moderate |  |  |
| **Author, Year (Ref)** | **Comparison** | **Population** | **Regions** | **Study duration** | **Age range (mean)** | **Outcome** | **No. of primary studies/**  **estimates** | **No. of case/ control** |  | **BP Change (95%CI)*** | **I^2^** | **NutriGrade** | **Quality**  **tool** | **AMSTAR** |
| Teoh, 2018 (Teoh et al., 2018) | Chia seed vs. controls | F,M; Healthy, some with Ow, Ob, DM,MetS | NA,SA | 60 d to 168 d | 18 to 75 | DBP | 4/4 | 231/  171 | MCD | -3.37 (-7.43, 0.70) | 65% | Low | GRADE | Moderate |
|  |  |  |  |  |  | SBP | 6/6 | 383/ 303 | MCD | -2.57 (-6.70, 1.55) | 38% | Very Low |  |  |
| Ursoniu, 2016 (Ursoniu et al., 2016) | Flaxseed vs. controls | F,M; Healthy, some with DM,HTN | EU,NA,  AS,AU,  SA | 4 w to 12 m | From 18 | DBP | 15/15 | 618/  554 | WMCD | -2.39 (-3.78, -0.99)* | NR | High | Cochrane | Low |
|  |  |  |  |  |  | SBP | 15/19 | 618/ 554 | WMCD | -2.85 (-5.37, -0.33)* | NR | High |  |  |
| ***Diet exposure: Cocoa*** | | | | | | | | | | | | | | |
| Ried, 2017 (Ried et al., 2017) | Cocoa products vs. controls | F,M; Healthy, some with Ow,HTN,O | EU,NA,  AS,AU,  ME,SA | 2 w to 18 w | From 18 | DBP | 34/34 | 891/  881 | MCD | -1.76 (-2.57, -0.94)* | 78% | Moderate | GRADE | High |
|  |  |  |  |  |  | SBP | 35/40 | 907/ 897 | MCD | -1.76 (-3.09, -0.43)* | 87% | Moderate |  |  |
| ***Diet exposure: Herb, spice, and condiment*** | | | | | | | | | | | | | | |
| Ashtary-Larky, 2021 (Ashtary-Larky, 2021) | Curcumin vs. controls | F,M; Healthy,  Some with MetS, O | ME | 12 w | 42 to 56 (m) | DBP | 3/3 | 170  (total) | WMCD | -0.07 (-1.12, 0.97) | 0% | Low | GRADE | Low |
|  |  |  |  | 6 w to  12 w | 42 to 62 (m) | SBP | 4/5 | 203  (total) | WMCD | -7.09 (-12.98, -1.20)* | 83% | Low |  |  |
| Luis, 2018 (Luis et al., 2018b) | Licorice vs. controls | F,M; Healthy, some with HTN | AS | 6 w to  8 w | 23 to 50 (m) | DBP | 6/6 | 295/  295 | WMCD | 1.74 (0.84, 2.62)* | 96% | Moderate | Cochrane | Moderate |
|  |  |  |  |  |  | SBP | 6/14 | 295/ 295 | WMCD | 0.78 (-0.41, 1.97) | 93% | Moderate |  |  |
| Mousavi, 2019 (Mousavi et al., 2019) | Cinnamon vs. controls | F,M; Healthy, some with DM,MetS,  HTN | EU,NA,  AS,ME | 8 w to 16 w | 44 to 62 (m) | DBP | 9/9 | 318/  323 | WMCD | -3.93 (-6.33, -1.53)* | 96% | Moderate | Cochrane | Moderate |
|  |  |  |  |  |  | SBP | 9/9 | 318/ 323 | WMCD | -6.24 (-10.70, -1.77)* | 98% | Moderate |  |  |
| Setayesh, 2021  (Setayesh, 2021) | Saffron vs. controls | F,M; Healthy, some with DM, MetS,  O | ME | 1 w to 12 w | From 18 | DBP | 7/8 | 209/  195 | WMCD | -1.23 (-1.64, -0.81)* | 0% | Very low | Cochrane | Critically Low |
|  |  |  |  |  |  | SBP | 8/10 | 249/  237 | WMCD | -0.65 (-1.12 , -0.18)* | 47% | Very low |  |  |
| ***Diet exposure: Beverages*** | | | | | | | | | | | | | | |
| Roerecke, 2017 (Roerecke et al., 2017) | Alcohol reduction vs. alcohol | F,M; Healthy, some with HTN | EU,NA,  AS,AU | 1 w to 104 w | 49.5 (m) | DBP | 32/36 | 2865  (total) | WMCD | -2.00 (-2.65, -1.35)* | 80% | Moderate | GRADE, Cochrane | Moderate |
|  |  |  |  |  |  | SBP | 34/40 | 2865  (total) | WMCD | -3.13 (-3.93, -2.32)* | 82% | Moderate |  |  |
| Spaggiari, 2020 (Spaggiari, 2020) | Beer vs. controls | F,M; Healthy, some with Ow, HTN | NR | 3 w to 18 w | 21 to 61 (m) | DBP | 10/10 | 363/  347 | WMD | 0.15 (-1.07, 1.38) | 98% | Low | Cochrane | Critically Low |
|  |  |  |  |  |  | SBP | 10/10 | 363/  347 | WMD | 0.74 (-0.76, 2.24) | 97% | Low |  |  |
| **Author, Year (Ref)** | **Comparison** | **Population** | **Regions** | **Study duration** | **Age range (mean)** | **Outcome** | **No. of primary studies/**  **estimates** | **No. of case/ control** |  | **BP Change (95%CI)*** | **I^2^** | **NutriGrade** | **Quality**  **tool** | **AMSTAR** |
| Ramli 2021(Ramli, 2021) | Decaffeinated coffee vs. controls | F,M; Healthy, | SA, AS, ME,EU | 1 h to  12 w | From 18 | DBP | 3/4 | 268  (total) | WMCD | -0.49 (-0.93, -0.05)* | 97% | Low | JADAD | Low |
|  |  |  |  |  |  | SBP | 4/7 | 305  (total) | WMCD | -0.22 (-0.43, -0.21)* | 95% | Low |  |  |
| Steffen, 2012 (Steffen et al., 2012) | Coffee vs. controls | F,M; Healthy, some with HTN | NR | 4 w to 16 w | 25 to 73 (m) | DBP | 10/10 | 737/  730 | WMCD | -0.45 (-1.52, 0.61) | 41% | Moderate | Cochrane | Moderate |
|  |  |  |  |  |  | SBP | 10/12 | 737/ 730 | WMCD | -0.55 (-2.46, 1.36) | 72% | Moderate |  |  |
| Liu, 2014 (Liu et al., 2014) | Tea vs. controls | F,M; Healthy, some with HTN, O | EU,NA,  AU | 1 h to  24 h | 24 to 62 (m) | DBP | 6/6 | 203  (total) | WMCD | 0.71 (-0.01, 1.42) | 7% | Low | JADAD | Moderate |
|  |  |  |  |  |  | SBP | 6/6 | 203  (total) | WMCD | 2.42 (-0.62, 5.46) | 70% | Low |  |  |
| Ma, 2021 (Ma, 2021) | Black tea vs. controls | F,M; Healthy, some with HTN, O | EU,NA,  AS,AU | 1 h to  6 m | 22 to  66 (m) | DBP | 11/11 | 1087  (total) | WMCD | -0.59 (-1.05, -0.13)* | 71% | Moderate | Cochrane | Moderate |
|  |  |  |  |  |  | SBP | 13/22 | 556/  559 | WMCD | -1.04 (-2.05, -0.03)* | 71% | Moderate |  |  |
| Serban, 2015 (Serban et al., 2015) | Sour tea (hibiscus) vs. controls | F,M; Healthy, some with MetS | NA,ME,  SA | 15 d to  6 w | From 18 | DBP | 5/5 | 205/  165 | WMCD | -3.53 (-5.16, -1.89)* | 68% | Low | JADAD | Moderate |
|  |  |  |  |  |  | SBP | 5/7 | 205/ 165 | WMCD | -7.58 (-9.69, -5.46)* | 92% | Low |  |  |
| Xu, 2020 (Xu, 2020) | Green tea vs. controls | F,M; Healthy, some with Ow, Ob,DM,  HTN | EU,NA,  AS,ME,  SA | 3 w to  16 w | 22 to 74 (m) | DBP | 24/25 | 1697  (total) | WMCD | -1.24 (-2.07, -0.40)* | 57% | Moderate | JADAD | Critically Low |
|  |  |  |  |  |  | SBP | 24/25 | 1697  (total) | WMCD | -1.17 (-2.18, -0.16)* | 43% | Moderate |  |  |
| ***Diet exposure: Carbohydrates*** | | | | | | | | | | | | | | |
| Clark, 2020 (Clark, 2020) | Psyllium vs. controls | F,M; Healthy, some with HTN,DM,O | EU,ME,  NA,AU | 4 w to  6 m | 25 to 70 | DBP | 11/15 | 300/  292 | WMCD | -0.72 (-1.98, -0.53)* | 32% | Moderate | JADAD | Moderate |
|  |  |  |  |  |  | SBP | 11/15 | 300/  292 | WMCD | -2.22 (-2.82, -1.63)* | 0% | Moderate |  |  |
| Evans, 2015 (Evans et al., 2015) | Arabinoxylan-rich foods vs. controls | F,M; Healthy, some with Ow,Ob,HTN | EU,NA | 6 w to 14 w | 48 to 60 (m) | DBP | 3/3 | 137  (total) | WMCD | -0.73 (-3.66, 2.20) | 0% | Low | Own designed | Low |
|  |  |  |  |  |  | SBP | 3/3 | 137  (total) | WMCD | -0.09 (-4.61, 4.44) | 0% | Low |  |  |
|  | Mannans vs. controls | F,M; Healthy, some with Ow,Ob,HTN | EU,NA,  AS | 6 w to 14 w | 39 to 52 (m) | DBP | 3/3 | 119  (total) | WMCD | 1.66 (-4.26, 7.58) | 91% | Low |  |  |
|  |  |  |  |  |  | SBP | 3/3 | 119  (total) | WMCD | 0.36 (-4.29, 5.01) | 81% | Low |  |  |
| **Author, Year (Ref)** | **Comparison** | **Population** | **Regions** | **Study duration** | **Age range (mean)** | **Outcome** | **No. of primary studies/**  **estimates** | **No. of case/ control** |  | **BP Change (95%CI)*** | **I^2^** | **NutriGrade** | **Quality**  **tool** | **AMSTAR** |
| Faghihimani, 2021 (Faghihimani, 2021) | Inulin type carbohydrates vs. controls | F,M; Healthy, some with DM, Ob, O | EU,SA,  ME | 3 w to  8.5 w | 40 to 51 (m) | DBP | 5/6 | 121/  112 | WMCD | -5.83 (-12.49, 0.82) | 59% | Moderate | Cochrane | Low |
|  |  |  |  |  |  | SBP | 5/6 | 121/  112 | WMCD | -2.62 (-6.15, 0.92) | 51% | Moderate |  |  |
| Hashemi Tari, 2021 (Hashemi Tari, 2021) | Inositol vs. controls | F,M; Healthy, some with DM, O | EU,SA, AS | 6 w to  1 y | 28 to 61 (m) | DBP | 7/7 | 168/  154 | WMCD | -7.12 (-10.18, -4.05)* | 0% | Moderate | Cochrane | Critically Low |
|  |  |  |  |  |  | SBP | 7/7 | 168/  154 | WMCD | -5.69 (-7.35 , -4.02)* | 0% | Moderate |  |  |
| Khan, 2018 (Khan et al., 2018) | B-glucan vs. controls | F,M; Healthy, some with OW,DM,HTN | EU,NA  ,AS | 4 w to 24 w | 18 to 70 | DBP | 7/7 | 519  (total) | WMD or WMCD | -1.02 (-2.06, 0.01) | 63% | Low | Heyland MQS, Cochrane | High |
|  |  |  |  |  |  | SBP | 8/ 8 | 606  (total) | WMD or WMCD | -1.50 (-3.16, 0.16) | 59% | Very Low |  |  |
|  | Guar gum vs. controls | F,M; Healthy, some with OW,DM,HTN | EU,NA,  AS,SA | 4 w to 24 w | 18 to 70 | DBP | 5/5 | 279  (total) | WMD or WMCD | 0.39 (-2.25, 3.04) | 73% | Low |  |  |
|  |  |  |  |  |  | SBP | 5/5 | 279  (total) | WMD or WMCD | -0.19 (-3.11, 2.74) | 31% | Very Low |  |  |
|  | Konjac glucomannan vs. controls | F,M; Healthy, some with OW,DM,HTN | EU,NA | 4 w to 12 w | 20 to 69 | DBP | 2/2 | 92  (total) | WMD or WMCD | 3.63 (-4.28, 11.53) | 93% | Very Low |  |  |
|  |  |  |  |  |  | SBP | 2/ 2 | 92  (total) | WMD or WMCD | 1.00 (-8.13, 10.13) | 90% | Very Low |  |  |
|  | Pectin vs. controls | F,M; Healthy, some with OW,DM,HTN | EU,NA | 6 w to 12 w | 18 to 70 | DBP | 2/2 | 102  (total) | WMD or WMCD | -1.33 (-3.78, 1.12) | 0% | Very Low |  |  |
|  |  |  |  |  |  | SBP | 2/2 | 102  (total) | WMD or WMCD | -2.34 (-7.82, 3.14) | 34% | Very Low |  |  |
| Moraru, 2018 (Moraru et al., 2018) | Chitosan vs. controls | F,M; Healthy, some with Ow | EU,AS | 28 d to 365 d | 18 to 70 | DBP | 6/6 | 296/  293 | WMD | -2.14 (-4.14, -0.14)* | 99% | Very Low | NR | Low |
|  |  |  |  |  |  | SBP | 6/7 | 296/ 293 | WMD | -2.68 (-4.19, -1.18)* | 89% | Very Low |  |  |
| Whelton, 2005 (Whelton et al., 2005) | Dietary fibre vs. controls | F,M; Healthy, some with HTN | EU,NA,  AS,AU | 2 w to 26 w | 18 to 82 | DBP | 25/25 | 1477  (total) | WMCD | -1.65 (-2.70, -0.61)* | NR | Low | NR | Critically Low |
|  |  |  |  |  |  | SBP | 25/25 | 1477  (total) | WMCD | -1.15 (-2.68, 0.39) | NR | Low |  |  |
| ***Diet exposure: Sugars*** | | | | | | | | | | | | | | |
| Ha, 2012 (Ha et al., 2012) | Fructose vs. controls | F,M; Healthy, some with Ow,Ob,DM,O | EU,NA,  SA | 15.5 d to  10 w | 26 to 61 (m) | DBP | 11/11 | 352  (total) | MCD | -1.54 (-2.77, -0.32)* | 47% | Moderate | Heyland MQS | Moderate |
|  |  |  |  |  |  | SBP | 11/13 | 352  (total) | MCD | -1.10 (-2.64, 0.44) | 31% | Moderate |  |  |
| **Author, Year (Ref)** | **Comparison** | **Population** | **Regions** | **Study duration** | **Age range (mean)** | **Outcome** | **No. of primary studies/**  **estimates** | **No. of case/ control** |  | **BP Change (95%CI)*** | **I^2^** | **NutriGrade** | **Quality**  **tool** | **AMSTAR** |
| Te Morenga, 2014 (Te Morenga et al., 2014) | Free sugars vs. low sugars | F,M; Healthy, some with Ow, Ob,DM,  MetS | EU | 2 w to  6 m | From 18 | DBP | 12/12 | 324  (total) | WMCD | 1.37 (0.25, 2.49)* | 41% | Moderate | Cochrane | Moderate |
|  |  |  |  |  |  | SBP | 12/12 | 324  (total) | WMCD | 1.09 (-1.04, 3.22) | 67% | Moderate |  |  |
| ***Diet exposure: Proteins*** | | | | | | | | | | | | | | |
| Askarpour, 2019 (Askarpour et al., 2019) | L-carnitine supplements vs. controls | F,M; Healthy, some with DM, O | EU,AS,  AF, ME | 8 w to 54 w | 44 to 101 (m) | DBP | 10/10 | 427/  424 | WMCD | -1.16 (-2.02, -0.30)* | 0% | Moderate | Cochrane | Moderate |
|  |  |  |  |  |  | SBP | 10/10 | 427/ 424 | WMCD | -0.09 (-1.46, 1.29) | 41% | Low |  |  |
| Badely, 2019 (Badely et al., 2019) | Whey protein supplements vs. controls | F,M; Healthy | NR | 2 w to 96 w | From 18 | DBP | 18/18 | 885  (total) | WMCD | -5.65 (-6.69, -4.67)* | 99% | Low | Cochrane | Low |
|  |  |  |  |  |  | SBP | 18 26 | 885  (total) | WMCD | -7.46 (-9.39, -6.13)* | 100% | Low |  |  |
| Dong, 2011 (Dong et al., 2011) | Soy foods vs. controls | NR; Healthy | EU,NA | 4 w to 21 w | 45 to 62 (m) | DBP | 11/11 | 1608  (total) | WMCD | -1.41 (-3.52, 0.69) | 71% | Moderate | JADAD | Critically Low |
|  |  |  |  |  |  | SBP | 11/12 | 1608  (total) | WMCD | -2.78 (-6.53, 0.96) | 78% | Moderate |  |  |
|  | Soy protein isolate vs. controls | NR; Healthy | NR | 4 w to 52 w | 24 to 67 (m) | DBP | 14/14 | 1608  (total) | WMCD | -1.57 (-2.76, -0.38)* | 44% | Moderate |  |  |
|  |  |  |  |  |  | SBP | 15/15 | 1608  (total) | WMCD | -1.99 (-3.91, -0.07)* | 42% | Moderate |  |  |
|  | Soy protein vs. controls | F,M; Healthy, some with DM,HTN | EU,NA | 4 w to 52 w | 18 to 75 | DBP | 25/25 | 1608  (total) | WMCD | -1.44 (-2.56, -0.31)* | 62% | Moderate |  |  |
|  |  |  |  |  |  | SBP | 25/27 | 1608  (total) | WMCD | -2.21 (-4.10, -0.33)* | 66% | Moderate |  |  |
| Guan, 2020 (Guan, 2020) | Taurine supplements vs. controls | F,M; Healthy, some with Ob, O | AS,EU | 15 d to  6 m | From 18 | DBP | 5/5 | 109/  105 | WMCD | -2.90 (-4.29, -1.51)* | 0% | Moderate | Cochrane | Low |
|  |  |  |  |  |  | SBP | 6/6 | 118/  115 | WMCD | -4.68 (-9.10, -0.25)* | 79% | Moderate |  |  |
| Hidayat, 2017 (Hidayat et al., 2017) | Milk protein vs. controls | F,M; Healthy, some with Ow, Ob,  HTN,O | EU,NA,  AS,AU,  ME | 4 w to  2 y | 23 to 61 (m) | DBP | 7/7 | 412  (total) | WMCD | -1.80 (-3.38, -0.22)* | 0% | Low | JADAD | Moderate |
|  |  |  |  |  |  | SBP | 7 9 | 412  (total) | WMCD | -3.33 (-5.62, -1.03)* | 0% | Low |  |  |
| Mirenayat, 2018 (Mirenayat et al., 2018) | L-citrulline supplements vs. controls | F,M; Healthy, some with Ow,Ob | NA,AS | 1 w to  8 w | 21 to 70 (m) | DBP | 5/5 | 114  (total) | WMCD | -1.56 (-4.32, 1.20) | 68% | Low | JADAD | Moderate |
|  |  |  |  |  |  | SBP | 5/6 | 114  (total) | WMCD | 0.28 (-2.87, 2.31) | 7% | Low |  |  |
| **Author, Year (Ref)** | **Comparison** | **Population** | **Regions** | **Study duration** | **Age range (mean)** | **Outcome** | **No. of primary studies/**  **estimates** | **No. of case/ control** |  | **BP Change (95%CI)*** | **I^2^** | **NutriGrade** | **Quality**  **tool** | **AMSTAR** |
| Pripp, 2008 (Pripp, 2008) | Dietary peptides vs. controls | F,M; Healthy, some with HTN | NR | 4 w to 12 w | 46 to 77 (m) | DBP | 14/14 | 816  (total) | WMCD | -2.42 (-3.82, -1.03)* | NS | Low | NR | Critically Low |
|  |  |  |  |  |  | SBP | 15/17 | 826  (total) | WMCD | -5.13 (-7.12, -3.14)* | NR | Moderate |  |  |
| Rebholz, 2012 (Rebholz et al., 2012) | Dietary animal proteins vs. dietary vegetable proteins | F,M; Healthy, some with Ow, Ob,HTN | EU,NA,  AU | 4 w to 12 w | 18 to 79 | DBP | 12/12 | 1083  (total) | WMCD | -0.24 (-1.58, 1.10) | 78% | Low | NR | Critically Low |
|  |  |  |  |  |  | SBP | 12/12 | 1083  (total) | WMCD | -0.10 (-2.31, 2.11) | 97% | Low |  |  |
|  | Dietary protein vs. low dietary protein | F,M; Healthy, some with Ow, Ob,HTN | EU,NA,  AS, AU,  ME | 3 w to 52 w | 18 to 80 | DBP | 32/32 | 2594  (total) | WMCD | -1.15 (-1.59, -0.71)* | 0% | Low |  |  |
|  |  |  |  |  |  | SBP | 32/32 | 2594  (total) | WMCD | -1.76 (-2.33, -1.20)* | 0% | Low |  |  |
| Sohouli, 2021 (Sohouli, 2021) | Soy milk vs. controls | F,M; Healthy, some with DM | ME,EU | 4 w to 12 w | 22 to  51 (m) | DBP | 5/5 | 123/  123 | WMCD | -4.36 (-7.06, -1.66)* | 89% | Low | Cochrane | Low |
|  |  |  |  |  |  | SBP | 5/5 | 123/  123 | WMCD | -7.38 (-10.87, -3.88)* | 90% | Low |  |  |
| ***Diet exposure: Fats and oils*** | | | | | | | | | | | | | | |
| AbuMweis, 2018 (AbuMweis et al., 2018) | Eicosapentaenoic acid and/or docosahexaenoic acid supplements vs. controls | F,M; Healthy, some with DM, HTN,O | NR | 4 w to 240 w | 21 to 74 (m) | DBP | 50/50 | NR | MCD | -1.37 (-2.42, -0.33)* | 62% | Moderate | JADAD | Low |
|  |  |  |  |  |  | SBP | 50/50 | NR | MCD | -2.20 (-3.17, -1.22)* | 57% | Moderate |  |  |
| Guo, 2019 (Guo et al., 2019) | Docosahexaenoic acid supplements vs. controls | F,M; Healthy, some with DM, O | EU,NA,  AU | 4 w to  3 m | 33 to 61 (m) | DBP | 8/8 | 110/  109 | WMCD | -1.00 (-2.60, 0.60) | 0% | Low | JADAD | Moderate |
|  |  |  |  |  |  | SBP | 9/9 | 182/ 186 | WMCD | -1.30 (-3.20, 0.70) | 0% | Low |  |  |
|  | Eicosapentaenoic acid supplements vs. controls | F,M; Healthy, some with DM, O | EU,AS,  AU | 6 w to 13 m | 44 to 65 (m) | DBP | 7/7 | 75/  77 | WMCD | -1.10 (-2.80, 0.60) | 0% | Low |  |  |
|  |  |  |  |  |  | SBP | 8/8 | 75/  77 | WMCD | -2.60 (-4.60, -0.50)* | 0% | Low |  |  |
| Miller, 2014 (Miller et al., 2014) | Dietary and supplemental eicosapentaenoic acid and/or docosahexaenoic acid vs. controls | F,M; Healthy, some with HTN | EU,NA,  AS, AU,  ME | 21 d to 365 d | 18 to 90 | DBP | 69/92 | 4187  (total) | WMD | -0.99 (-1.54, -0.79)* | Sig | Very Low | NR | Critically Low |
|  |  |  |  |  |  | SBP | 70/93 | 4212  (total) | WMD | -1.52 (-2.25, -0.79)* | Sig | Very Low |  |  |
| Morris, 1993 (Morris et al., 1993) | Fish oil supplements vs. controls | F,M; Healthy, some with HTN,O | NR | 3 w to 24 w | 18 to 80 | DBP | 31/31 | 1356  (total) | WMCD | -1.50 (-2.20, -0.80)* | NS | Low | NR | Critically Low |
|  |  |  |  |  |  | SBP | 31/31 | 1356  (total) | WMCD | -3.00 (-4.50, -1.50)* | Sig | Low |  |  |
| **Author, Year (Ref)** | **Comparison** | **Population** | **Regions** | **Study duration** | **Age range (mean)** | **Outcome** | **No. of primary studies/**  **estimates** | **No. of case/ control** |  | **BP Change (95%CI)*** | **I^2^** | **NutriGrade** | **Quality**  **tool** | **AMSTAR** |
| Wendland, 2006 (Wendland et al., 2006) | Alpha-lipoic acid supplements vs. controls | F,M; Healthy, some with O | EU,AU | 6 w to 104 w | 46 to 55 (m) | DBP | 3/3 | 348  (total) | WMCD | -0.17 (-0.82, 0.48) | NR | Very Low | No | Critically Low |
|  |  |  |  |  |  | SBP | 3/3 | 348  (total) | WMCD | -0.72 (-2.01, 0.58) | NR | Very Low |  |  |
| Yang, 2015 (Yang et al., 2015) | Conjugated linoleic acid supplements vs. controls | F,M; Healthy, some with Ow,Ob,DM | EU,AS,  ME | 5 w to 24 w | 26 to 58 (m) | DBP | 8/8 | 318/  320 | WMCD | 0.69 (-1.41, 2.80) | 52% | Low | JADAD | Moderate |
|  |  |  |  |  |  | SBP | 8/9 | 318/ 320 | WMCD | -0.03 (-2.29, 2.24) | 22% | Low |  |  |
| Zamora-Zamora, 2018 (Zamora-Zamora et al., 2018) | Olive oil supplements vs. controls | F,M; Healthy, some with Ow, Ob,DM, HTN | EU,NA,  AS, AU | 12 w to 1 y | From 18 | SBP | 13/13 | 2853/ 2778 | MD | -0.11 (-0.68, 0.46) | 85% | Moderate | Cochrane | Low |
| ***Diet exposure: Minerals*** | | | | | | | | | | | | | | |
| Aburto, 2013 (Aburto et al., 2013) | Urinary potassium vs. controls | F,M; Healthy | EU,NA,  AS,AF,  AU,SA | 4 w to 12 m | From 18 | DBP | 22/22 | 893/  890 | MD | -1.96 (-3.06, -0.86)* | 55% | High | GRADE | Moderate |
|  |  |  |  |  |  | SBP | 22/22 | 947/ 945 | MD | -3.49 (-5.15, -1.82)* | 65% | High |  |  |
| Allender, 1996 (Allender et al., 1996) | Calcium supplements vs. controls | F,M; Healthy, some with HTN | NR | 5 d to  4 y | 18 to 70 | DBP | 22/22 | 1214  (total) | WMCD | -0.18 (-0.75, 0.40) | NR | Very Low | NR | Critically Low |
|  |  |  |  |  |  | SBP | 22/28 | 1223  (total) | WMCD | -0.89 (-1.74, -0.05)* | NR | Low |  |  |
| Beyer, 2006 (Beyer et al., 2006) | Potassium and magnesium supplements vs. controls | F,M; HTN | EU,NA,  AS | 24 w to 28 w | 21 to 70 (m) | DBP | 3/3 | 112/  118 | MCD | -3.84 (-9.47, 1.79) | 85% | Low | Cochrane | Moderate |
|  |  |  |  |  |  | SBP | 3/3 | 112/ 118 | MCD | -4.64 (-9.94, 0.66) | 68% | Low |  |  |
| Geleijnse, 2003 (Geleijnse et al., 2003) | Urinary sodium vs. salt reduction | F,M; Healthy, some with HTN | NR | >2 w | From 18 | DBP | 40/40 | NR | WMD | -1.96 (-2.56, -1.36)* | NR | Very Low | NR | Critically Low |
|  |  |  |  |  |  | SBP | 40/47 | NR | WMD | -2.54 (-3.47, -1.60)* | NR | Very Low |  |  |
| Ghanbari, 2021  (Ghanbari, 2021) | Chromium supplements vs. controls | F,M; Healthy, some with DM, Ob, MetS, O | EU,NA,  AS,ME | 12 w to  24 w | From 18 | DBP | 10/15 | 952  (total) | WMCD | -0.10 (-1.39, 1.18) | 38% | Moderate | Cochrane | Low |
|  |  |  |  |  |  | SBP | 10/15 | 952  (total) | WMCD | -0.42 (-2.15, 1.30) | 13% | Moderate |  |  |
| Griffith, 1999 (Griffith et al., 1999) | Dietary and supplemental calcium vs. controls | F,M; Healthy, some with HTN | NR | 2 w to 208 w | From 18 | DBP | 42/42 | 2068/ 2059 | MCD | -0.84 (-1.44, -0.24)* | Sig | Low | Quality Score of Studies | Moderate |
|  |  |  |  |  |  | SBP | 42/ 42 | 2068/ 2059 | MCD | -1.44 (-2.20, -0.68)* | Sig | Low |  |  |
| Hernandez, 2019 (Hernandez et al., 2019) | Sodium or salt substitute vs. regular salt | F,M; Healthy, some with HTN | EU,AS,  SA | 1 w to 44 m | 59 (m) | DBP | 19/19 | 958/ 974 | MD | -3.96 (-5.17, -2.74)* | 0% | Moderate | GRADE, Cochrane | Moderate |
|  |  |  |  |  |  | SBP | 19/21 | 960/ 973 | MD | -7.81 (-9.47, -6.15)* | 0% | Moderate |  |  |
| **Author, Year (Ref)** | **Comparison** | **Population** | **Regions** | **Study duration** | **Age range (mean)** | **Outcome** | **No. of primary studies/**  **estimates** | **No. of case/ control** |  | **BP Change (95%CI)*** | **I^2^** | **NutriGrade** | **Quality**  **tool** | **AMSTAR** |
| Khazdouz, 2020 (Khazdouz et al., 2020) | Zinc supplements vs. controls | F,M; Healthy, some with Ow,DM | AS,ME | 6 w to  3 m | 23 to 56 (m) | DBP | 4/4 | 101/  98 | SMD | 0.01 (-0.30, 0.32) | 0% | Very Low | Cochrane | Moderate |
|  |  |  |  |  |  | SBP | 4/ 4 | 101/  98 | SMD | -0.07 (-0.38, 0.24) | 0% | Very Low |  |  |
| Ndanuko, 2021 (Ndanuko, 2021) | Low vs. high urinary sodium to potassium ratio | F,M; Healthy,  some with HTN | NA,EU,  AU | NR | From 18 | DBP | 5/6 | 751/  600 | SMD | -1.42 (-2.24, -0.59)* | 98% | Low | NHMRC,  ADA | Low |
|  |  |  |  |  |  | SBP | 5/6 | 751/  600 | SMD | -1.09 (-1.91, -0.28)* | 97% | Low |  |  |
| Whelton, 1997 (Whelton et al., 1997) | Potassium supplements vs. controls | F,M; Healthy, some with HTN | NR | 4 d to  3 y | 18 to 79 | DBP | 32/32 | 2609  (total) | WMCD | -2.45 (-4.16, -0.74)* | Sig | Low | NR | Critically Low |
|  |  |  |  |  |  | SBP | 33/33 | 2609  (total) | WMCD | -4.44 (-6.36, -2.53)* | Sig | Low |  |  |
| Zhang, 2016 (Zhang et al., 2016) | Magnesium supplements vs. controls | F,M; Healthy | EU,NA,  AS, SA | 3 w to  6 m | 18 to 84 | DBP | 34/34 | 1010/ 1018 | WMCD | -1.77 (-2.82, -0.73)* | 64% | Moderate | JADAD, AHRQ | Moderate |
|  |  |  |  |  |  | SBP | 34/34 | 1010/ 1018 | WMCD | -2.00 (-3.58, -0.43)* | 62% | Moderate |  |  |
| ***Diet exposure: Vitamins*** | | | | | | | | | | | | | | |
| Asbaghi, 2021 (Asbaghi, 2021) | Folic acid supplements vs. controls | F,M; Healthy, some with HTN, O | EU,NA,  AS, SA, AU | 1 w to 234 w | From 18 | DBP | 20/24 | 20874/  20715 | WMCD | -0.24 (-0.37, -0.11)* | 48% | Moderate | Cochrane | Low |
|  |  |  |  |  |  | SBP | 22/26 | 20896/  20737 | WMCD | -1.11 (-1.93, -0.28)* | 65% | Moderate |  |  |
| Emami, 2019 (Emami et al., 2019) | Vitamin E supplements vs. controls | F,M; Healthy, some with DM, MetS, HTN,O | EU,NA,  AS, AU,  ME | 3 w to 48 w | 18 to 76 | DBP | 12/12 | 839  (total) | WMCD | -1.19 (-2.67, 0.29) | 90% | Low | JADAD | Low |
|  |  |  |  |  |  | SBP | 15/23 | 839  (total) | WMCD | -3.40 (-6.70, -0.11)* | 94% | Low |  |  |
| Golzarand, 2016 (Golzarand et al., 2016) | Vitamin D3 supplements vs. controls | F,M; Healthy, some with DM,HTN,O | EU,NA,  AS, AU,  ME | 1 m to 18 m | 55 (m) | DBP | 30/30 | 2338/ 2406 | WMCD | -0.57 (-1.36, 0.22) | 87% | Moderate | JADAD | Low |
|  |  |  |  |  |  | SBP | 30/41 | 2338/ 2406 | WMCD | -0.68 (-2.19, 0.84) | 92% | Moderate |  |  |
| Juraschek, 2012 (Juraschek et al., 2012) | Vitamin C supplements vs. controls | F,M; Healthy, some with Ow, DM,HTN,O | NR | 2 w to 26 w | 22 to 74 (m) | DBP | 29/29 | 1407  (total) | WMCD | -1.48 (-2.86, -0.10)* | 81% | Moderate | Own designed | Moderate |
|  |  |  |  |  |  | SBP | 29/29 | 1407  (total) | WMCD | -3.84 (-5.29, -2.38)* | 69% | Moderate |  |  |
| Li, 2015 (X. H. Li et al., 2015) | Active vitamin D vs. controls | NRNR; Healthy, some with O | NR | 1 m to 52 w | From 18 | DBP | 3/3 | 62/  64 | WMCD | -0.24 (-6.21, 5.72) | 64% | Low | Cochrane | Low |
|  |  |  |  |  |  | SBP | 3/3 | 62/  64 | WMCD | 0.30 (-4.95, 5.56) | 0% | Low |  |  |
| Li, 2018 (Li et al., 2018) | Multivitamin and multimineral vs. controls | F,M; Healthy, some with Ow, Ob,HTN | NR | 1 m to 86.4 m | 22 to 65 (m) | DBP | 10/10 | 2011  (total) | WMCD | -0.71 (-1.43, 0.00) | 31% | High | Cochrane | Moderate |
|  |  |  |  |  |  | SBP | 10/10 | 2011  (total) | WMCD | -1.31 (-2.48, -0.14)* | 28% | Moderate |  |  |
| **Author, Year (Ref)** | **Comparison** | **Population** | **Regions** | **Study duration** | **Age range (mean)** | **Outcome** | **No. of primary studies/**  **estimates** | **No. of case/ control** |  | **BP Change (95%CI)*** | **I^2^** | **NutriGrade** | **Quality**  **tool** | **AMSTAR** |
| Mirhosseini, 2018 (Mirhosseini et al., 2018) | Vitamin D supplements vs. controls | F,M; Healthy, some with Ow, Ob,DM,O | NR | 3 m to  5 y | 28 to 72 (m) | DBP | 39/39 | 2420/ 2420 | SMD | -0.07 (-0.14, -0.01)* | 18% | Moderate | Cochrane | Moderate |
|  |  |  |  |  |  | SBP | 39/ 39 | 2420/ 2420 | SMD | -0.10 (-0.19, -0.01)* | 51% | Moderate |  |  |
| Tabrizi, 2018 (Tabrizi et al., 2018) | Folate supplements vs. controls | F,M; Healthy, some with Ow, DM,  MetS,HTN | EU,AU,  ME | 4 w to 12 w | From 18 | DBP | 6/6 | 130/ 132 | SMD | -0.59 (-1.55, 0.37) | 92% | Low | Cochrane | Moderate |
|  |  |  |  |  |  | SBP | 6/6 | 130/ 132 | SMD | -0.87 (-1.83, 0.09) | 92% | Low |  |  |
| Wu, 2017 (Wu & Sun, 2017) | Vitamin D + Calcium vs. controls | F,M; Healthy, some with DM,O | NA,AS,  AU, ME | 8 w to  7 y | 20 to 62 (m) | DBP | 8/8 | 36806  (total) | WMCD | -0.22 (-0.89, 0.46) | 82% | Moderate | Cochrane | Moderate |
|  |  |  |  |  |  | SBP | 8/8 | 36806  (total) | WMCD | 0.61 (-0.95, 2.16) | 94% | Moderate |  |  |
| ***Diet exposure: Probiotics*** | | | | | | | | | | | | | | |
| Ghavami, 2020 (Ghavami, 2020) | Fermented milk supplements vs. controls | F,M; Healthy, some with HTN,O | EU,NA,  AS,ME,  AU | 2 w to 24 w | 34 to 77 (m) | DBP | 24/31 | 1058/  1044 | MCD | -1.04 (-2.51, 0.44) | 79% | Moderate | Cochrane | Low |
|  |  |  |  |  |  | SBP | 24/31 | 1058/  1044 | MCD | -2.17 (-4.50, 0.16) | 83% | Moderate |  |  |
| Lewis-Mikhael, 2020 (Lewis-Mikhael, 2020) | Lactobacillusplantarum supplements vs. controls | F,M; Healthy, some with HTN, DM, Ob, O | EU,NA,  AS,ME | 2 w to 12 w | 20 to 65 (m) | DBP | 7/8 | 337/  316 | WMCD | -0.92 (-1.49, -0.35)* | 0% | Moderate | JADAD | High |
|  |  |  |  |  |  | SBP | 7/8 | 337/  316 | WMCD | -1.58 (-3.05, -0.11)* | 36% | Moderate |  |  |
| Qi, 2020 (Qi, 2020) | Products with live bacteria vs. controls | F,M; Healthy, some with HTN,Ob,  DM,O | EU,NA,  AS,SA,  AU | 3 w to 24 w | 18 to 86 | DBP | 21/31 | 1509 (total) | WMD | -1.51 (-2.38, -0.65)* | 82% | High | Cochrane | Critically low |
|  |  |  |  |  |  | SBP | 21/31 | 1509 (total) | WMD | -3.05 (-4.67, -1.44)* | 91% | High |  |  |
| ***Diet exposure: Polyphenols*** | | | | | | | | | | | | | | |
| Akbari, 2019 (Akbari et al., 2019) | Resveratrol vs. controls | F,M; Healthy, some with MetS | EU,NA,  AS,AU,  ME,SA | 1 w to 48 w | 40 to 74 (m) | DBP | 27/27 | 948/ 800 | SMD | -0.21 (-0.52, 0.11) | 90% | Moderate | Cochrane | Low |
|  |  |  |  |  |  | SBP | 27/27 | 948/ 800 | SMD | -0.27 (-0.57, 0.03) | 89% | Moderate |  |  |
| Asbaghi, 2021 (Asbaghi, Naeini, et al., 2021) | Grape and its products vs. controls | F,M; Healthy, some with MetS,HTN,  DM,O | EU,NA,  AS, SA,  AU, ME | 2 w to  52 w | From 18 | DBP | 28/33 | 772/  729 | WMCD | -1.69 (-3.12, -0.27)* | 80% | High | Cochrane | High |
|  |  |  |  |  |  | SBP | 28/33 | 772/  729 | WMCD | -3.40 (-6.55, -0.24)* | 93% | High |  |  |
| **Author, Year (Ref)** | **Comparison** | **Population** | **Regions** | **Study duration** | **Age range (mean)** | **Outcome** | **No. of primary studies/**  **estimates** | **No. of case/ control** |  | **BP Change (95%CI)*** | **I^2^** | **NutriGrade** | **Quality**  **tool** | **AMSTAR** |
| Daneshzad, 2019 (Daneshzad et al., 2019) | Anthocyanin supplements vs. controls | F,M; Healthy | EU,AS,  AU,SA | 1 w to 96 w | From 18 | DBP | 13/13 | 513/ 472 | WMD | 0.66 (-0.82, 2.14) | 38% | Moderate | JADAD | Low |
|  |  |  |  |  |  | SBP | 13/15 | 513/ 472 | WMD | 1.80 (-0.92, 4.52) | 64% | Moderate |  |  |
| Ellwood, 2019 (Ellwood et al., 2019) | Flavonoid-rich fruits vs. controls | F,M; HTN | EU,NA,  AS,ME | ≤4 w | 21 to 77 (m) | DBP | 4/4 | 156/ 156 | WMD | -0.90 (-2.10, 0.31) | 0% | Low | JBI, GRADE | Moderate |
|  |  |  |  |  |  | SBP | 4/4 | 156/ 156 | WMD | -1.02 (-3.12, 1.07) | 0% | Low |  |  |
|  | Flavonoid-rich fruits vs. controls | F,M; HTN | EU,NA,  AS,ME | >4 w | 21 to 77 (m) | DBP | 5/5 | 196/ 150 | WMD | 0.86 (-1.11, 2.82) | 0% | Low |  |  |
|  |  |  |  |  |  | SBP | 5/5 | 196/ 150 | WMD | -0.95 (-3.58, 1.68) | 0% | Low |  |  |
| Feringa, 2011 (Feringa et al., 2011) | Grape seed extract vs. controls | F,M; Healthy, some with MetS,HTN,O | NR | 2 w to 24 w | From 18 | DBP | 5/5 | 228  (total) | WMCD | -0.65 (-1.67, 0.36) | 0% | Moderate | JADAD | Low |
|  |  |  |  |  |  | SBP | 5/7 | 228  (total) | WMCD | -1.54 (-2.85, -0.22)* | 11% | Moderate |  |  |
| Garcia-Conesa, 2018 (Garcia-Conesa et al., 2018) | Anthocyanins vs. standard (usual) diet | F,M; Healthy, some with Ow, MetS, HTN,O | EU,NA,  AS,AU,  ME,SA | 2 d to 730 d | 18 to 75 | DBP | 79/79 | NR | SMD | -0.20* | 25% | Low | Cochrane | Low |
|  |  |  |  |  |  | SBP | 74/74 | NR | SMD | -0.23* | 12% | Moderate |  |  |
|  | Hydrobenzoic acids vs. standard (usual) diet | F,M; Healthy, some with Ow, MetS, HTN,O | EU,NA,  AS,AU,  ME,SA | 2 d to 730 d | 18 to 75 | DBP | 20/20 | NR | SMD | -0.14 | 38% | Low |  |  |
|  |  |  |  |  |  | SBP | 21/21 | NR | SMD | -0.11 | 51% | Low |  |  |
| Hohmann, 2015 (Hohmann et al., 2015) | High phenolic olive oil vs. controls | F,M; Healthy, some with HTN,O | EU | 3 w to  2 m | 26 to 68 (m) | DBP | 2/2 | 69  (total) | SMD | -0.20 (-1.01, 0.62) | 94% | Low | Cochrane | Moderate |
|  |  |  |  |  |  | SBP | 2/2 | 69  (total) | SMD | -0.52 (-0.77, -0.27)* | 32% | Low |  |  |
| Li, 2015 (S. H. Li et al., 2015) | Grape polyphenols vs. controls | F,M; Healthy, some with MetS,HTN | NR | 2 w to 16 w | 31 to 64 (m) | DBP | 10/10 | 291/ 270 | WMCD | -0.50 (-1.46, 0.46) | 0% | Low | NR | Moderate |
|  |  |  |  |  |  | SBP | 10/12 | 291/ 270 | WMCD | -1.48 (-2.79, -0.16)* | 32% | Low |  |  |
| Menezes, 2017 (Menezes et al., 2017) | Quercetin vs. controls | F,M; Healthy, some with MetS,HTN,O | EU,NA,  AS,ME | 14 d to 84 d | 20 to 66 | DBP | 13/13 | 336/  334 | MD | -2.63 (-3.83, -1.42)* | 0% | Moderate | Cochrane, GRADE | Moderate |
|  |  |  |  |  |  | SBP | 13/15 | 336/ 334 | MD | -3.05 (-4.83, -1.27)* | 0% | Low |  |  |
| Mohammadi, 2019 (Mohammadi et al., 2019) | Hesperidin vs. controls | F,M; Healthy, some with Ow, Ob,DM,  MetS,HTN,O | EU,ME | 3 w to 12 w | 18 to 81 | DBP | 7/7 | 392  (total) | WMCD | -0.48 (-2.39, 1.42) | 23% | Low | Cochrane, NG | High |
|  |  |  |  |  |  | SBP | 7/8 | 392  (total) | WMCD | -0.85 (-3.07, 1.36) | 54% | Low |  |  |
| **Author, Year (Ref)** | **Comparison** | **Population** | **Regions** | **Study duration** | **Age range (mean)** | **Outcome** | **No. of primary studies/**  **estimates** | **No. of case/ control** |  | **BP Change (95%CI)*** | **I^2^** | **NutriGrade** | **Quality**  **tool** | **AMSTAR** |
| Raman, 2019 (Raman et al., 2019) | Flavanols vs. controls | NR; Healthy, some with O | EU,NA,  AS,AF,  AU,ME,  SA | 3 w to 26 w | From 18 | DBP | 91/91 | 4,208  (total) | MCD | -0.99 (-1.50, -0.45)* | 58% | Moderate | Cochrane and GRADE | Moderate |
|  |  |  |  |  |  | SBP | 91/91 | 4,208  (total) | MCD | -1.46 (-2.27, -0.65)* | 65% | Moderate |  |  |
| Ren, 2021  (Ren, 2021) | Proanthocyanidins vs. controls | F,M; Healthy | EU,AS | 5 w to 16 w | 44 to 57 (m) | DBP | 6/10 | 376  (total) | WMCD | -2.75 (-5.09, -0.41)* | 0% | Moderate |  | Critically low |
|  |  |  |  |  |  | SBP | 6/10 | 376  (total) | WMCD | -4.60 (-8.04, -1.16)* | 34% | Moderate |  |  |
| Shrime, 2011 (Shrime et al., 2011) | Flavonoid-rich cocoa vs. controls | F,M; Healthy, some with Ow, HTN,O | NR | 14 d to 126 d | 18 to 70 (m) | DBP | 20/20 | 914  (total) | WMCD | -1.2 (-2.5, 0.10) | NR | Low | NR | Low |
|  |  |  |  |  |  | SBP | 20/20 | 914  (total) | WMCD | -1.63 (-3.12, -0.13)* | 83% | Low |  |  |
| Weaver, 2021 (Weaver, 2021) | Red wine polyphenols vs. controls | F,M; Healthy, some with Ob, MetS, HTN,DM | NR | 3 w to 36 w | 21 to 65 | DBP | 25/33 | 973/  983 | WMCD | -0.97 (-2.25, 0.31) | 100% | Low | Cochrane | High |
|  |  |  |  |  |  | SBP | 25/33 | 973/  983 | WMCD | -2.62 (-4.81, -0.44)* | 99% | Low |  |  |
| ***Diet exposure: Phytochemicals*** | | | | | | | | | | | | | | |
| Amerizadeh, 2021 (Amerizadeh, 2021) | Genistein vs. controls | F,M; Healthy, some with MetS | EU,AS | 8 w to  1 y | 43 to  60 (m) | DBP | 5/6 | 242/  237 | SMD | -0.35(-0.80, 0.09) | 82% | Very low | Cochrane | Low |
|  |  |  |  |  |  | SBP | 5/6 | 242/  237 | SMD | -0.52(-0.90, -0.14)* | 74% | Very low |  |  |
| Ghaedi, 2020 (Ghaedi, 2020) | Phytosterols supplements vs. controls | F,M; Healthy, some with DM, Ob, MetS, O | EU,NA,  AS | 3 w to 52 w | 23 to 63 (m) | DBP | 19/22 | 791/  776 | WMCD | -0.84 (-1.60, -0.08)* | 0% | Moderate | Cochrane | Low |
|  |  |  |  |  |  | SBP | 19/22 | 791/  776 | WMCD | -1.55 (-2.67, -0.42)* | 16% | Moderate |  |  |
| Han, 2019 (Han et al., 2019) | Green coffee extract vs. controls | F,M; Healthy, some with OW,DM,HTN | AS,ME | 4 w to 16 w | From 18 | DBP | 5/5 | 251/  250 | WMCD | -2.17 (-2.75, -1.59)* | 47% | Moderate | Cochrane | Moderate |
|  |  |  |  |  |  | SBP | 5/9 | 251/ 250 | WMCD | -3.09 (-3.91, -2.27)* | 0% | Moderate |  |  |
| Ismail, 2021 (Ismail, 2021) | Olive leaf extract vs. controls | F,M; HTN | EU,ME | 8 w to 12 w | From 18 | DBP | 2/2 | 40/  40 | MD | -1.69 (-5.73, 2.34) | 0% | Low | GRADE | Low |
|  |  |  |  |  |  | SBP | 2/2 | 40/  40 | MD | -5.78 (-10.27, -1.30)* | 0% | Low |  |  |
| Li, 2020 (Li et al., 2020) | Green tea extract vs. controls | F,M; Healthy, some with  MetS | EU,NA,  AS,SA | 6 w to 24 w | 40 to 52 (m) | DBP | 8/8 | 270/  267 | SMD or WMDC or WMD | -0.21 (-0.46, -0.04)* | 63% | Moderate | JADAD | Moderate |
|  |  |  |  |  |  | SBP | 8/8 | 270/ 267 | SMD or WMDC or WMD | -0.19 (-0.52, 0.14) | 79% | Moderate |  |  |
|  | Tea extract vs. controls | F,M; Healthy, some with  MetS | EU,NA,  AS,SA | 6 w to 24 w | 40 to 52 (m) | DBP | 9/9 | 386/ 377 | SMD or WMDC or WMD | -0.16 (-0.41, 0.09) | 64% | Moderate |  |  |
|  |  |  |  |  |  | SBP | 9/12 | 386/ 377 | SMD or WMDC or WMD | -0.16 (-0.47, 0.16) | 77% | Moderate |  |  |
| **Author, Year (Ref)** | **Comparison** | **Population** | **Regions** | **Study duration** | **Age range (mean)** | **Outcome** | **No. of primary studies/**  **estimates** | **No. of case/ control** |  | **BP Change (95%CI)*** | **I^2^** | **NutriGrade** | **Quality**  **tool** | **AMSTAR** |
| Noordzij, 2005 (Noordzij et al., 2005) | Caffeine vs. controls | F,M; Healthy | NR | 7 d to  84 d | 23 to 71 (m) | DBP | 5/5 | 159  (total) | WMCD | 1.72 (0.44, 3.00)* | NR | Very Low | NR | Critically Low |
|  |  |  |  |  |  | SBP | 5/7 | 159  (total) | WMCD | 3.64 (1.57, 5.71)* | NR | Very Low |  |  |
| Onakpoya, 2015 (Onakpoya, Spencer, et al., 2015) | Chlorogenic acid vs. controls | NR; Healthy, some with HTN | AS | 4 w to 26 w | 22 to 65 (m) | DBP | 5/5 | 253/  254 | MCD | -2.54 (-3.93, -1.15)* | 97% | Moderate | NR | Moderate |
|  |  |  |  |  |  | SBP | 5/10 | 253/ 254 | MCD | -4.31 (-5.60, -3.01)* | 65% | Moderate |  |  |
| Tierney, 2020  (Tierney, 2020) | Lycopene supplements vs. controls | F,M; Healthy, some with HTN, MetS,O | EU,ME,AS | 2 w to  16 w | From 18 | DBP | 7/8 | 288/  254 | MCD | -0.46 (-2.43. 1.51) | 30% | Moderate | Cochrane | High |
|  |  |  |  |  |  | SBP | 8/9 | 325/  292 | MCD | -0.16 (-4.09, 3.76) | 75% | Moderate |  |  |
| ***Diet exposure: Nitrates*** | | | | | | | | | | | | | | |
| He, 2021 (He, 2021) | Nitrates vs. controls | F,M; Healthy, some with  Ow, DM, O | NR | 45 min to 4 w | Over 60 | DBP | 22/30 | 372 (total) | WMD | -2.62 (-3.86, -1.37)* | 31% | Moderate | Cochrane | Low |
|  |  |  |  |  |  | SBP | 22/30 | 372 (total) | WMD | -3.90 (-5.23, -2.57)* | 17% | High |  |  |
| ***Diet exposure: Sweeteners*** | | | | | | | | | | | | | | |
| Onakpoya, 2015 (Onakpoya & Heneghan, 2015) | Steviol glycoside vs. controls | F,M; Healthy, some with Ow,Ob,DM,  HTN | EU,NA,  AS,ME | 2 h to 104 w | 18 to 75 | DBP | 7/7 | 395/  393 | MCD | -2.24 (-4.20, -0.27)* | 100% | High | Cochrane | Moderate |
|  |  |  |  |  |  | SBP | 7/11 | 395/ 393 | MCD | -2.98 (-6.23, 0.27) | 100% | Moderate |  |  |
|  | Stevioside vs. controls | F,M; Healthy, some with Ow,Ob,DM,  HTN | EU,NA,  AS,ME | 2 h to 104 w | From 18 | DBP | NR | 380  (total) | MCD | -2.69 (-5.12, -0.26)* | 100% | Moderate |  |  |
|  |  |  |  |  |  | SBP | NR | 380  (total) | MCD | -4.45 (-8.48, -0.41)* | 100% | Moderate |  |  |
|  | Pure rebaudioside vs. controls | F,M; Healthy, some with Ow,Ob,DM,  HTN | NR | 6 h to  16 w | From 18 | DBP | NR | 408  (total) | MCD | -1.38 (-3.85, 1.10) | 86% | Moderate |  |  |
|  |  |  |  |  |  | SBP | NR | 408  (total) | MCD | -0.36 (-1.70, 0.99) | 0% | Moderate |  |  |
| ***Diet exposure: Others*** | | | | | | | | | | | | | | |
| Xia, 2020  (Xia, 2020) | Astaxanthin supplements vs. controls | F.M; Healthy, some with  DM, O | AU, AS,  ME | 8 w to 12 m | 43 to 56 (m) | DBP | 5/8 | 134/  127 | WMCD | -1.77 (-3.62, 0.07) | 0% | Moderate | Cochrane | Critically Low |
|  |  |  |  |  |  | SBP | 5/8 | 134/  127 | WMCD | -3.33 (-7.84, 1.17) | 0% | Moderate |  |  |

*P<0.05; (m): mean age

1 ADA: American Dietetic Association Evidence Analysis Manual, AF: Africa, AHRQ: Agency for Healthcare Research and Quality evaluation criteria, AND: Academy of Nutrition and Dietetics quality criteria, AS: Asia, AU: Australia and New Zealand, d: day, DBP: Diastolic blood pressure, DM: Diabetes mellitus, EU: Europe, F: Female, h: hour, HTN: Hypertension, JBI: JBI Database of Systematic Reviews and Implementation Reports, M: Males, m: month, MD: Mean difference, MCD: Mean change difference, ME: Middle East, MetS: Metabolic symptom, MQS: Heyland methodological quality score, NA: North America, NG: NutriGrade, NHLBI: Quality Assessment of Controlled Intervention Studies Tool by the National Heart, Lung, and Blood Institute, NHMRC: Australian National Health and Medical Research Council criteria, NR: Not reported, O: Other medical conditions, Ob: Obese, Ow: Overweight, SA: South America, SBP: Systolic blood pressure, SIGN :Scottish Intercollegiate Guidelines Network, SMD: Standard mean difference, w: week, WMD: Weighted mean difference, WMCD: Weighted mean change difference, y: year.

**Supplementary Table 5 Summary of observational studies by diet exposure**

| **Author, Year (Refence)** | **Comparison** | **Population** | **Regions** | **Study duration** | **Age range (mean)** | **Outcome** | **No. of primary studies/**  **estimates** | **No. of**  **cases/non-cases** |  | **BP Change (95%CI)*** | **I^2^** | **NutriGrade** | **Quality**  **tool** | **AMSTAR** |
| --- | --- | --- | --- | --- | --- | --- | --- | --- | --- | --- | --- | --- | --- | --- |
| ***Diet exposure: Patterns of diet*** | | | | | | | | | | | | | | |
| Benatar, 2018 (Benatar & Stewart, 2018)  (cross-sectional) | Vegan vs. omnivore | F,M; Healthy | EU,NA,  AS,AF,  AU | NR | 28 to 72 | DBP | 19/19 | 3,222/  53,870 | MD | -1.33 (-2.67, 0.02)* | 82% | Very Low | NOS | Moderate |
|  |  |  |  |  |  | SBP | 19/19 | 3,222/  53,870 | MD | -2.56 (-4.66, -0.45)* | 83% | Low |  |  |
| Farhangi, 2020 (Farhangi et al., 2020)  (cross-sectional, case-control, cohort) | Highest vs. lowest dietary inflammatory index | F,M; Healthy, some with Ow,Ob,DM | EU,NA,  ME,SA | NR | 18 to 90 | DBP | 10/12 | 19,653/  19,579 | WMD | 0.01 (-0.69, 0.70) | 92% | Low | NOS | Moderate |
|  |  |  |  |  |  | SBP | 12/15 | 21,486/  21,411 | WMD | 1.23 (0.28, 2.18)* | 92% | Low |  |  |
|  |  |  | EU,NA,  AS,AU,  ME |  |  | HTN | 12/15 | 20,126/  23,976 | OR | 1.13 (1.01, 1.27)* | 56% | Low |  |  |
| Godos, 2017 (Godos et al., 2017)  (cross-sectional, prospective cohort) | Highest vs. lowest adherence to Mediterranean diet | F,M; Healthy, some with DM | EU,ME | 3 y | 18 to 74 | HTN | 4/6 | 33,847  (total) | RR | 0.87 (0.77, 0.97)* | 0% | Very Low | NOS | Low |
| Parohan, 2019 (Parohan et al., 2019)  (cross-sectional, prospective cohort) | An increase of 40 units in net endogenous acid production (NEAP) values | F,M; Healthy | EU,NA,  AS | 4 y to  14 y | 18 to 79 | HTN | 11/12 | 55,443/  287,144 | RR | 1.01 (0.97, 1.06) | 78% | Very Low | NOS | Moderate |
|  | An increase of 20 units increase in potential renal acid load (PRAL) values | F,M; Healthy | EU,NA,  AS | 4 y to  14 y | 18 to 79 | HTN | 9/10 | 39,660/  197,668 | RR | 1.03 (1.01, 1.06)* | 44% | Very Low |  |  |
| Dehghan, 2020 (Dehghan & Abbasalizad Farhangi, 2020) | Highest vs. lowest NEAP | F,M; Healthy | EU,NA,  AS, ME | NR | 18 to 84 | SBP | 6 | 42884/42882 | WMD | 0.50 (-0.29, 1.28) | 47% | Very Low | NOS | High |
|  |  |  |  |  |  | DBP | 3 |  | WMD | 0.03 (-1.07, 1.13) |  |  |  |  |
|  | Highest vs. lowest PRAL | F,M; Healthy | EU,NA,  AS, ME | NR | 18 to 84 | SBP | 12 | 55790/55788 | WMD | 0.98 (0.51, 1.45) | 0% | Very Low |  |  |
|  |  |  |  |  |  | DBP | 8 | 22,113 | WMD | 0.61 (0.09, 1.14) |  |  |  |  |
| **Author, Year (Refence)** | **Comparison** | **Population** | **Regions** | **Study duration** | **Age range (mean)** | **Outcome** | **No. of primary studies/**  **estimates** | **No. of**  **cases/**  **participants** |  | **BP Change (95%CI)*** | **I^2^** | **NutriGrade** | **Quality**  **tool** | **AMSTAR** |
| Picasso, 2019 (Picasso et al., 2019)  (cross-sectional, cohort) | Vegetarian vs. omnivore | F,M; Healthy | EU,NA,  AS,AF,  AU,SA | NR | 29 to 68 (m) | DBP | 41/42 | 12,732/ | WMD | -3.03 (-4.93, -1.13)* | 98% | Low | NOS,  NIH | Moderate |
|  |  |  |  |  |  | SBP | 41/42 | 20,915/  63,028 | WMD | -4.18 (-5.57, -2.80)* | 90% | Low |  |  |
| Wang, 2016 (Wang et al., 2016)  (cross-sectional, cohort) | Highest vs. lowest healthy dietary pattern | F,M; Healthy | EU,NA,  AS,AF,  ME,SA | NR | 18 to 84 | HTN | 22/24 | 14,796/  81,683 | OR | 0.81 (0.67, 0.97)* | 94% | Very Low | NOS,  NIH | Moderate |
| ***Diet exposure: Meat, poultry, egg, and fish*** | | | | | | | | | | | | | | |
| Schwingshackl, 2017 (Schwingshackl et al., 2017)  (prospective cohort, case-cohort, and nested case-control design) | Highest vs. lowest fish intake | F,M; Healthy | EU,NA,  ME | 3 y to  20 y; 564k to 1.4m person years | 25 to 84 | HTN | 6/8 | 83,612/  556,913 | RR | 1.01 (0.92, 1.10) | 57% | Very Low | NG | Moderate |
|  | An increase fish intake of 100 g/d |  |  |  |  | HTN | 5/7 |  | RR | 1.07 (0.98, 1.16) | 74% | N/A |  |  |
|  | Highest vs. lowest red meat intake | F,M; Healthy | EU,ME | 3 y to  14 y; 564k to 1.4m person years | 25 to 84 | HTN | 5/7 | 97,745/  264,148 | RR | 1.15 (1.02, 1.28)* | 84% | Very Low |  |  |
|  | An increase of red meat intake of 100 g/d |  |  |  |  | HTN | 5/7 |  | RR | 1.14 (1.02, 1.28)* | 88% | N/A |  |  |
|  | Highest vs. lowest processed meat intake | F,M; Healthy | EU,NA | 10 y to 14 y; 564k to 1.4m person years | 25 to 75 | HTN | 3/5 | 97,441/  262,447 | RR | 1.12 (1.02, 1.23)* | 81% | Very Low |  |  |
|  | An increase of processed meat intake of  50 g/d |  |  |  |  | HTN | 2/5 |  | RR | 1.12 (1.00, 1.26) | 82% | N/A |  |  |
| **Author, Year (Refence)** | **Comparison** | **Population** | **Regions** | **Study duration** | **Age range (mean)** | **Outcome** | **No. of primary studies/**  **estimates** | **No. of**  **cases/**  **participants** |  | **BP Change (95%CI)*** | **I^2^** | **NutriGrade** | **Quality**  **tool** | **AMSTAR** |
| Zhang, 2018 (Zhang & Zhang, 2018) | Highest vs. lowest egg intake | F,M; Healthy, some with DM,O | NA,AS | 3 y to  15 y | 18 to 84 | HTN | 3/3 | 1,987/  8,942 | RR | 0.79 (0.68, 0.91)* | 13% | Low | NOS | Moderate |
|  | Highest vs. lowest poultry intake | F,M; Healthy, some with DM,O | NA,ME | 3 y to  26 y | 18 to 84 | HTN | 4/6 | 14,739/  222,740 | RR | 1.15 (1.03, 1.28)* | 63% | Very Low |  |  |
|  | Highest vs. lowest total meat intake | F,M; Healthy, some with DM,O | EU,NA,  ME | 3 y to  26 y | 18 to 84 | HTN | 7/9 | 28,288/  351,817 | RR | 1.22 (1.11, 1.35)* | 75% | Low |  |  |
| ***Diet exposure: Milk and dairy*** | | | | | | | | | | | | | | |
| Heidari, 2021 (Heidari, 2021)  (prospective cohort) | Highest vs. lowest dairy products intake | F,M; Healthy | EU,NA,  ME,AS | 2.2 y to  30 y | From 18 | HTN | 15/19 | 119,579/  351,932 | RR | 0.90 (0.87, 0.94)* | 49% | Moderate | NOS, AMSTAR2, GRADE | Low |
|  | Highest vs. lowest low fat dairy products intake | F,M; Healthy | EU,NA,  ME | 2.2 y to 14.6 y | From 18 | HTN | 8/10 | 12,800/  48,799 | RR | 0.86 (0.77, 0.96)* | 72% | Moderate |  |  |
|  | Highest vs. lowest whole fat dairy products intake | F,M; Healthy | EU,NA,  ME | 2.2 y to 14.6 y | From 18 | HTN | 8/10 | 12,800/  48,799 | RR | 0.99 (0.94, 1.06) | 81% | Low |  |  |
|  | Highest vs. lowest milk intake | F,M; Healthy | EU,NA,  ME, AS | 3 y to 14.6 y | From 18 | HTN | 11/13 | 34,551/  152,867 (with 2 cohorts NR cases) | RR | 0.94 (0.90, 0.99)* | 74% | Moderate |  |  |
|  | Highest vs. lowest fermented dairy intake | F,M; Healthy | EU,NA,  ME | 3 y to 14.6 y | From 18 | HTN | 8/11 | 94,337/  262,388 (with 2 cohorts NR cases) | RR | 0.95 (0.91, 0.99)* | 55% | Moderate |  |  |
|  | Highest vs. lowest yogurt intake | F,M; Healthy | EU,NA,  ME | 3 y to 14.6 y | From 18 | HTN | 6/11 | 102,124/  261,545 (1 cohort NR cases) | RR | 0.95 (0.90, 1.01) | 60% | Low |  |  |
| **Author, Year (Refence)** | **Comparison** | **Population** | **Regions** | **Study duration** | **Age range (mean)** | **Outcome** | **No. of primary studies/**  **estimates** | **No. of**  **cases/**  **participants** |  | **BP Change (95%CI)*** | **I^2^** | **NutriGrade** | **Quality**  **tool** | **AMSTAR** |
|  | Highest vs. lowest cheese intake | F,M; Healthy | EU,NA,  ME | 3 y to 14.6 y | From 18 | HTN | 9/10 | 102,838/  292,681 (2 cohorts NR cases) | RR | 0.97 (0.92, 1.01) | 53% | Low |  |  |
| Lee, 2018 (Lee et al., 2018)  (cross-sectional, nested case-control, cohort) | An increase of milk intake of 200 g/d | F,M; Healthy, some with MetS | EU,NA,  AS | NR | 18 to 84 | HTN | 5/5 | 22,393  (total) | RR | 0.97 (0.91, 1.03) | 0% | N/A | NOS,  STROBE | Moderate |
| Ralston, 2012 (Ralston et al., 2012)  (Prospective cohort) | Highest vs. lowest fluid dairy products intake | F,M; Healthy | EU,NA | 5 y to  15 y | From 18 | HTN | 4/6 | 20,323/  72,079 | RR | 0.92 (0.87, 0.98)* | 0% | Very Low | ADA | Moderate |
| Schwingshackl, 2017 (Schwingshackl et al., 2017)  (prospective cohort, case-cohort, and nested case-control design) | An increase of dairy intake of 200 d/day | F,M; Healthy | EU,NA,  AS | 2 y to  15 y | From 18 | HTN | 9/9 | 31,509/  116,415 | RR | 0.95 (0.94, 0.97)* | 0% | N/A | NG | Moderate |
| Soedamah-Muthu, 2012 (Soedamah-Muthu et al., 2012)  (prospective cohort) | An increase of low fat dairy intake of 200 g/d | F,M; Healthy | EU,NA | of 2 y to 15 y | From 18 | HTN | 6/6 | 11,365/  42,407 | RR | 0.96 (0.93, 0.99)* | 25% | Very Low | NR | Low |
| **Author, Year (Refence)** | **Comparison** | **Population** | **Regions** | **Study duration** | **Age range (mean)** | **Outcome** | **No. of primary studies/**  **estimates** | **No. of**  **cases/**  **participants** |  | **BP Change (95%CI)*** | **I^2^** | **NutriGrade** | **Quality**  **tool** | **AMSTAR** |
| ***Diet exposure: Fruits and vegetables*** | | | | | | | | | | | | | | |
| Wu, 2016 (Wu et al., 2016)  (prospective cohort) | Highest vs. lowest fruit intake | F,M; Healthy | EU,NA,  AS | 3.8 y to 28 y | 25 to 65 | HTN | 6/8 | 94,797/  185,676 | RR | 0.87 (0.79, 0.95)* | 64% | Low | NOS | Moderate |
|  | An increase of fruit intake of serving/d |  |  |  |  | HTN | 5/7 | 92,491/  164,784 | RR | 0.98 (0.97, 0.99)* | NR | N/A |  |  |
|  | highest vs. lowest vegetable intake | F,M; Healthy | EU,NA,  AS | 3.8 y to 28 y | 18 to 95 | HTN | 8/8 | 94,797/  185,676 | RR | 0.88 (0.79, 0.99)* | 68% | Low |  |  |
|  | An increase of vegetable intake of serving/d |  |  |  |  | HTN | 7/7 | 92,491/  164,784 | RR | 1.00 (0.99, 1.01) | NR | N/A |  |  |
|  | Highest vs lowest fruit and vegetable intake | F,M; Healthy | EU,NA | 3.8 y to 28 y | 18 to 95 | HTN | 6/6 | 91,592/  160,284 | RR | 0.90 (0.84, 0.98)* | 68% | Low |  |  |
|  | An increase of fruit and vegetable intake of serving/d |  |  |  |  | HTN | 6/6 | 91,592/  160,284 | RR | 0.99 (0.99, 0.99) | NR | N/A |  |  |
| ***Diet exposure: Starchy foods*** | | | | | | | | | | | | | | |
| Schwingshackl, 2017 (Schwingshackl et al., 2017)  (prospective cohort, case-cohort, and nested case-control design) | Highest vs. lowest whole grains intake | F,M; Healthy | NA | 9 y to  18 y | 18 to 95 | HTN | 4/4 | 28,069  /109,615 | RR | 0.86 (0.79, 0.93)* | 72% | Very Low | NG | Moderate |
|  | An increase of whole grains intake of 30 g/day |  |  |  |  | HTN | 4/4 |  | RR | 0.92 (0.87, 0.98)* | 88% | N/A |  |  |
|  | Highest vs. lowest refined grains intake | F,M; Healthy | NA | 9 y to  16 y | 18 to 95 | HTN | 3/3 | 18,842/  58,086 | RR | 0.95 (0.88, 1.03) | 17% | Very Low |  |  |
|  | An increase of refined grains intake 30 g/day |  |  |  |  | HTN | 3/3 |  | RR | 0.99 (0.96, 1.02) | 37% | N/A |  |  |
| **Author, Year (Refence)** | **Comparison** | **Population** | **Regions** | **Study duration** | **Age range (mean)** | **Outcome** | **No. of primary studies/**  **estimates** | **No. of**  **cases/**  **participants** |  | **BP Change (95%CI)*** | **I^2^** | **NutriGrade** | **Quality**  **tool** | **AMSTAR** |
| Schwingshackl, 2019 (Schwingshackl et al., 2019)  (cohort, case–cohort, and nested case–control design, as well as fol-low-ups of RCTs) | An increase of baked/boiled/mashed potatoes of 150 g/d | F,M; Healthy | EU,NA | 6.7 yr; 560k to 1.3m person years | 40 to 86 | HTN | 2/4 | 78,484/  201,290 | RR | 1.08 (0.96, 1.21) | 84% | Very Low | NG,  MOOSE | Low |
|  | An increase of French fries of 150 g/d | F,M; Healthy | EU,NA | 6.7 yr; 560k to 1.3m person years | 40 to 86 | HTN | 2/4 | 78,484/  201,290 | RR | 1.37 (1.15, 1.63)* | 69% | Very Low |  |  |
|  | Highest vs. lowest potatoes intake | F,M; Healthy | EU,NA | 6.7 yr; 560k to 1.3m person years | 40 to 86 | HTN | 2/4 | 78,484/  201,290 | RR | 1.09 (0.92, 1.29) | 71% | Very Low |  |  |
|  | An increase of potatoes intake of 150 g/d |  |  |  |  | HTN | 2/4 |  | RR | 1.12 (1.01, 1.23) | 87% | N/A |  |  |
| ***Diet exposure: Legumes and pulses*** | | | | | | | | | | | | | | |
| Schwingshackl, 2017 (Schwingshackl et al., 2017)  (prospective cohort, case-cohort, and nested case-control design) | Highest vs. lowest legumes intake | F,M; Healthy | EU,NA,  ME | 2 y to  15 y | 25 to 75 | HTN | 4/6 | 80,871  /213,805 | RR | 0.92 (0.86, 0.98)* | 0% | Very Low | NG | Moderate |
|  | An increase of legumes intake of 50 g/day (n=5) |  |  |  |  | HTN | 3/5 |  | RR | 0.98 (0.95, 1.01) | 0% | N/A |  |  |
| ***Diet exposure: Nuts and seeds*** | | | | | | | | | | | | | | |
| Schwingshackl, 2017 (Schwingshackl et al., 2017)  (prospective cohort, case-cohort, and nested case-control design) | Highest vs. lowest nuts intake | F,M; Healthy | EU,NA,  ME | 3 y to  9 y | 25 to 75 | HTN | 4/4 | 11,962/  42,829 | RR | 0.85 (0.78, 0.92)* | 0% | Very Low | NG | Moderate |
|  | An increase by 28 g/day |  |  |  |  | HTN | 4/4 |  | RR | 0.70 (0.45, 1.08) | 69% | N/A |  |  |
| **Author, Year (Refence)** | **Comparison** | **Population** | **Regions** | **Study duration** | **Age range (mean)** | **Outcome** | **No. of primary studies/**  **estimates** | **No. of**  **cases/**  **participants** |  | **BP Change (95%CI)*** | **I^2^** | **NutriGrade** | **Quality**  **tool** | **AMSTAR** |
| ***Diet exposure: Cocoa*** | | | | | | | | | | | | | | |
| Morze, 2020 (Morze et al., 2020)  (cohorts, case–cohorts, case–control nested in cohort studies, as well as follow-ups of randomized controlled trials) | Highest vs. lowest chocolate intake | F,M; Healthy | EU | 2 y to 13.8 y | From 18 | HTN | 2/2 | 9,530/  46,454 | RR | 0.97 (0.91, 1.04) | 0% | Very Low | NG,  MOOSE | Moderate |
| ***Diet exposure: Beverages*** | | | | | | | | | | | | | | |
| Roerecke, 2018 (Roerecke et al., 2018)  (cohort studies, including nested case-control studies) | 5 alcoholic drinks/d vs. non-drinker | M; Healthy | EU,NA,  AS | 3.9 y to 20 y | From 18 | HTN | 9/10 | 32,426/  125,907 | RR | 1.68 (1.31, 2.14)* | 94% | Low | GRADE, Cochrane | Moderate |
|  | 3 alcoholic drinks/d vs. non-drinker | F; Healthy | EU,NA,  AS | 3.9 y to 20 y |  | HTN | 7/7 | 57,734/  235,347 | RR | 1.46 (1.22, 1.76)* | 89% | Low |  |  |
|  |  |  |  |  |  |  |  |  |  |  |  |  |  |  |
| Xie, 2018 (Xie et al., 2018)  (cohort studies) | Highest vs. lowest coffee intake | F,M; Healthy | EU,NA,  AS | 3 y to  33 y | From 18 | HTN | 10/10 | 58,094/  243,869 | RR | 0.95 (0.91, 0.99)* | 44% | Low | NOS | Moderate |
|  | An increase coffee intake of 1 cup/d |  |  |  |  | HTN | 8/10 |  | RR | 0.98 (0.98, 0.99)* | 43% | N/A |  |  |
| **Author, Year (Refence)** | **Comparison** | **Population** | **Regions** | **Study duration** | **Age range (mean)** | **Outcome** | **No. of primary studies/**  **estimates** | **No. of**  **cases/**  **participants** |  | **BP Change (95%CI)*** | **I^2^** | **NutriGrade** | **Quality**  **tool** | **AMSTAR** |
| Azad, 2017 (Azad et al., 2017) | Highest vs. lowest artificial-sweetened beverages intake | F,M; Healthy | NA | 5 y to  38 y | From 18 | HTN | 3/5 | 232,630  (total) | HR | 1.12 (1.08, 1.13)** | 53% | Very Low | NOS,  Cochrane | Moderate |
| Kim, 2016 (Kim & Je, 2016) | An increase of artificial-sweetened beverages by 1 serving/d | F,M; Healthy | NA | 7 y to  38 y | 36 to 58 (m) | HTN | 4/4 | 78,177/  227,254 | RR | 1.09 (1.06, 1.11)* | 60% | Very Low | NOS | Moderate |
| Kim, 2016 (Kim & Je, 2016)  (prospective cohort) | Highest vs. lowest sugar-sweetened beverages intake | F,M; Healthy | EU,NA | 4 y to  38 y | 36 to 58 (m) | HTN | 6/6 | 80,628/  246,828 | RR | 1.12 (1.07, 1.17)* | 60% | Low | NOS | Moderate |
|  | An increase of sugar-sweetened beverages of 1 serving/d |  |  |  |  | HTN | 6/6 |  | RR | 1.08 (1.06, 1.11)* | NR | N/A |  |  |
| ***Diet exposure: Sugars*** | | | | | | | | | | | | | | |
| Jayalath, 2014 (Jayalath, Sievenpiper, et al., 2014)  (prospective cohort) | Highest vs. lowest total fructose intake | F,M; Healthy, some with HTN | NA | 14 y to 20 y | 25 to 57 | HTN | 3/3 | 58,162  /223,230 | RR | 1.02 (0.99, 1.04) | 0% | Very Low | NOS | Moderate |
| ***Diet exposure: Proteins*** | | | | | | | | | | | | | | |
| Liu, 2002 (Liu et al., 2002)  (cross-sectional studies) | Pooled regression coefficient of dietary protein intake | F,M; Healthy | NA,AS | NR | 25 to 60 | DBP | 7/9 | 12,508  (total) | Regression coefficient | -0.014 (0.00)* | NR | Very Low | NR | Critically Low |
|  |  |  |  |  | 26 to 63 (m) | SBP | 7/9 | 12,761  (total) | Regression coefficient | -0.029 (0.01)* | NR | Very Low |  |  |
|  | | | | | | | | | | | | | | |
| **Author, Year (Refence)** | **Comparison** | **Population** | **Regions** | **Study duration** | **Age range (mean)** | **Outcome** | **No. of primary studies/**  **estimates** | **No. of**  **cases/**  **participants** |  | **BP Change (95%CI)*** | **I^2^** | **NutriGrade** | **Quality**  **tool** | **AMSTAR** |
| ***Diet exposure: Fats and oils*** | | | | | | | | | | | | | | |
| Yang, 2016 (Yang et al., 2016)  (prospective cohort) | Highest vs. lowest dietary long-chain n-3 polyunsaturated fatty acid | F,M; Healthy | NA,AS | 3 y to  20 y | 25 to 62 | HTN | 4/4 | 15,234/  36,112 | RR | 0.80 (0.58, 1.10) | 79% | Very Low | NR | Low |
|  | Highest vs. lowest biomarker of long-chain n-3 polyunsaturated fatty acid | F,M; Healthy | EU,NA,  AS | 3 y to  20 y | 25 to 62 (m) | HTN | 3/3 | 1,662/  3,898 | RR | 0.67 (0.55, 0.83)* | 47% | Very Low |  |  |
|  | Highest vs. lowest total long-chain n-3 polyunsaturated fatty acid | F,M; Healthy | EU,NA,  AS | 3 y to  20 y | 25 to 84 | HTN | 7/7 | 16,896/  40,010 | RR | 0.73 (0.60, 0.89)* | 75% | Very Low |  |  |
| ***Diet exposure: Minerals*** | | | | | | | | | | | | | | |
| Han, 2017 (Han et al., 2017)  (prospective cohort) | Highest vs. lowest dietary magnesium intake | F,M; Healthy | EU,NA,  SA | 4 y to  15 y | 18 to 84 | HTN | 8/8 | 20,119/  180,566 | RR | 0.92 (0.86, 0.98)* | 0% | Very Low | NOS | Moderate |
|  | An increase of magnesium intake by 100 mg/d |  |  |  |  | HTN | 6/7 |  | RR | 0.95 (0.90, 1.00) | 39% | N/A |  |  |
|  | Highest vs. lowest serum magnesium level | F,M; Healthy | EU,NA,  SA | 4 y to  15 y | 18 to 84 | HTN | 4/4 | 20,119/  180,566 | RR | 0.91 (0.80, 1.02) | 0% | Very Low |  |  |
| Jayedi, 2019 (Jayedi & Zargar, 2019)  (prospective cohort) | Highest vs. lowest dietary and supplemental calcium intake | F,M; Healthy, some with HTN | EU,NA,  AS | 3.4 y to 10 y | 36 to 52 (m) | HTN | 8/8 | 30,838/  248,398 | RR | 0.89 (0.86, 0.93)* | 0% | Low | NOS,  NG | Moderate |
|  | An increase of dietary and supplemental calcium intake by 500 mg/d |  |  |  |  | HTN | 7/7 | 30,658/  242,518 | RR | 0.93 (0.90, 0.97)* | 64% | N/A |  |  |
| **Author, Year (Refence)** | **Comparison** | **Population** | **Regions** | **Study duration** | **Age range (mean)** | **Outcome** | **No. of primary studies/**  **estimates** | **No. of**  **cases/**  **participants** |  | **BP Change (95%CI)*** | **I^2^** | **NutriGrade** | **Quality**  **tool** | **AMSTAR** |
| Kuruppu, 2014 (Kuruppu et al., 2014)  (cross-sectional) | Correlation between mean plasma selenium level and SBP | F,M; Healthy, some with O | EU,NA,  AS,AU,  ME | NR | 18 to >70 | SBP | 13/13 | 10,731  (total) | Regression coefficient | 0.02 (0.00, 0.04)* | NR | Very Low | NR | Critically Low |
| Ziaei, 2020  (Ziaei, 2020)  (cross-sectional, cohort studies) | Correlation between urinary potassium and SBP | F,M; Healthy, | EU,AF,  AS | 7.9 y | 35 to 64 (m) | SBP | 7/9 | 6,965  (total) | Regression coefficient | -0.03 (-0.08, 0.02) | 70% | Very Low | NOS | Low |
| ***Diet exposure: Vitamins*** | | | | | | | | | | | | | | |
| Kunutsor, 2013 (Kunutsor et al., 2013)  (prospective studies) | Highest vs. lowest blood 25-hydroxyvitamin D level | F,M; Healthy | EU,NA | 1.3 y to 14 y | 18 to >70 | HTN | 7/7 | 4,965/  48,633 | RR | 0.70 (0.58, 0.86)* | NR | Low | NOS | Moderate |
|  | An increase of blood 25-hydroxyvitamin D level by 10 mg/ml |  |  |  |  | HTN | 5/5 | 2,371/  6,716 | RR | 0.88 (0.81, 0.97)* | NR | N/A |  |  |
|  | Highest vs. lowest dietary vitamin D intake | F,M; Healthy | NA | 6.9 y to 14 y | 18 to 74 | HTN | 4/4 | 52,000/  238,199 | RR | 1.00 (0.95, 1.05) | NR | Very Low |  |  |
| Ran, 2020 (Ran, 2020) | Correlation between serum vitamin C concentration and DBP | F,M; Healthy, some with HTN | EU,NA,  AS | NR | 19 to 80 | DBP | 10/11 | 22,200 (total) | Regression coefficient  Fisher’s Z | -0.15 (-0.20, -0.10) | 51% | Low | NOS | Low |
|  | Correlation between serum vitamin C concentration and SBP |  |  |  |  | SBP | 11/12 | 22,200 (total) | Regression coefficient  Fisher’s Z | -0.17 (-0.20, -0.15) | 37% | Low |  |  |
| **Author, Year (Refence)** | **Comparison** | **Population** | **Regions** | **Study duration** | **Age range (mean)** | **Outcome** | **No. of primary studies/**  **estimates** | **No. of**  **cases/**  **participants** |  | **BP Change (95%CI)*** | **I^2^** | **NutriGrade** | **Quality**  **tool** | **AMSTAR** |
| ***Diet exposure: Polyphenols*** | | | | | | | | | | | | | | |
| Godos, 2019 (Godos et al., 2019)  (cross-sectional, prospective cohort) | Highest vs. lowest anthocyanins intake | F,M; Healthy, some with O | EU,NA | 4 y to to 14 y | 18 to 74 | HTN | 3/6 | 45,732/  200,256 | RR | 0.92 (0.88, 0.97)* | 39% | Very Low | NOS | Low |
|  | Highest vs. lowest flavan-3-ol intake | F,M; Healthy, some with O | EU,NA | 4 y to to 14 y | 18 to 74 | HTN | 3/6 | 45,732/  200,256 | RR | 0.99 (0.92, 1.07) | 73% | Low |  |  |
|  | Highest vs. lowest flavanones intake | F,M; Healthy, some with O | EU,NA | 4 y to to 14 y | 18 to 74 | HTN | 3/6 | 45,732/  200,256 | RR | 0.98 (0.92, 1.04) | 61% | Low |  |  |
|  | Highest vs. lowest flavones intake | F,M; Healthy, some with O | EU,NA | 4 y to to 14 y | 18 to 87 | HTN | 3/6 | 45,732/  200,256 | RR | 0.96 (0.90, 1.02) | 56% | Very Low |  |  |
|  | Highest vs. lowest flavonols intake | F,M; Healthy, some with O | EU,NA | 4 y to to 14 y | 18 to 74 | HTN | 3/6 | 45,732/  200,256 | RR | 0.98 (0.91, 1.07) | 77% | Very Low |  |  |
|  | Highest vs. lowest total flavonoids intake | F,M; Healthy, some with O | EU,NA | 4 y to to 14 y | 18 to 87 | HTN | 3/6 | 45,732/  200,256 | RR | 0.96 (0.89, 1.03) | 74% | Very Low |  |  |

AF: Africa, AS: Asia, AU: Australia and New Zealand, BP: Blood pressure, CI: Confidence interval, d: day, DBP: Diastolic blood pressure, DM: Diabetes mellitus, EU: Europe, F: Female, h: hour, HTN: Hypertension, M: Males, m: month, ME: Middle East, MetS: Metabolic symptom, N/A: Not applicable, NA: North America, NR: Not reported, O: Other medical conditions, Ob: Obese, Ow: Overweight, RR: Relative risk, SA: South America, SBP: Systolic blood pressure, w: week, y: year

**References**

AbuMweis, S., Jew, S., Tayyem, R., & Agraib, L. (2018, Feb). Eicosapentaenoic acid and docosahexaenoic acid containing supplements modulate risk factors for cardiovascular disease: a meta-analysis of randomised placebo-control human clinical trials. *J Hum Nutr Diet, 31*(1), 67-84. <https://doi.org/10.1111/jhn.12493>

Aburto, N. J., Hanson, S., Gutierrez, H., Hooper, L., Elliott, P., & Cappuccio, F. P. (2013, Apr 3). Effect of increased potassium intake on cardiovascular risk factors and disease: systematic review and meta-analyses. *BMJ, 346*, f1378. <https://doi.org/10.1136/bmj.f1378>

Akbari, M., Tamtaji, O. R., Lankarani, K. B., Tabrizi, R., Dadgostar, E., Kolahdooz, F., Jamilian, M., Mirzaei, H., & Asemi, Z. (2019, Aug). The Effects of Resveratrol Supplementation on Endothelial Function and Blood Pressures Among Patients with Metabolic Syndrome and Related Disorders: A Systematic Review and Meta-Analysis of Randomized Controlled Trials. *High Blood Press Cardiovasc Prev, 26*(4), 305-319. <https://doi.org/10.1007/s40292-019-00324-6>

Alhassan, A., Young, J., Lean, M. E. J., & Lara, J. (2017, Nov). Consumption of fish and vascular risk factors: A systematic review and meta-analysis of intervention studies. *Atherosclerosis, 266*, 87-94. <https://doi.org/10.1016/j.atherosclerosis.2017.09.028>

Allender, P. S., Cutler, J. A., Follmann, D., Cappuccio, F. P., Pryer, J., & Elliott, P. (1996, May 1). Dietary calcium and blood pressure: a meta-analysis of randomized clinical trials. *Ann Intern Med, 124*(9), 825-831. <https://doi.org/10.7326/0003-4819-124-9-199605010-00007>

Amerizadeh, A. A., S. Vaseghi, G. Farajzadegan, Z. (2021, May 29). Effect of Genistein Intake on Some Cardiovascular Risk Factors: An Updated Systematic Review and Meta-analysis. *Curr Probl Cardiol*, 100902. <https://doi.org/10.1016/j.cpcardiol.2021.100902> UpdatedTo31Oct2021

Asbaghi, O., Hadi, A., Campbell, M. S., Venkatakrishnan, K., & Ghaedi, E. (2021, Sep 14). Effects of pistachios on anthropometric indices, inflammatory markers, endothelial function and blood pressure in adults: a systematic review and meta-analysis of randomised controlled trials. *Br J Nutr, 126*(5), 718-729. <https://doi.org/10.1017/s0007114520004523> UpdatedTo31Oct2021

Asbaghi, O., Naeini, F., Moodi, V., Najafi, M., Shirinbakhshmasoleh, M., Kelishadi, M. R., Hadi, A., Ghaedi, E., & Fadel, A. (2021, Jan). Effect of grape products on blood pressure: a systematic review and meta-analysis of randomized controlled trials [Review]. *International Journal of Food Properties, 24*(1), 627-645. <https://doi.org/10.1080/10942912.2021.1901731> UpdatedTo31Oct2021

Asbaghi, O. S., S. Rezaei Kelishadi, M. Bagheri, R. Ashtary-Larky, D. Nazarian, B. Mombaini, D. Ghanavati, M. Clark, C. C. T. Wong, A. Naeini, A. A. (2021, Sep 3). Folic acid supplementation and blood pressure: a GRADE-assessed systematic review and dose-response meta-analysis of 41,633 participants. *Crit Rev Food Sci Nutr*, 1-16. <https://doi.org/10.1080/10408398.2021.1968787> UpdatedTo31Oct2021

Ashtary-Larky, D. R. K., M. Bagheri, R. Moosavian, S. P. Wong, A. Davoodi, S. H. Khalili, P. Dutheil, F. Suzuki, K. Asbaghi, O. (2021, Jun 24). The Effects of Nano-Curcumin Supplementation on Risk Factors for Cardiovascular Disease: A GRADE-Assessed Systematic Review and Meta-Analysis of Clinical Trials. *Antioxidants (Basel), 10*(7). <https://doi.org/10.3390/antiox10071015> UpdatedTo31Oct2021

Askarpour, M., Hadi, A., Dehghani Kari Bozorg, A., Sadeghi, O., Sheikhi, A., Kazemi, M., & Ghaedi, E. (2019, Oct). Effects of L-carnitine supplementation on blood pressure: a systematic review and meta-analysis of randomized controlled trials. *J Hum Hypertens, 33*(10), 725-734. <https://doi.org/10.1038/s41371-019-0248-1>

Azad, M. B., Abou-Setta, A. M., Chauhan, B. F., Rabbani, R., Lys, J., Copstein, L., Mann, A., Jeyaraman, M. M., Reid, A. E., Fiander, M., MacKay, D. S., McGavock, J., Wicklow, B., & Zarychanski, R. (2017, Jul 17). Nonnutritive sweeteners and cardiometabolic health: a systematic review and meta-analysis of randomized controlled trials and prospective cohort studies. *CMAJ, 189*(28), E929-E939. <https://doi.org/10.1503/cmaj.161390>

Badely, M., Sepandi, M., Samadi, M., Parastouei, K., & Taghdir, M. (2019, Nov - Dec). The effect of whey protein on the components of metabolic syndrome in overweight and obese individuals; a systematic review and meta-analysis. *Diabetes Metab Syndr, 13*(6), 3121-3131. <https://doi.org/10.1016/j.dsx.2019.11.001>

Bahadoran, Z., Mirmiran, P., Kabir, A., Azizi, F., & Ghasemi, A. (2017, Nov). The Nitrate-Independent Blood Pressure-Lowering Effect of Beetroot Juice: A Systematic Review and Meta-Analysis. *Adv Nutr, 8*(6), 830-838. <https://doi.org/10.3945/an.117.016717>

Baye, E., Kiriakova, V., Uribarri, J., Moran, L. J., & de Courten, B. (2017, May 23). Consumption of diets with low advanced glycation end products improves cardiometabolic parameters: meta-analysis of randomised controlled trials. *Sci Rep, 7*(1), 2266. <https://doi.org/10.1038/s41598-017-02268-0>

Benatar, J. R., Sidhu, K., & Stewart, R. A. (2013). Effects of high and low fat dairy food on cardio-metabolic risk factors: a meta-analysis of randomized studies. *PLoS One, 8*(10), e76480. <https://doi.org/10.1371/journal.pone.0076480>

Benatar, J. R., & Stewart, R. A. H. (2018, Dec 20). Cardiometabolic risk factors in vegans; A meta-analysis of observational studies. *PLoS One, 13*(12), e0209086. <https://doi.org/10.1371/journal.pone.0209086>

Beyer, F. R., Dickinson, H. O., Nicolson, D., Ford, G. A., & Mason, J. (2006). Combined calcium, magnesium and potassium supplementation for the management of primary hypertension in adults. *Cochrane Database of Systematic Reviews*(3). <https://doi.org/10.1002/14651858.CD004805.pub2>

Blanco Mejia, S., Kendall, C. W., Viguiliouk, E., Augustin, L. S., Ha, V., Cozma, A. I., Mirrahimi, A., Maroleanu, A., Chiavaroli, L., Leiter, L. A., de Souza, R. J., Jenkins, D. J., & Sievenpiper, J. L. (2014, Jul 29). Effect of tree nuts on metabolic syndrome criteria: a systematic review and meta-analysis of randomised controlled trials. *BMJ Open, 4*(7), e004660. <https://doi.org/10.1136/bmjopen-2013-004660>

Bonnet, J. P. C., M. I. Cellini, J. Hu, F. B. Guasch-Ferré, M. (2020, Jun). Breakfast Skipping, Body Composition, and Cardiometabolic Risk: A Systematic Review and Meta-Analysis of Randomized Trials. *Obesity (Silver Spring), 28*(6), 1098-1109. <https://doi.org/10.1002/oby.22791> UpdatedTo31Oct2021

Bueno, N. B., de Melo, I. S., de Oliveira, S. L., & da Rocha Ataide, T. (2013, Oct). Very-low-carbohydrate ketogenic diet v. low-fat diet for long-term weight loss: a meta-analysis of randomised controlled trials. *Br J Nutr, 110*(7), 1178-1187. <https://doi.org/10.1017/S0007114513000548>

Carvalho, M. F. L., A. B. A. Ribeiro, E. Silva V. R. Macedo, L. R. Silva, M. (2021, Jul). Blueberry intervention improves metabolic syndrome risk factors: systematic review and meta-analysis. *Nutr Res, 91*, 67-80. <https://doi.org/10.1016/j.nutres.2021.04.006> UpdatedTo31Oct2021

Chiavaroli, L., Nishi, S. K., Khan, T. A., Braunstein, C. R., Glenn, A. J., Mejia, S. B., Rahelic, D., Kahleova, H., Salas-Salvado, J., Jenkins, D. J. A., Kendall, C. W. C., & Sievenpiper, J. L. (2018, May - Jun). Portfolio Dietary Pattern and Cardiovascular Disease: A Systematic Review and Meta-analysis of Controlled Trials. *Prog Cardiovasc Dis, 61*(1), 43-53. <https://doi.org/10.1016/j.pcad.2018.05.004>

Clark, C. C. T. S., M. Aghabagheri, E. Jafarnejad, S. (2020, Nov). The effect of psyllium supplementation on blood pressure: a systematic review and meta-analysis of randomized controlled trials. *Korean J Intern Med, 35*(6), 1385-1399. <https://doi.org/10.3904/kjim.2019.049> UpdatedTo31Oct2021

D'Elia, L. D., M. Sofi, F. Volpe, M. Strazzullo, P. (2021, Aug). 100% Fruit juice intake and cardiovascular risk: a systematic review and meta-analysis of prospective and randomised controlled studies. *Eur J Nutr, 60*(5), 2449-2467. <https://doi.org/10.1007/s00394-020-02426-7> UpdatedTo31Oct2021

Daneshzad, E., Shab-Bidar, S., Mohammadpour, Z., & Djafarian, K. (2019, Jun). Effect of anthocyanin supplementation on cardio-metabolic biomarkers: A systematic review and meta-analysis of randomized controlled trials. *Clin Nutr, 38*(3), 1153-1165. <https://doi.org/10.1016/j.clnu.2018.06.979>

Dehghan, P., & Abbasalizad Farhangi, M. (2020, Apr). Dietary acid load, blood pressure, fasting blood sugar and biomarkers of insulin resistance among adults: Findings from an updated systematic review and meta-analysis. *Int J Clin Pract, 74*(4), e13471. <https://doi.org/10.1111/ijcp.13471>

Dong, J. Y., Tong, X., Wu, Z. W., Xun, P. C., He, K., & Qin, L. Q. (2011, Aug). Effect of soya protein on blood pressure: a meta-analysis of randomised controlled trials. *Br J Nutr, 106*(3), 317-326. <https://doi.org/10.1017/S0007114511000262>

Dong, T. G., M. Zhang, P. Sun, G. Chen, B. (2020). The effects of low-carbohydrate diets on cardiovascular risk factors: A meta-analysis. *PLoS One, 15*(1), e0225348. <https://doi.org/10.1371/journal.pone.0225348> UpdatedTo31Oct2021

Ellwood, L., Torun, G., Bahar, Z., & Fernandez, R. (2019, Oct). Effects of flavonoid-rich fruits on hypertension in adults: a systematic review. *JBI Database System Rev Implement Rep, 17*(10), 2075-2105. <https://doi.org/10.11124/JBISRIR-D-19-00050>

Emami, M. R., Safabakhsh, M., Alizadeh, S., Asbaghi, O., & Khosroshahi, M. Z. (2019, Jul). Effect of vitamin E supplementation on blood pressure: a systematic review and meta-analysis. *J Hum Hypertens, 33*(7), 499-507. <https://doi.org/10.1038/s41371-019-0192-0>

Eslampour, E. A., O. Hadi, A. Abedi, S. Ghaedi, E. Lazaridi, A. V. Miraghajani, M. (2020, May). The effect of almond intake on blood pressure: A systematic review and meta-analysis of randomized controlled trials. *Complement Ther Med, 50*, 102399. <https://doi.org/10.1016/j.ctim.2020.102399> UpdatedTo31Oct2021

Evans, C. E., Greenwood, D. C., Threapleton, D. E., Cleghorn, C. L., Nykjaer, C., Woodhead, C. E., Gale, C. P., & Burley, V. J. (2015, May). Effects of dietary fibre type on blood pressure: a systematic review and meta-analysis of randomized controlled trials of healthy individuals. *J Hypertens, 33*(5), 897-911. <https://doi.org/10.1097/HJH.0000000000000515>

Faghihimani, Z. N., N. Ghaffari, S. Kelishadi, M. R. Sharifi, S. Nattagh-Eshtivani, E. Akbarzadeh, M. Moravejolahkami, A. R. Khorvash, F. Roshanravan, N. Alamdari, N. M. (2021, Jan). Effects of Inulin Type-Carbohydrates on blood pressure: a systematic review and meta-analysis [Review]. *International Journal of Food Properties, 24*(1), 129-139. <https://doi.org/10.1080/10942912.2020.1858863> UpdatedTo31Oct2021

Farhangi, M. A., Nikniaz, L., Nikniaz, Z., & Dehghan, P. (2020, Jun). Dietary inflammatory index potentially increases blood pressure and markers of glucose homeostasis among adults: findings from an updated systematic review and meta-analysis. *Public Health Nutr, 23*(8), 1362-1380. <https://doi.org/10.1017/S1368980019003070>

Fekete, A. A., Givens, D. I., & Lovegrove, J. A. (2015, Jan 20). Casein-derived lactotripeptides reduce systolic and diastolic blood pressure in a meta-analysis of randomised clinical trials. *Nutrients, 7*(1), 659-681. <https://doi.org/10.3390/nu7010659>

Feringa, H. H., Laskey, D. A., Dickson, J. E., & Coleman, C. I. (2011, Aug). The effect of grape seed extract on cardiovascular risk markers: a meta-analysis of randomized controlled trials. *J Am Diet Assoc, 111*(8), 1173-1181. <https://doi.org/10.1016/j.jada.2011.05.015>

Filippou, C. D., Tsioufis, C. P., Thomopoulos, C. G., Mihas, C. C., Dimitriadis, K. S., Sotiropoulou, L. I., Chrysochoou, C. A., Nihoyannopoulos, P. I., & Tousoulis, D. M. (2020, Sep 1). Dietary Approaches to Stop Hypertension (DASH) Diet and Blood Pressure Reduction in Adults with and without Hypertension: A Systematic Review and Meta-Analysis of Randomized Controlled Trials. *Adv Nutr, 11*(5), 1150-1160. <https://doi.org/10.1093/advances/nmaa041>

Filippou, C. D. T., C. G. Kouremeti, M. M. Sotiropoulou, L. I. Nihoyannopoulos, P. I. Tousoulis, D. M. Tsioufis, C. P. (2021, May). Mediterranean diet and blood pressure reduction in adults with and without hypertension: A systematic review and meta-analysis of randomized controlled trials. *Clin Nutr, 40*(5), 3191-3200. <https://doi.org/10.1016/j.clnu.2021.01.030> UpdatedTo31Oct2021

Garcia-Conesa, M. T., Chambers, K., Combet, E., Pinto, P., Garcia-Aloy, M., Andres-Lacueva, C., de Pascual-Teresa, S., Mena, P., Konic Ristic, A., Hollands, W. J., Kroon, P. A., Rodriguez-Mateos, A., Istas, G., Kontogiorgis, C. A., Rai, D. K., Gibney, E. R., Morand, C., Espin, J. C., & Gonzalez-Sarrias, A. (2018, Feb 28). Meta-Analysis of the Effects of Foods and Derived Products Containing Ellagitannins and Anthocyanins on Cardiometabolic Biomarkers: Analysis of Factors Influencing Variability of the Individual Responses. *Int J Mol Sci, 19*(3). <https://doi.org/10.3390/ijms19030694>

Gay, H. C., Rao, S. G., Vaccarino, V., & Ali, M. K. (2016, Apr). Effects of Different Dietary Interventions on Blood Pressure: Systematic Review and Meta-Analysis of Randomized Controlled Trials. *Hypertension, 67*(4), 733-739. <https://doi.org/10.1161/HYPERTENSIONAHA.115.06853>

Geleijnse, J. M., Kok, F. J., & Grobbee, D. E. (2003, Jul). Blood pressure response to changes in sodium and potassium intake: a metaregression analysis of randomised trials. *J Hum Hypertens, 17*(7), 471-480. <https://doi.org/10.1038/sj.jhh.1001575>

Ghaedi, E., Mohammadi, M., Mohammadi, H., Ramezani-Jolfaie, N., Malekzadeh, J., Hosseinzadeh, M., & Salehi-Abargouei, A. (2019, Jul 1). Effects of a Paleolithic Diet on Cardiovascular Disease Risk Factors: A Systematic Review and Meta-Analysis of Randomized Controlled Trials. *Adv Nutr, 10*(4), 634-646. <https://doi.org/10.1093/advances/nmz007>

Ghaedi, E. F., S. Ziaei, R. Beigrezaei, S. Kord-Varkaneh, H. Ghavami, A. Miraghajani, M. (2020, Sep). Effects of phytosterols supplementation on blood pressure: A systematic review and meta-analysis. *Clin Nutr, 39*(9), 2702-2710. <https://doi.org/10.1016/j.clnu.2019.12.020> UpdatedTo31Oct2021

Ghanbari, M. A., M. R. Djafarian, K. Shab-Bidar, S. (2021, Jul 23). The effects of chromium supplementation on blood pressure: a systematic review and meta-analysis of randomized clinical trials. *Eur J Clin Nutr*. <https://doi.org/10.1038/s41430-021-00973-8> UpdatedTo31Oct2021

Ghavami, A. Z., R. Moradi, S. Sharifi, S. Moravejolahkami, A. R. Ghaffari, S. Irandoost, P. Khorvash, F. Mokari-Yamchi, A. Nattagh-Eshtivani, E. Roshanravan, N. (2020, Jan). Potential of favorable effects of probiotics fermented milk supplementation on blood pressure: a systematic review and meta-analysis [Review]. *International Journal of Food Properties, 23*(1), 1925-1940. <https://doi.org/10.1080/10942912.2020.1833030> UpdatedTo31Oct2021

Gibbs, J. G., E. Ji, C. Miller, M. A. Cappuccio, F. P. (2021, Jan). The effect of plant-based dietary patterns on blood pressure: a systematic review and meta-analysis of controlled intervention trials. *J Hypertens, 39*(1), 23-37. <https://doi.org/10.1097/hjh.0000000000002604> UpdatedTo31Oct2021

Godos, J., Vitale, M., Micek, A., Ray, S., Martini, D., Del Rio, D., Riccardi, G., Galvano, F., & Grosso, G. (2019, May 31). Dietary Polyphenol Intake, Blood Pressure, and Hypertension: A Systematic Review and Meta-Analysis of Observational Studies. *Antioxidants (Basel), 8*(6). <https://doi.org/10.3390/antiox8060152>

Godos, J., Zappala, G., Bernardini, S., Giambini, I., Bes-Rastrollo, M., & Martinez-Gonzalez, M. (2017, Mar). Adherence to the Mediterranean diet is inversely associated with metabolic syndrome occurrence: a meta-analysis of observational studies. *Int J Food Sci Nutr, 68*(2), 138-148. <https://doi.org/10.1080/09637486.2016.1221900>

Golzarand, M., Shab-Bidar, S., Koochakpoor, G., Speakman, J. R., & Djafarian, K. (2016, Aug). Effect of vitamin D3 supplementation on blood pressure in adults: An updated meta-analysis. *Nutr Metab Cardiovasc Dis, 26*(8), 663-673. <https://doi.org/10.1016/j.numecd.2016.04.011>

Griffith, L. E., Guyatt, G. H., Cook, R. J., Bucher, H. C., & Cook, D. J. (1999, Jan). The influence of dietary and nondietary calcium supplementation on blood pressure: an updated metaanalysis of randomized controlled trials. *Am J Hypertens, 12*(1 Pt 1), 84-92. <https://doi.org/10.1016/s0895-7061(98)00224-6>

Guan, L. M., P. (2020, Oct 15). The effects of taurine supplementation on obesity, blood pressure and lipid profile: A meta-analysis of randomized controlled trials. *Eur J Pharmacol, 885*, 173533. <https://doi.org/10.1016/j.ejphar.2020.173533> UpdatedTo31Oct2021

Guo, X. F., Li, K. L., Li, J. M., & Li, D. (2019). Effects of EPA and DHA on blood pressure and inflammatory factors: a meta-analysis of randomized controlled trials. *Crit Rev Food Sci Nutr, 59*(20), 3380-3393. <https://doi.org/10.1080/10408398.2018.1492901>

Guo, X. F., Li, Z. H., Cai, H., & Li, D. (2017, May 24). The effects of Lycium barbarum L. (L. barbarum) on cardiometabolic risk factors: a meta-analysis of randomized controlled trials. *Food Funct, 8*(5), 1741-1748. <https://doi.org/10.1039/c7fo00183e>

Ha, V., Sievenpiper, J. L., de Souza, R. J., Chiavaroli, L., Wang, D. D., Cozma, A. I., Mirrahimi, A., Yu, M. E., Carleton, A. J., Dibuono, M., Jenkins, A. L., Leiter, L. A., Wolever, T. M., Beyene, J., Kendall, C. W., & Jenkins, D. J. (2012, Apr). Effect of fructose on blood pressure: a systematic review and meta-analysis of controlled feeding trials. *Hypertension, 59*(4), 787-795. <https://doi.org/10.1161/HYPERTENSIONAHA.111.182311>

Hadi, A., Askarpour, M., Miraghajani, M., Symonds, M. E., Sheikhi, A., & Ghaedi, E. (2019, Nov 1). Effects of strawberry supplementation on cardiovascular risk factors: a comprehensive systematic review and meta-analysis of randomized controlled trials. *Food Funct, 10*(11), 6987-6998. <https://doi.org/10.1039/c9fo01684h>

Han, B., Nazary-Vannani, A., Talaei, S., Clark, C. C. T., Rahmani, J., Rasekhmagham, R., & Kord-Varkaneh, H. (2019, Nov). The effect of green coffee extract supplementation on blood pressure: A systematic review and meta-analysis of randomized controlled trials. *Phytother Res, 33*(11), 2918-2926. <https://doi.org/10.1002/ptr.6481>

Han, B. B., A. S. Rashid, M. Chhabra, M. Clark, C. Abdulazeem, H. M. Abd-ElGawad, M. Varkaneh, H. K. Rahmani, J. Zhang, Y. (2020, Mar). The effect of sour cherry consumption on blood pressure, IL-6, CRP, and TNF-alpha levels: A systematic review and meta-analysis of randomized controlled trials sour cherry consumption and blood pressure [Review]. *Journal of King Saud University Science, 32*(2), 1687-1693. <https://doi.org/10.1016/j.jksus.2020.01.002> UpdatedTo31Oct2021

Han, H., Fang, X., Wei, X., Liu, Y., Jin, Z., Chen, Q., Fan, Z., Aaseth, J., Hiyoshi, A., He, J., & Cao, Y. (2017, May 5). Dose-response relationship between dietary magnesium intake, serum magnesium concentration and risk of hypertension: a systematic review and meta-analysis of prospective cohort studies. *Nutr J, 16*(1), 26. <https://doi.org/10.1186/s12937-017-0247-4>

Hasani, H., Arab, A., Hadi, A., Pourmasoumi, M., Ghavami, A., & Miraghajani, M. (2019, Jun). Does ginger supplementation lower blood pressure? A systematic review and meta-analysis of clinical trials. *Phytother Res, 33*(6), 1639-1647. <https://doi.org/10.1002/ptr.6362>

Hashemi Tari, S. S., M. H. Lari, A. Fatahi, S. Rahideh, S. T. (2021, Aug). The effect of inositol supplementation on blood pressure: A systematic review and meta-analysis of randomized-controlled trials. *Clin Nutr ESPEN, 44*, 78-84. <https://doi.org/10.1016/j.clnesp.2021.06.017> UpdatedTo31Oct2021

Hawkins, J., Hires, C., Baker, C., Keenan, L., & Bush, M. (2021). Daily supplementation with aronia melanocarpa (chokeberry) reduces blood pressure and cholesterol: a meta analysis of controlled clinical trials. *J Diet Suppl, 18*(5), 517-530. <https://doi.org/10.1080/19390211.2020.1800887>

He, Y. L., J. Cai, H. Zhang, J. Yi, J. Niu, Y. Xi, H. Peng, X. Guo, L. (2021, Sep 1). Effect of inorganic nitrate supplementation on blood pressure in older adults: A systematic review and meta-analysis. *Nitric Oxide, 113-114*, 13-22. <https://doi.org/10.1016/j.niox.2021.04.006> UpdatedTo31Oct2021

Heidari, Z. R. P. F., N. Clark, C. C. T. Haghighatdoost, F. (2021, Jun 30). Dairy products consumption and the risk of hypertension in adults: An updated systematic review and dose-response meta-analysis of prospective cohort studies. *Nutr Metab Cardiovasc Dis, 31*(7), 1962-1975. <https://doi.org/10.1016/j.numecd.2021.02.033> UpdatedTo31Oct2021

Hernandez, A. V., Emonds, E. E., Chen, B. A., Zavala-Loayza, A. J., Thota, P., Pasupuleti, V., Roman, Y. M., Bernabe-Ortiz, A., & Miranda, J. J. (2019, Jun). Effect of low-sodium salt substitutes on blood pressure, detected hypertension, stroke and mortality. *Heart, 105*(12), 953-960. <https://doi.org/10.1136/heartjnl-2018-314036>

Hidayat, K., Du, H. Z., Yang, J., Chen, G. C., Zhang, Z., Li, Z. N., & Qin, L. Q. (2017, Mar). Effects of milk proteins on blood pressure: a meta-analysis of randomized control trials. *Hypertens Res, 40*(3), 264-270. <https://doi.org/10.1038/hr.2016.135>

Hohmann, C. D., Cramer, H., Michalsen, A., Kessler, C., Steckhan, N., Choi, K., & Dobos, G. (2015, Jun 1). Effects of high phenolic olive oil on cardiovascular risk factors: A systematic review and meta-analysis. *Phytomedicine, 22*(6), 631-640. <https://doi.org/10.1016/j.phymed.2015.03.019>

Hu, T., Mills, K. T., Yao, L., Demanelis, K., Eloustaz, M., Yancy, W. S., Jr, Kelly, T. N., He, J., & Bazzano, L. A. (2012). Effects of Low-Carbohydrate Diets Versus Low-Fat Diets on Metabolic Risk Factors: A Meta-Analysis of Randomized Controlled Clinical Trials. *American Journal of Epidemiology, 176*(suppl_7), S44-S54. <https://doi.org/10.1093/aje/kws264>

Huang, H. Z., G. Pu, R. Cui, Y. Liao, D. (2021, Feb 22). Clinical evidence of dietary supplementation with sesame on cardiovascular risk factors: An updated meta-analysis of randomized controlled trials. *Crit Rev Food Sci Nutr*, 1-11. <https://doi.org/10.1080/10408398.2021.1888689> UpdatedTo31Oct2021

Ismail, M. A. N., M. N. Mohamad, N. (2021). Olive leaf extract effect on cardiometabolic profile among adults with prehypertension and hypertension: a systematic review and meta-analysis. *PeerJ, 9*, e11173. <https://doi.org/10.7717/peerj.11173> UpdatedTo31Oct2021

Jalali, M. K., M. Ferns, G. A. Zare, M. Moosavian, S. P. Akbarzadeh, M. (2020, May). The effects of cashew nut intake on lipid profile and blood pressure: A systematic review and meta-analysis of randomized controlled trials. *Complement Ther Med, 50*, 102387. <https://doi.org/10.1016/j.ctim.2020.102387> UpdatedTo31Oct2021

Jandari, S. G., A. Ziaei, R. Nattagh-Eshtivani, E. Kelishadi, M. R. Sharifi, S. Khorvash, F. Pahlavani, N. Mohammadi, H. (2020, Jan). Effects of Momordica charantia L on blood pressure: a systematic review and meta- analysis of randomized clinical trials [Review]. *International Journal of Food Properties, 23*(1), 1913-1924. <https://doi.org/10.1080/10942912.2020.1833916> UpdatedTo31Oct2021

Jayalath, V. H., de Souza, R. J., Sievenpiper, J. L., Ha, V., Chiavaroli, L., Mirrahimi, A., Di Buono, M., Bernstein, A. M., Leiter, L. A., Kris-Etherton, P. M., Vuksan, V., Beyene, J., Kendall, C. W., & Jenkins, D. J. (2014, Jan). Effect of dietary pulses on blood pressure: a systematic review and meta-analysis of controlled feeding trials. *Am J Hypertens, 27*(1), 56-64. <https://doi.org/10.1093/ajh/hpt155>

Jayalath, V. H., Sievenpiper, J. L., de Souza, R. J., Ha, V., Mirrahimi, A., Santaren, I. D., Blanco Mejia, S., Di Buono, M., Jenkins, A. L., Leiter, L. A., Wolever, T. M., Beyene, J., Kendall, C. W., & Jenkins, D. J. (2014, 2014). Total fructose intake and risk of hypertension: a systematic review and meta-analysis of prospective cohorts. *J Am Coll Nutr, 33*(4), 328-339. <https://doi.org/10.1080/07315724.2014.916237>

Jayedi, A., & Zargar, M. S. (2019, Jul). Dietary calcium intake and hypertension risk: a dose-response meta-analysis of prospective cohort studies. *Eur J Clin Nutr, 73*(7), 969-978. <https://doi.org/10.1038/s41430-018-0275-y>

Juraschek, S. P., Guallar, E., Appel, L. J., & Miller, E. R., 3rd. (2012, May). Effects of vitamin C supplementation on blood pressure: a meta-analysis of randomized controlled trials. *Am J Clin Nutr, 95*(5), 1079-1088. <https://doi.org/10.3945/ajcn.111.027995>

Kelly, S. A., Hartley, L., Loveman, E., Colquitt, J. L., Jones, H. M., Al-Khudairy, L., Clar, C., Germano, R., Lunn, H. R., Frost, G., & Rees, K. (2017, Aug 24). Whole grain cereals for the primary or secondary prevention of cardiovascular disease. *Cochrane Database Syst Rev, 8*, CD005051. <https://doi.org/10.1002/14651858.CD005051.pub3>

Khan, K., Jovanovski, E., Ho, H. V. T., Marques, A. C. R., Zurbau, A., Mejia, S. B., Sievenpiper, J. L., & Vuksan, V. (2018, Jan). The effect of viscous soluble fiber on blood pressure: A systematic review and meta-analysis of randomized controlled trials. *Nutr Metab Cardiovasc Dis, 28*(1), 3-13. <https://doi.org/10.1016/j.numecd.2017.09.007>

Khazdouz, M., Djalalinia, S., Sarrafi Zadeh, S., Hasani, M., Shidfar, F., Ataie-Jafari, A., Asayesh, H., Zarei, M., Gorabi, A. M., Noroozi, M., & Qorbani, M. (2020, Jun). Effects of Zinc Supplementation on Cardiometabolic Risk Factors: a Systematic Review and Meta-analysis of Randomized Controlled Trials. *Biol Trace Elem Res, 195*(2), 373-398. <https://doi.org/10.1007/s12011-019-01870-9>

Kim, Y., & Je, Y. (2016, Apr). Prospective association of sugar-sweetened and artificially sweetened beverage intake with risk of hypertension. *Arch Cardiovasc Dis, 109*(4), 242-253. <https://doi.org/10.1016/j.acvd.2015.10.005>

Kirkham, A. A. B., V. Prado, C. M. (2021, Mar). The effect of caloric restriction on blood pressure and cardiovascular function: A systematic review and meta-analysis of randomized controlled trials. *Clin Nutr, 40*(3), 728-739. <https://doi.org/10.1016/j.clnu.2020.06.029> UpdatedTo31Oct2021

Kolahdouz-Mohammadi, R. M., M. Clayton, Z. S. Sadat, S. Z. Pahlavani, N. Sikaroudi, M. K. Soltani, S. (2020, Feb 29). Effect of Egg Consumption on Blood Pressure: a Systematic Review and Meta-analysis of Randomized Clinical Trials. *Curr Hypertens Rep, 22*(3), 24. <https://doi.org/10.1007/s11906-020-1029-5> UpdatedTo31Oct2021

Kunutsor, S. K., Apekey, T. A., & Steur, M. (2013, Mar). Vitamin D and risk of future hypertension: meta-analysis of 283,537 participants. *Eur J Epidemiol, 28*(3), 205-221. <https://doi.org/10.1007/s10654-013-9790-2>

Kuruppu, D., Hendrie, H. C., Yang, L., & Gao, S. (2014, Jun). Selenium levels and hypertension: a systematic review of the literature. *Public Health Nutr, 17*(6), 1342-1352. <https://doi.org/10.1017/S1368980013000992>

Lee, K. W. L., H. C. Ching, S. M. Devaraj, N. K. Hoo, F. K. (2020, May 29). Effects of Vegetarian Diets on Blood Pressure Lowering: A Systematic Review with Meta-Analysis and Trial Sequential Analysis. *Nutrients, 12*(6). <https://doi.org/10.3390/nu12061604> UpdatedTo31Oct2021

Lee, M., Lee, H., & Kim, J. (2018, Aug). Dairy food consumption is associated with a lower risk of the metabolic syndrome and its components: a systematic review and meta-analysis. *Br J Nutr, 120*(4), 373-384. <https://doi.org/10.1017/S0007114518001460>

Lewis-Mikhael, A. M. D., A. Jafarnejad, S. (2020, Mar). Effect of Lactobacillusplantarum containing probiotics on blood pressure: A systematic review and meta-analysis. *Pharmacol Res, 153*, 104663. <https://doi.org/10.1016/j.phrs.2020.104663> UpdatedTo31Oct2021

Li, J. J., B. O. Santos H Santos, D. Singh, A. Wang, L. (2020, Nov). Effects of walnut intake on blood pressure: A systematic review and meta-analysis of randomized controlled trials. *Phytother Res, 34*(11), 2921-2931. <https://doi.org/10.1002/ptr.6740> UpdatedTo31Oct2021

Li, K., Liu, C., Kuang, X., Deng, Q., Zhao, F., & Li, D. (2018, Aug 3). Effects of Multivitamin and Multimineral Supplementation on Blood Pressure: A Meta-Analysis of 12 Randomized Controlled Trials. *Nutrients, 10*(8). <https://doi.org/10.3390/nu10081018>

Li, S. H., Zhao, P., Tian, H. B., Chen, L. H., & Cui, L. Q. (2015, 2015). Effect of Grape Polyphenols on Blood Pressure: A Meta-Analysis of Randomized Controlled Trials. *PLoS One, 10*(9), e0137665. <https://doi.org/10.1371/journal.pone.0137665>

Li, X., Wang, W., Hou, L., Wu, H., Wu, Y., Xu, R., Xiao, Y., & Wang, X. (2020, Apr). Does tea extract supplementation benefit metabolic syndrome and obesity? A systematic review and meta-analysis. *Clin Nutr, 39*(4), 1049-1058. <https://doi.org/10.1016/j.clnu.2019.05.019>

Li, X. H., Feng, L., Yang, Z. H., & Liao, Y. H. (2015, Oct). Effect of active vitamin D on cardiovascular outcomes in predialysis chronic kidney diseases: A systematic review and meta-analysis. *Nephrology (Carlton), 20*(10), 706-714. <https://doi.org/10.1111/nep.12505>

Liu, G., Zheng, X., Lu, J., & Huang, X. (2014, 2014). Effects of tea intake on blood pressure: A metaanalysis of 21 randomized controlled trials. *Cardiology (Switzerland), 129*, 10-11. <http://ovidsp.ovid.com/ovidweb.cgi?T=JS&PAGE=reference&D=emed15&NEWS=N&AN=71652284>

Liu, K., Xing, A., Chen, K., Wang, B., Zhou, R., Chen, S., Xu, H., & Mi, M. (2013, 2013). Effect of fruit juice on cholesterol and blood pressure in adults: a meta-analysis of 19 randomized controlled trials. *PLoS One, 8*(4), e61420. <https://doi.org/10.1371/journal.pone.0061420>

Liu, L., Ikeda, K., Sullivan, D. H., Ling, W., & Yamori, Y. (2002, Sep). Epidemiological evidence of the association between dietary protein intake and blood pressure: a meta-analysis of published data. *Hypertens Res, 25*(5), 689-695. <https://doi.org/10.1291/hypres.25.689>

Lopez, P. D., Cativo, E. H., Atlas, S. A., & Rosendorff, C. (2019, Jul). The Effect of Vegan Diets on Blood Pressure in Adults: A Meta-Analysis of Randomized Controlled Trials. *Am J Med, 132*(7), 875-883 e877. <https://doi.org/10.1016/j.amjmed.2019.01.044>

Luis, A., Domingues, F., & Pereira, L. (2018a, Feb 21). Association between berries intake and cardiovascular diseases risk factors: a systematic review with meta-analysis and trial sequential analysis of randomized controlled trials. *Food Funct, 9*(2), 740-757. <https://doi.org/10.1039/c7fo01551h>

Luis, A., Domingues, F., & Pereira, L. (2018b, Jan 15). Metabolic changes after licorice consumption: A systematic review with meta-analysis and trial sequential analysis of clinical trials. *Phytomedicine, 39*, 17-24. <https://doi.org/10.1016/j.phymed.2017.12.010>

Ma, C. Z., X. Yang, Y. Bu, P. (2021, Jan 7). The effect of black tea supplementation on blood pressure: a systematic review and dose-response meta-analysis of randomized controlled trials. *Food Funct, 12*(1), 41-56. <https://doi.org/10.1039/d0fo02122a> UpdatedTo31Oct2021

Mazzaro, C. C., Klostermann, F. C., Erbano, B. O., Schio, N. A., Guarita-Souza, L. C., Olandoski, M., Faria-Neto, J. R., & Baena, C. P. (2014, Apr). Dietary interventions and blood pressure in Latin America - systematic review and meta-analysis. *Arq Bras Cardiol, 102*(4), 345-354. <https://doi.org/10.5935/abc.20140037>

Menezes, R., Rodriguez-Mateos, A., Kaltsatou, A., Gonzalez-Sarrias, A., Greyling, A., Giannaki, C., Andres-Lacueva, C., Milenkovic, D., Gibney, E. R., Dumont, J., Schar, M., Garcia-Aloy, M., Palma-Duran, S. A., Ruskovska, T., Maksimova, V., Combet, E., & Pinto, P. (2017, Feb 9). Impact of Flavonols on Cardiometabolic Biomarkers: A Meta-Analysis of Randomized Controlled Human Trials to Explore the Role of Inter-Individual Variability. *Nutrients, 9*(2). <https://doi.org/10.3390/nu9020117>

Miller, P. E., Van Elswyk, M., & Alexander, D. D. (2014, Jul). Long-chain omega-3 fatty acids eicosapentaenoic acid and docosahexaenoic acid and blood pressure: a meta-analysis of randomized controlled trials. *Am J Hypertens, 27*(7), 885-896. <https://doi.org/10.1093/ajh/hpu024>

Mirenayat, M. S., Moradi, S., Mohammadi, H., & Rouhani, M. H. (2018, Oct 3). Effect of L-Citrulline Supplementation on Blood Pressure: a Systematic Review and Meta-Analysis of Clinical Trials. *Curr Hypertens Rep, 20*(11), 98. <https://doi.org/10.1007/s11906-018-0898-3>

Mirhosseini, N., Rainsbury, J., & Kimball, S. M. (2018). Vitamin D Supplementation, Serum 25(OH)D Concentrations and Cardiovascular Disease Risk Factors: A Systematic Review and Meta-Analysis. *Front Cardiovasc Med, 5*, 87. <https://doi.org/10.3389/fcvm.2018.00087>

Mohammadi, M., Ramezani-Jolfaie, N., Lorzadeh, E., Khoshbakht, Y., & Salehi-Abargouei, A. (2019, Mar). Hesperidin, a major flavonoid in orange juice, might not affect lipid profile and blood pressure: A systematic review and meta-analysis of randomized controlled clinical trials. *Phytother Res, 33*(3), 534-545. <https://doi.org/10.1002/ptr.6264>

Mohammadifard, N., Salehi-Abargouei, A., Salas-Salvado, J., Guasch-Ferre, M., Humphries, K., & Sarrafzadegan, N. (2015, May). The effect of tree nut, peanut, and soy nut consumption on blood pressure: a systematic review and meta-analysis of randomized controlled clinical trials. *Am J Clin Nutr, 101*(5), 966-982. <https://doi.org/10.3945/ajcn.114.091595>

Moraru, C., Mincea, M. M., Frandes, M., Timar, B., & Ostafe, V. (2018, Dec 12). A Meta-Analysis on Randomised Controlled Clinical Trials Evaluating the Effect of the Dietary Supplement Chitosan on Weight Loss, Lipid Parameters and Blood Pressure. *Medicina (Kaunas), 54*(6). <https://doi.org/10.3390/medicina54060109>

Morris, M. C., Sacks, F., & Rosner, B. (1993, Aug). Does fish oil lower blood pressure? A meta-analysis of controlled trials. *Circulation, 88*(2), 523-533. <https://doi.org/10.1161/01.cir.88.2.523>

Morze, J., Schwedhelm, C., Bencic, A., Hoffmann, G., Boeing, H., Przybylowicz, K., & Schwingshackl, L. (2020, Feb). Chocolate and risk of chronic disease: a systematic review and dose-response meta-analysis. *Eur J Nutr, 59*(1), 389-397. <https://doi.org/10.1007/s00394-019-01914-9>

Motallaei, M. R.-J., N. Mohammadi, M. Shams-Rad, S. Jahanlou, A. S. Salehi-Abargouei, A. (2021, Oct). Effects of orange juice intake on cardiovascular risk factors: A systematic review and meta-analysis of randomized controlled clinical trials. *Phytother Res, 35*(10), 5427-5439. <https://doi.org/10.1002/ptr.7173> UpdatedTo31Oct2021

Mousavi, S. M., Karimi, E., Hajishafiee, M., Milajerdi, A., Amini, M. R., & Esmaillzadeh, A. (2019, Oct 16). Anti-hypertensive effects of cinnamon supplementation in adults: A systematic review and dose-response Meta-analysis of randomized controlled trials. *Crit Rev Food Sci Nutr*, 1-11. <https://doi.org/10.1080/10408398.2019.1678012>

Naude, C. E., Schoonees, A., Senekal, M., Young, T., Garner, P., & Volmink, J. (2014, 2014). Low carbohydrate versus isoenergetic balanced diets for reducing weight and cardiovascular risk: a systematic review and meta-analysis. *PLoS One, 9*(7), e100652. <https://doi.org/10.1371/journal.pone.0100652>

Ndanuko, R. N. I., R. Hapsari, R. A. Neale, E. P. Raubenheimer, D. Charlton, K. E. (2021, Oct 1). Association between the Urinary Sodium to Potassium Ratio and Blood Pressure in Adults: A Systematic Review and Meta-Analysis. *Adv Nutr, 12*(5), 1751-1767. <https://doi.org/10.1093/advances/nmab036> UpdatedTo31Oct2021

Noordzij, M., Uiterwaal, C. S., Arends, L. R., Kok, F. J., Grobbee, D. E., & Geleijnse, J. M. (2005, May). Blood pressure response to chronic intake of coffee and caffeine: a meta-analysis of randomized controlled trials. *J Hypertens, 23*(5), 921-928. <https://doi.org/10.1097/01.hjh.0000166828.94699.1d>

O'Connor, L. E., Kim, J. E., & Campbell, W. W. (2017, Jan). Total red meat intake of >/=0.5 servings/d does not negatively influence cardiovascular disease risk factors: a systemically searched meta-analysis of randomized controlled trials. *Am J Clin Nutr, 105*(1), 57-69. <https://doi.org/10.3945/ajcn.116.142521>

Onakpoya, I., O'Sullivan, J., Heneghan, C., & Thompson, M. (2017, Feb 11). The effect of grapefruits (Citrus paradisi) on body weight and cardiovascular risk factors: A systematic review and meta-analysis of randomized clinical trials. *Crit Rev Food Sci Nutr, 57*(3), 602-612. <https://doi.org/10.1080/10408398.2014.901292>

Onakpoya, I. J., & Heneghan, C. J. (2015, Dec). Effect of the natural sweetener, steviol glycoside, on cardiovascular risk factors: a systematic review and meta-analysis of randomised clinical trials. *Eur J Prev Cardiol, 22*(12), 1575-1587. <https://doi.org/10.1177/2047487314560663>

Onakpoya, I. J., O'Sullivan, J., & Heneghan, C. J. (2015, May). The effect of cactus pear (Opuntia ficus-indica) on body weight and cardiovascular risk factors: a systematic review and meta-analysis of randomized clinical trials. *Nutrition, 31*(5), 640-646. <https://doi.org/10.1016/j.nut.2014.11.015>

Onakpoya, I. J., Spencer, E. A., Thompson, M. J., & Heneghan, C. J. (2015, Feb). The effect of chlorogenic acid on blood pressure: a systematic review and meta-analysis of randomized clinical trials. *J Hum Hypertens, 29*(2), 77-81. <https://doi.org/10.1038/jhh.2014.46>

Parohan, M., Sadeghi, A., Nasiri, M., Maleki, V., Khodadost, M., Pirouzi, A., & Sadeghi, O. (2019, Jul). Dietary acid load and risk of hypertension: A systematic review and dose-response meta-analysis of observational studies. *Nutr Metab Cardiovasc Dis, 29*(7), 665-675. <https://doi.org/10.1016/j.numecd.2019.03.009>

Picasso, M. C., Lo-Tayraco, J. A., Ramos-Villanueva, J. M., Pasupuleti, V., & Hernandez, A. V. (2019, Jun). Effect of vegetarian diets on the presentation of metabolic syndrome or its components: A systematic review and meta-analysis. *Clin Nutr, 38*(3), 1117-1132. <https://doi.org/10.1016/j.clnu.2018.05.021>

Pourmasoumi, M., Hadi, A., Najafgholizadeh, A., Joukar, F., & Mansour-Ghanaei, F. (2020, Mar). The effects of cranberry on cardiovascular metabolic risk factors: A systematic review and meta-analysis. *Clin Nutr, 39*(3), 774-788. <https://doi.org/10.1016/j.clnu.2019.04.003>

Pripp, A. H. (2008, 2008). Effect of peptides derived from food proteins on blood pressure: a meta-analysis of randomized controlled trials. *Food Nutr Res, 52*. <https://doi.org/10.3402/fnr.v52i0.1641>

Qi, D. N., X. L. Zhang, J. J. (2020, Apr 25). The effect of probiotics supplementation on blood pressure: a systemic review and meta-analysis. *Lipids Health Dis, 19*(1), 79. <https://doi.org/10.1186/s12944-020-01259-x> UpdatedTo31Oct2021

Ralston, R. A., Lee, J. H., Truby, H., Palermo, C. E., & Walker, K. Z. (2012, Jan). A systematic review and meta-analysis of elevated blood pressure and consumption of dairy foods. *J Hum Hypertens, 26*(1), 3-13. <https://doi.org/10.1038/jhh.2011.3>

Raman, G., Avendano, E. E., Chen, S., Wang, J., Matson, J., Gayer, B., Novotny, J. A., & Cassidy, A. (2019, Nov 1). Dietary intakes of flavan-3-ols and cardiometabolic health: systematic review and meta-analysis of randomized trials and prospective cohort studies. *Am J Clin Nutr, 110*(5), 1067-1078. <https://doi.org/10.1093/ajcn/nqz178>

Ramezani-Jolfaie, N., Mohammadi, M., & Salehi-Abargouei, A. (2019, Sep). The effect of healthy Nordic diet on cardio-metabolic markers: a systematic review and meta-analysis of randomized controlled clinical trials. *Eur J Nutr, 58*(6), 2159-2174. <https://doi.org/10.1007/s00394-018-1804-0>

Ramli, N. N. S. A., A. A. Mhd Jalil, A. M. (2021, Sep 11). Effects of Caffeinated and Decaffeinated Coffee Consumption on Metabolic Syndrome Parameters: A Systematic Review and Meta-Analysis of Data from Randomised Controlled Trials. *Medicina (Kaunas), 57*(9). <https://doi.org/10.3390/medicina57090957> UpdatedTo31Oct2021

Ran, L. Z., W. Tan, X. Wang, H. Mizuno, K. Takagi, K. Zhao, Y. Bu, H. (2020). Association between Serum Vitamin C and the Blood Pressure: A Systematic Review and Meta-Analysis of Observational Studies. *Cardiovasc Ther, 2020*, 4940673. <https://doi.org/10.1155/2020/4940673> UpdatedTo31Oct2021

Rebholz, C. M., Friedman, E. E., Powers, L. J., Arroyave, W. D., He, J., & Kelly, T. N. (2012, Oct 1). Dietary protein intake and blood pressure: a meta-analysis of randomized controlled trials. *Am J Epidemiol, 176 Suppl 7*, S27-43. <https://doi.org/10.1093/aje/kws245>

Ren, J. Y. A., J. Q. Chen, M. Y. Yang, H. Y. Ma, Y. X. (2021, Mar). Effect of proanthocyanidins on blood pressure: A systematic review and meta-analysis of randomized controlled trials [Review]. *Pharmacological Research, 165*, 10, Article 105329. <https://doi.org/10.1016/j.phrs.2020.105329> UpdatedTo31Oct2021

Reynolds, A., Mann, J., Cummings, J., Winter, N., Mete, E., & Te Morenga, L. (2019, Feb 2). Carbohydrate quality and human health: a series of systematic reviews and meta-analyses. *Lancet, 393*(10170), 434-445. <https://doi.org/10.1016/S0140-6736(18)31809-9>

Ried, K. (2016, Feb). Garlic Lowers Blood Pressure in Hypertensive Individuals, Regulates Serum Cholesterol, and Stimulates Immunity: An Updated Meta-analysis and Review. *J Nutr, 146*(2), 389S-396S. <https://doi.org/10.3945/jn.114.202192>

Ried, K., Fakler, P., & Stocks, N. P. (2017, Apr 25). Effect of cocoa on blood pressure. *Cochrane Database Syst Rev, 4*, CD008893. <https://doi.org/10.1002/14651858.CD008893.pub3>

Roerecke, M., Kaczorowski, J., Tobe, S. W., Gmel, G., Hasan, O. S. M., & Rehm, J. (2017, Feb). The effect of a reduction in alcohol consumption on blood pressure: a systematic review and meta-analysis. *Lancet Public Health, 2*(2), e108-e120. <https://doi.org/10.1016/S2468-2667(17)30003-8>

Roerecke, M., Tobe, S. W., Kaczorowski, J., Bacon, S. L., Vafaei, A., Hasan, O. S. M., Krishnan, R. J., Raifu, A. O., & Rehm, J. (2018, Jun 27). Sex-Specific Associations Between Alcohol Consumption and Incidence of Hypertension: A Systematic Review and Meta-Analysis of Cohort Studies. *J Am Heart Assoc, 7*(13). <https://doi.org/10.1161/JAHA.117.008202>

Sahebkar, A., Ferri, C., Giorgini, P., Bo, S., Nachtigal, P., & Grassi, D. (2017, Jan). Effects of pomegranate juice on blood pressure: A systematic review and meta-analysis of randomized controlled trials. *Pharmacol Res, 115*, 149-161. <https://doi.org/10.1016/j.phrs.2016.11.018>

Sahebkar, A., Soranna, D., Liu, X., Thomopoulos, C., Simental-Mendia, L. E., Derosa, G., Maffioli, P., & Parati, G. (2016, Nov). A systematic review and meta-analysis of randomized controlled trials investigating the effects of supplementation with Nigella sativa (black seed) on blood pressure. *J Hypertens, 34*(11), 2127-2135. <https://doi.org/10.1097/HJH.0000000000001049>

Santesso, N., Akl, E. A., Bianchi, M., Mente, A., Mustafa, R., Heels-Ansdell, D., & Schunemann, H. J. (2012, Jul). Effects of higher- versus lower-protein diets on health outcomes: a systematic review and meta-analysis. *Eur J Clin Nutr, 66*(7), 780-788. <https://doi.org/10.1038/ejcn.2012.37>

Schwingshackl, L., & Hoffmann, G. (2013, Apr 15). Long-term effects of low-fat diets either low or high in protein on cardiovascular and metabolic risk factors: a systematic review and meta-analysis. *Nutr J, 12*, 48. <https://doi.org/10.1186/1475-2891-12-48>

Schwingshackl, L., Schwedhelm, C., Hoffmann, G., & Boeing, H. (2019, Sep). Potatoes and risk of chronic disease: a systematic review and dose-response meta-analysis. *Eur J Nutr, 58*(6), 2243-2251. <https://doi.org/10.1007/s00394-018-1774-2>

Schwingshackl, L., Schwedhelm, C., Hoffmann, G., Knuppel, S., Iqbal, K., Andriolo, V., Bechthold, A., Schlesinger, S., & Boeing, H. (2017, Nov). Food Groups and Risk of Hypertension: A Systematic Review and Dose-Response Meta-Analysis of Prospective Studies. *Adv Nutr, 8*(6), 793-803. <https://doi.org/10.3945/an.117.017178>

Schwingshackl, L., Strasser, B., & Hoffmann, G. (2011). Effects of monounsaturated fatty acids on cardiovascular risk factors: a systematic review and meta-analysis. *Ann Nutr Metab, 59*(2-4), 176-186. <https://doi.org/10.1159/000334071>

Serban, C., Sahebkar, A., Ursoniu, S., Andrica, F., & Banach, M. (2015, Jun). Effect of sour tea (Hibiscus sabdariffa L.) on arterial hypertension: a systematic review and meta-analysis of randomized controlled trials. *J Hypertens, 33*(6), 1119-1127. <https://doi.org/10.1097/HJH.0000000000000585>

Setayesh, L. A.-L., D. Clark, C. C. T. Rezaei Kelishadi, M. Khalili, P. Bagheri, R. Asbaghi, O. Suzuki, K. (2021, Aug 9). The Effect of Saffron Supplementation on Blood Pressure in Adults: A Systematic Review and Dose-Response Meta-Analysis of Randomized Controlled Trials. *Nutrients, 13*(8). <https://doi.org/10.3390/nu13082736> UpdatedTo31Oct2021

Shah, M., Adams-Huet, B., & Garg, A. (2007, May). Effect of high-carbohydrate or high-cis-monounsaturated fat diets on blood pressure: a meta-analysis of intervention trials. *Am J Clin Nutr, 85*(5), 1251-1256. <https://doi.org/10.1093/ajcn/85.5.1251>

Shin, J. Y., Kim, J. Y., Kang, H. T., Han, K. H., & Shim, J. Y. (2015, 2015). Effect of fruits and vegetables on metabolic syndrome: a systematic review and meta-analysis of randomized controlled trials. *Int J Food Sci Nutr, 66*(4), 416-425. <https://www.tandfonline.com/doi/pdf/10.3109/09637486.2015.1025716?needAccess=true>

Shrime, M. G., Bauer, S. R., McDonald, A. C., Chowdhury, N. H., Coltart, C. E., & Ding, E. L. (2011, Nov). Flavonoid-rich cocoa consumption affects multiple cardiovascular risk factors in a meta-analysis of short-term studies. *J Nutr, 141*(11), 1982-1988. <https://doi.org/10.3945/jn.111.145482>

Soedamah-Muthu, S. S., Verberne, L. D., Ding, E. L., Engberink, M. F., & Geleijnse, J. M. (2012, Nov). Dairy consumption and incidence of hypertension: a dose-response meta-analysis of prospective cohort studies. *Hypertension, 60*(5), 1131-1137. <https://doi.org/10.1161/HYPERTENSIONAHA.112.195206>

Sohouli, M. H. L., A. Fatahi, S. Shidfar, F. Gaman, M. A. Guimaraes, N. S. Sindi, G. A. Mandili, R. A. Alzahrani, G. R. Abdulwahab, R. A. Almuflihi, A. M. Alsobyani, F. M. Mahmud, A. M. A. Nazzal, O. Alshaibani, L. Elmokid, S. Abu-Zaid, A. (2021, Aug). Impact of soy milk consumption on cardiometabolic risk factors: A systematic review and meta-analysis of randomized controlled trials [Review]. *Journal of Functional Foods, 83*, 13, Article 104499. <https://doi.org/10.1016/j.jff.2021.104499> UpdatedTo31Oct2021

Spaggiari, G. C., A. Sansone, A. Baldi, M. Santi, D. (2020). To beer or not to beer: A meta-analysis of the effects of beer consumption on cardiovascular health. *PLoS One, 15*(6), e0233619. <https://doi.org/10.1371/journal.pone.0233619> UpdatedTo31Oct2021

Steffen, M., Kuhle, C., Hensrud, D., Erwin, P. J., & Murad, M. H. (2012, Dec). The effect of coffee consumption on blood pressure and the development of hypertension: a systematic review and meta-analysis. *J Hypertens, 30*(12), 2245-2254. <https://doi.org/10.1097/HJH.0b013e3283588d73>

Suksomboon, N., Poolsup, N., & Lin, W. (2019, 2019). Effect of kiwifruit on metabolic health in patients with cardiovascular risk factors: a systematic review and meta-analysis. *Diabetes Metab Syndr Obes, 12*, 171-180. <https://www.dovepress.com/getfile.php?fileID=47683>

Tabrizi, R., Lankarani, K. B., Akbari, M., Naghibzadeh-Tahami, A., Alizadeh, H., Honarvar, B., Sharifi, N., Mazoochi, M., Ostadmohammadi, V., Fatholahpour, A., & Asemi, Z. (2018, May). The effects of folate supplementation on lipid profiles among patients with metabolic diseases: A systematic review and meta-analysis of randomized controlled trials. *Diabetes Metab Syndr, 12*(3), 423-430. <https://doi.org/10.1016/j.dsx.2017.12.022>

Te Morenga, L. A., Howatson, A. J., Jones, R. M., & Mann, J. (2014, Jul). Dietary sugars and cardiometabolic risk: systematic review and meta-analyses of randomized controlled trials of the effects on blood pressure and lipids. *Am J Clin Nutr, 100*(1), 65-79. <https://doi.org/10.3945/ajcn.113.081521>

Teoh, S. L., Lai, N. M., Vanichkulpitak, P., Vuksan, V., Ho, H., & Chaiyakunapruk, N. (2018, Apr 1). Clinical evidence on dietary supplementation with chia seed (Salvia hispanica L.): a systematic review and meta-analysis. *Nutr Rev, 76*(4), 219-242. <https://doi.org/10.1093/nutrit/nux071>

Tierney, A. C. R., C. E. Billings, L. M. George, E. S. (2020, Nov 16). Effect of Dietary and Supplemental Lycopene on Cardiovascular Risk Factors: A Systematic Review and Meta-Analysis. *Adv Nutr, 11*(6), 1453-1488. <https://doi.org/10.1093/advances/nmaa069> UpdatedTo31Oct2021

Toh, D. W. K. K., E. S. Kim, J. E. (2020, Jul 1). Incorporating healthy dietary changes in addition to an increase in fruit and vegetable intake further improves the status of cardiovascular disease risk factors: A systematic review, meta-regression, and meta-analysis of randomized controlled trials. *Nutr Rev, 78*(7), 532-545. <https://doi.org/10.1093/nutrit/nuz104> UpdatedTo31Oct2021

Ursoniu, S., Sahebkar, A., Andrica, F., Serban, C., Banach, M., Lipid, & Blood Pressure Meta-analysis Collaboration, G. (2016, Jun). Effects of flaxseed supplements on blood pressure: A systematic review and meta-analysis of controlled clinical trial. *Clin Nutr, 35*(3), 615-625. <https://doi.org/10.1016/j.clnu.2015.05.012>

Wang, C. J., Shen, Y. X., & Liu, Y. (2016, 2016). Empirically Derived Dietary Patterns and Hypertension Likelihood: A Meta-Analysis. *Kidney Blood Press Res, 41*(5), 570-581.

Wang, Y. X. L., J. J. Zhao, C. Tian, H. B. Geng, Y. M. Sun, L. L. Ma, X. Y. Wang, Y. Zhang, R. H. Zheng, X. T. Chen, X. M. (2020, Mar). The effect of tomato on weight, body mass index, blood pressure and inflammatory factors: A systematic review and dose-response meta-analysis of randomized controlled trials [Review]. *Journal of King Saud University Science, 32*(2), 1619-1627. <https://doi.org/10.1016/j.jksus.2019.12.020> UpdatedTo31Oct2021

Weaver, S. R. R., C. McGettrick, H. M. Philp, A. Lucas, S. J. E. (2021, Feb). Fine wine or sour grapes? A systematic review and meta-analysis of the impact of red wine polyphenols on vascular health. *Eur J Nutr, 60*(1), 1-28. <https://doi.org/10.1007/s00394-020-02247-8> UpdatedTo31Oct2021

Wendland, E., Farmer, A., Glasziou, P., & Neil, A. (2006, Feb). Effect of alpha linolenic acid on cardiovascular risk markers: a systematic review. *Heart, 92*(2), 166-169.

Whelton, P. K., He, J., Cutler, J. A., Brancati, F. L., Appel, L. J., Follmann, D., & Klag, M. J. (1997). Effects of Oral Potassium on Blood Pressure: Meta-analysis of Randomized Controlled Clinical Trials. *JAMA, 277*(20), 1624-1632. <https://doi.org/10.1001/jama.1997.03540440058033>

Whelton, S. P., Hyre, A. D., Pedersen, B., Yi, Y., Whelton, P. K., & He, J. (2005, Mar). Effect of dietary fiber intake on blood pressure: a meta-analysis of randomized, controlled clinical trials. *J Hypertens, 23*(3), 475-481. <https://doi.org/10.1097/01.hjh.0000160199.51158.cf>

Wu, L., & Sun, D. (2017, Sep). Effects of calcium plus vitamin D supplementation on blood pressure: a systematic review and meta-analysis of randomized controlled trials. *J Hum Hypertens, 31*(9), 547-554. <https://doi.org/10.1038/jhh.2017.12>

Wu, L., Sun, D., & He, Y. (2016, Oct). Fruit and vegetables consumption and incident hypertension: dose-response meta-analysis of prospective cohort studies. *J Hum Hypertens, 30*(10), 573-580. <https://doi.org/10.1038/jhh.2016.44>

Xia, W. T., N. Kord-Varkaneh, H. Low, T. Y. Tan, S. C. Wu, X. Zhu, Y. (2020, Nov). The effects of astaxanthin supplementation on obesity, blood pressure, CRP, glycemic biomarkers, and lipid profile: A meta-analysis of randomized controlled trials. *Pharmacol Res, 161*, 105113. <https://doi.org/10.1016/j.phrs.2020.105113> UpdatedTo31Oct2021

Xie, C., Cui, L., Zhu, J., Wang, K., Sun, N., & Sun, C. (2018, Feb). Coffee consumption and risk of hypertension: a systematic review and dose-response meta-analysis of cohort studies. *J Hum Hypertens, 32*(2), 83-93. <https://doi.org/10.1038/s41371-017-0007-0>

Xu, R. Y., K. Ding, J. Chen, G. (2020, Feb). Effect of green tea supplementation on blood pressure: A systematic review and meta-analysis of randomized controlled trials. *Medicine (Baltimore), 99*(6), e19047. <https://doi.org/10.1097/md.0000000000019047> UpdatedTo31Oct2021

Yang, B., Shi, M. Q., Li, Z. H., Yang, J. J., & Li, D. (2016, Jan 21). Fish, Long-Chain n-3 PUFA and Incidence of Elevated Blood Pressure: A Meta-Analysis of Prospective Cohort Studies. *Nutrients, 8*(1). <https://doi.org/10.3390/nu8010058>

Yang, J., Wang, H. P., Zhou, L. M., Zhou, L., Chen, T., & Qin, L. Q. (2015, Feb 18). Effect of conjugated linoleic acid on blood pressure: a meta-analysis of randomized, double-blind placebo-controlled trials. *Lipids Health Dis, 14*, 11. <https://doi.org/10.1186/s12944-015-0010-9>

Yang, Q. L., X. Li, W. Liang, Y. (2021, Jun 24). The effects of low-fat, high-carbohydrate diets vs. low-carbohydrate, high-fat diets on weight, blood pressure, serum liquids and blood glucose: a systematic review and meta-analysis. *Eur J Clin Nutr*. <https://doi.org/10.1038/s41430-021-00927-0> UpdatedTo31Oct2021

Zamora-Zamora, F., Martinez-Galiano, J. M., Gaforio, J. J., & Delgado-Rodriguez, M. (2018, Oct-Dec). Effects of olive oil on blood pressure: A systematic review and meta-analysis. *Grasas Y Aceites, 69*(4). <https://doi.org/ARTN> e272

10.3989/gya.0105181

Zhang, X., Li, Y., Del Gobbo, L. C., Rosanoff, A., Wang, J., Zhang, W., & Song, Y. (2016, Aug). Effects of Magnesium Supplementation on Blood Pressure: A Meta-Analysis of Randomized Double-Blind Placebo-Controlled Trials. *Hypertension, 68*(2), 324-333. <https://doi.org/10.1161/HYPERTENSIONAHA.116.07664>

Zhang, Y., & Zhang, D.-z. (2018, 2018/07/01). Red meat, poultry, and egg consumption with the risk of hypertension: a meta-analysis of prospective cohort studies. *Journal of Human Hypertension, 32*(7), 507-517. <https://doi.org/10.1038/s41371-018-0068-8>

Ziaei, R. A., G. Foshati, S. Zolfaghari, H. Clark, C. C. T. Rouhani, M. H. (2020). Association between urinary potassium excretion and blood pressure: A systematic review and meta-analysis of observational studies. *J Res Med Sci, 25*, 116. <https://doi.org/10.4103/jrms.JRMS_167_20> UpdatedTo31Oct2021
